# Supplementary material for: Single cell T cell landscape and T cell receptor repertoire profiling of AML in context of PD-1 blockade therapy
Source: Nat Commun. 2021 Oct 18;12:6071. doi: 10.1038/s41467-021-26282-z (PMC8524723; doi:10.1038/s41467-021-26282-z)
Supplement: Supplementary file 1 — Supplementary Information [file 41467_2021_26282_MOESM1_ESM.pdf]

Single Cell T Cell Landscape and T Cell Receptor Repertoire Profiling of AML in Context of PD-1 Blockade Therapy

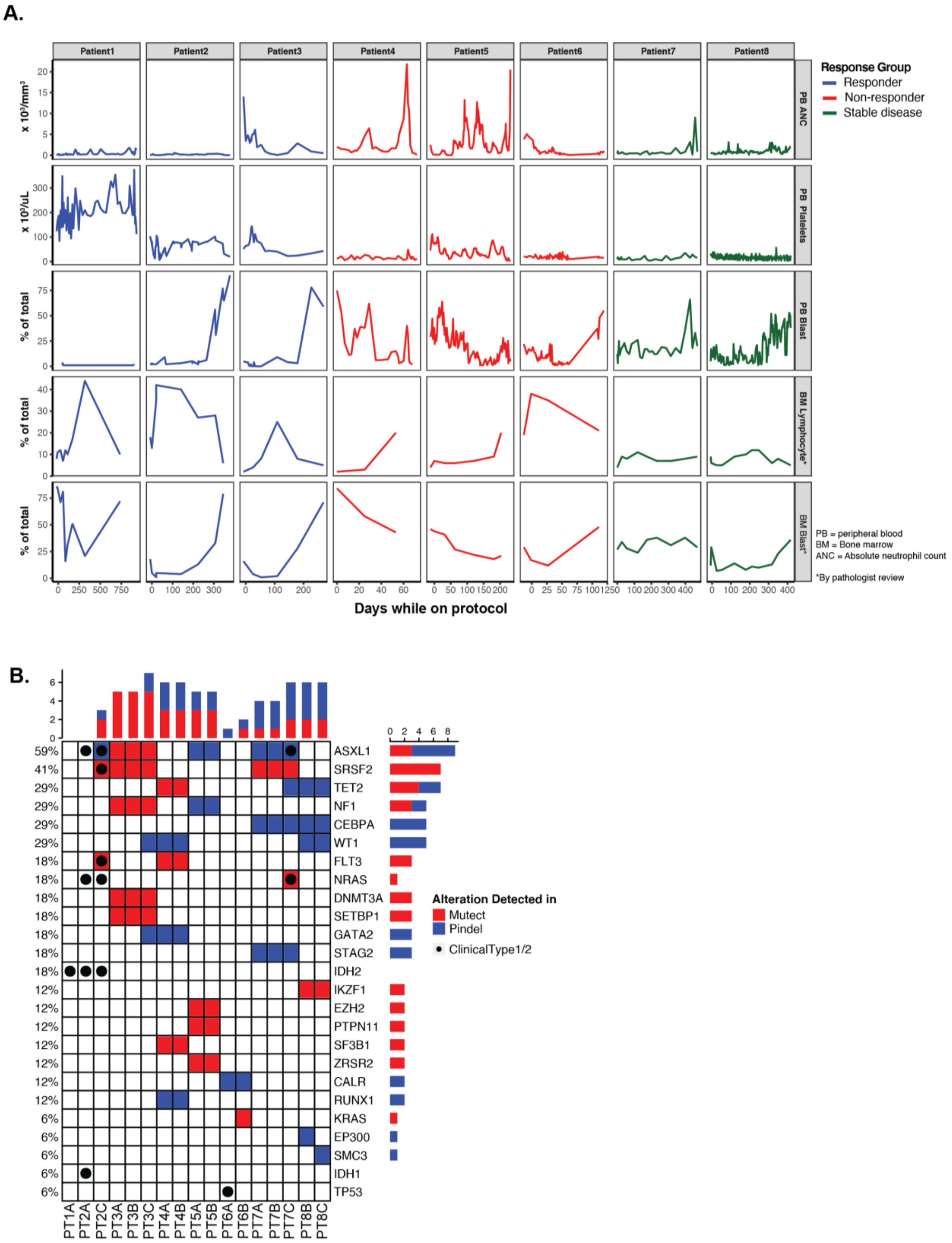

A.
Patient1
Patient2
Patient3
Patient4
Patient5
Patient6
Patient7
Patient8
Response Group
Responder
Non-responder
Stable disease
PB ANC
PB Platelets
PB Blast
BM Lymphocyte*
BM Blast*
x 10³/mm³
x 10³/uL
% of total
PB = peripheral blood
BM = Bone marrow
ANC = Absolute neutrophil count
*By pathologist review
Days while on protocol
B.
ASXL1
SRSF2
TET2
NF1
CEBPA
WT1
FLT3
NRAS
DNMT3A
SETBP1
GATA2
STAG2
IDH2
IKZF1
EZH2
PTPN11
SF3B1
ZRSR2
CALR
RUNX1
KRAS
EP300
SMC3
IDH1
TP53
59%
41%
29%
29%
29%
29%
18%
18%
18%
18%
18%
18%
18%
12%
12%
12%
12%
12%
12%
12%
6%
6%
6%
6%
6%
Alteration Detected in
Mutect
Pindel
ClinicalType1/2
PT1A
PT2A
PT2C
PT3A
PT3B
PT3C
PT4A
PT4B
PT5A
PT5B
PT6A
PT6B
PT7A
PT7B
PT7C
PT8B
PT8C

Supplementary Figure 1. (A) Bone marrow (BM) and peripheral blood (PB) laboratory results while on azacitidine/nivolumab protocol. (b) Targeted DNA sequencing of recurrent mutations in hematologic malignancies [96] as well as a CLIA-certified molecular diagnostic assay at the different treatment timepoints when available.

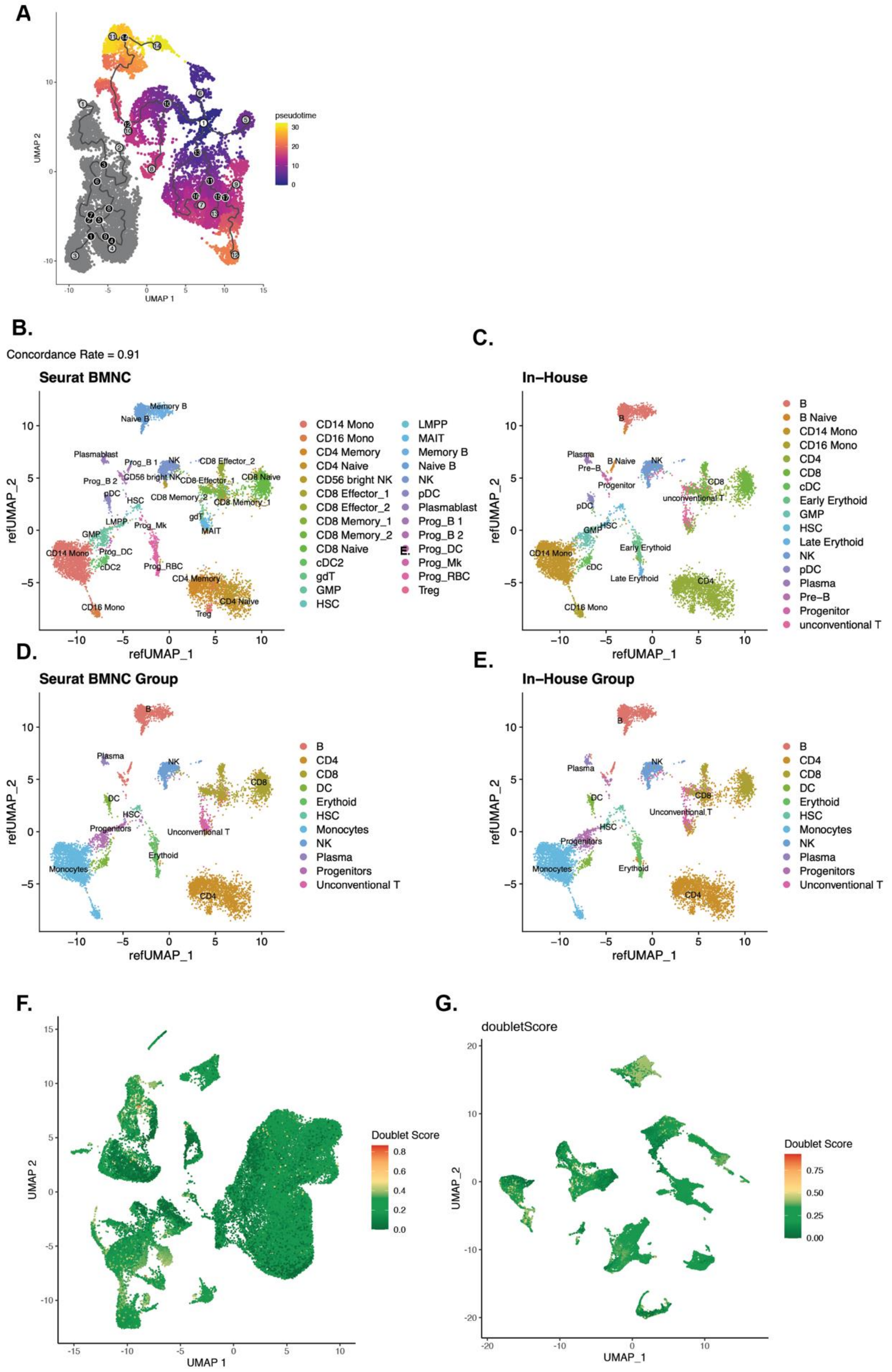
A
pseudotime
UMAP 1
UMAP 2
B.
Concordance Rate = 0.91
Seurat BMNC
refUMAP_1
refUMAP_2
CD14 Mono
CD16 Mono
CD4 Memory
CD4 Naive
CD56 bright NK
CD8 Effector_1
CD8 Effector_2
CD8 Memory_1
CD8 Memory_2
CD8 Naive
cDC2
gdT
GMP
HSC
LMPP
MAIT
Memory B
Naive B
NK
pDC
Plasmablast
Prog_B 1
Prog_B 2
Prog_DC
Prog_Mk
Prog_RBC
Treg
C.
In–House
B
B Naive
CD14 Mono
CD16 Mono
CD4
CD8
cDC
Early Erythoid
GMP
HSC
Late Erythoid
NK
pDC
Plasma
Pre–B
Progenitor
unconventional T
D.
Seurat BMNC Group
B
CD4
CD8
DC
Erythoid
HSC
Monocytes
NK
Plasma
Progenitors
Unconventional T
E.
In–House Group
B
CD4
CD8
DC
Erythoid
HSC
Monocytes
NK
Plasma
Progenitors
Unconventional T
F.
Doublet Score
UMAP 1
UMAP 2
G.
doubletScore
Doublet Score
UMAP_1
UMAP_2

Supplementary Figure 2. (A) Pseudotemporal trajectory analysis of the normal BM components using monocle3[40]. Mapping of healthy bone marrow cells to publicly available multimodal human BMNC reference with annotation based on public reference (B and D) versus our group's annotation (C and E) demonstrating high concordance of 91% among cells. (F-G) DoubletFinder[45] doublet scores demonstrating very low rate of doublets after our quality control assessment.

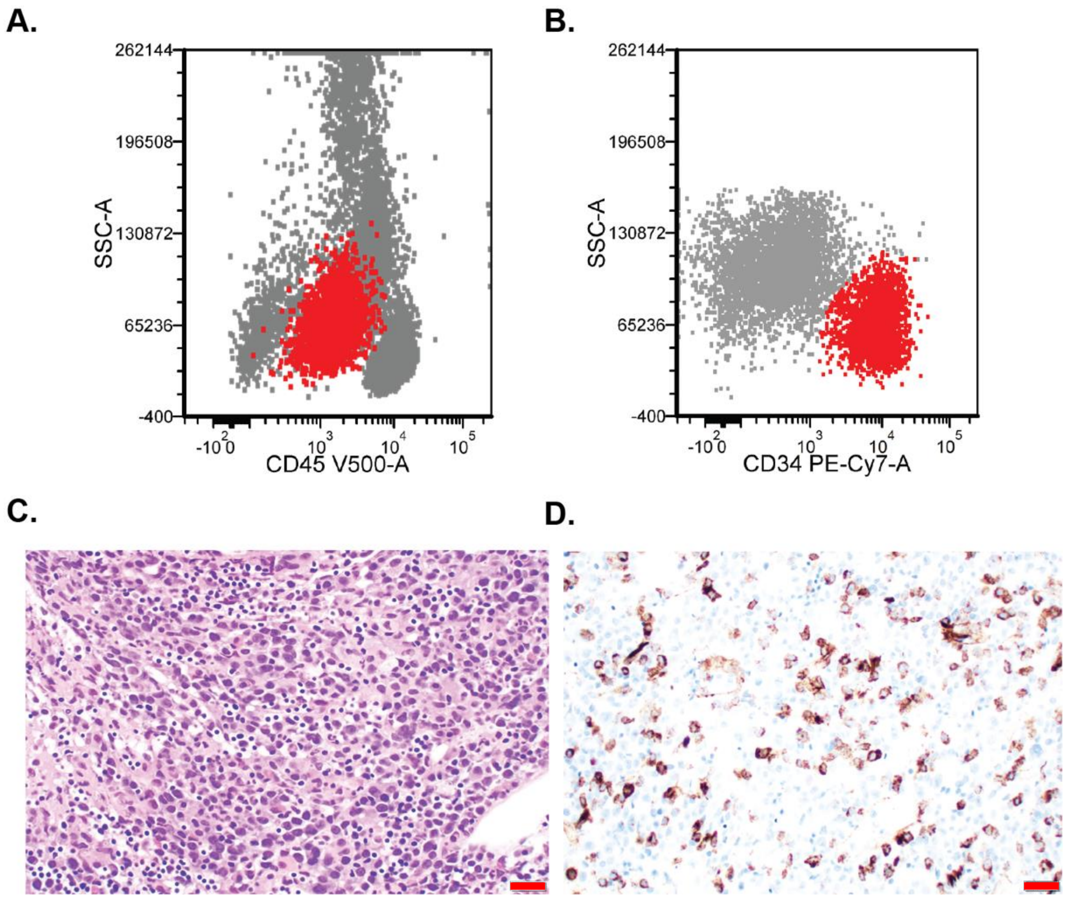

Supplementary Figure 3. (A-B) Starting from all events the flow cytometer collected, a gate for singlets is made to first exclude all doublets. Under Singlets gate, a FSC/SSC gate is drawn to exclude debris. Then a gate for nucleated cells is drawn to exclude mature red cells. Under nucleated cells gate, a CD45 dim gate is mad. Blast population (CD34-positive blasts) is under CD45 dim gate. (C) Hematoxylin and eosin staining of a representative BM biopsy. (D) Immunohistochemistry with CD34 antibody demonstrating CD34 positive blasts. Scalebar represents 20 um. All 22 AML bone marrows had uniform characterization of blast population by hematopathologists as represented in Figures A to D.

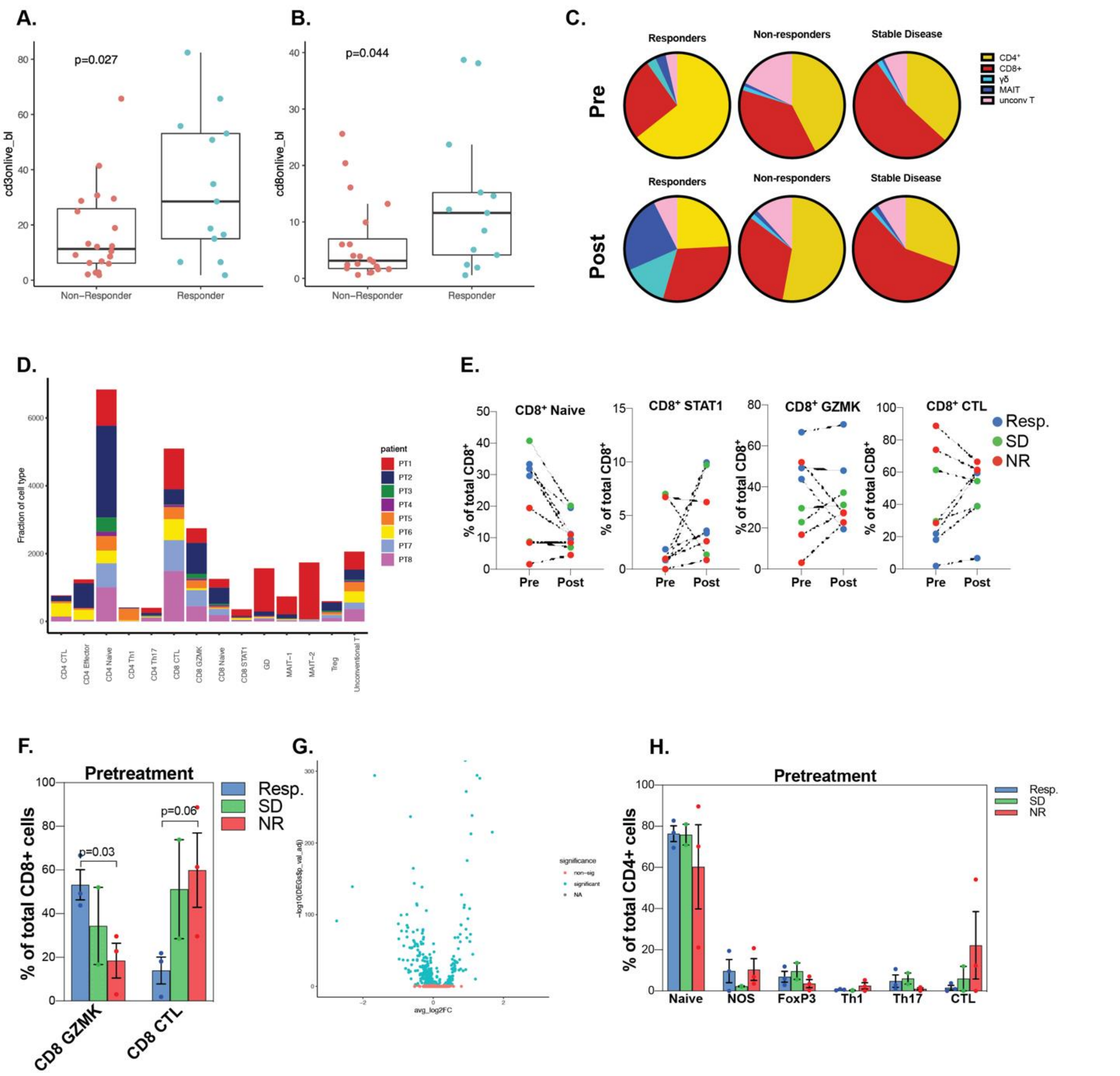

Supplementary Figure 4. T cell subset distributions across different patient populations and timepoints. Frequency of (A) CD3+ and (B) CD8+ cells from responders (n=13) and non-responders (n=20) as measured by flow cytometry at time of enrollment in study. The lower and upper hinges correspond to the first and third quartiles. (C) Distribution of T-cell subsets in pre and post treatment timepoints. (D) Contribution of each patient to the T cell subsets. (E) Pre- and post- treatment distribution change in CD8 subsets. (F) Pretreatment levels of CD8 GZMK and CD8 CTL in different response groups (responder n=3; stable disease n=2; non-responder n=3).

(G) Differentially expression of genes in CD8 GZMK subset between responders and non-responders. (H) Distribution of CD4 subsets in the response groups at pre-treatment (responder n=3; stable disease n=2; non-responder n=3). Error bars represent standard error of the mean.

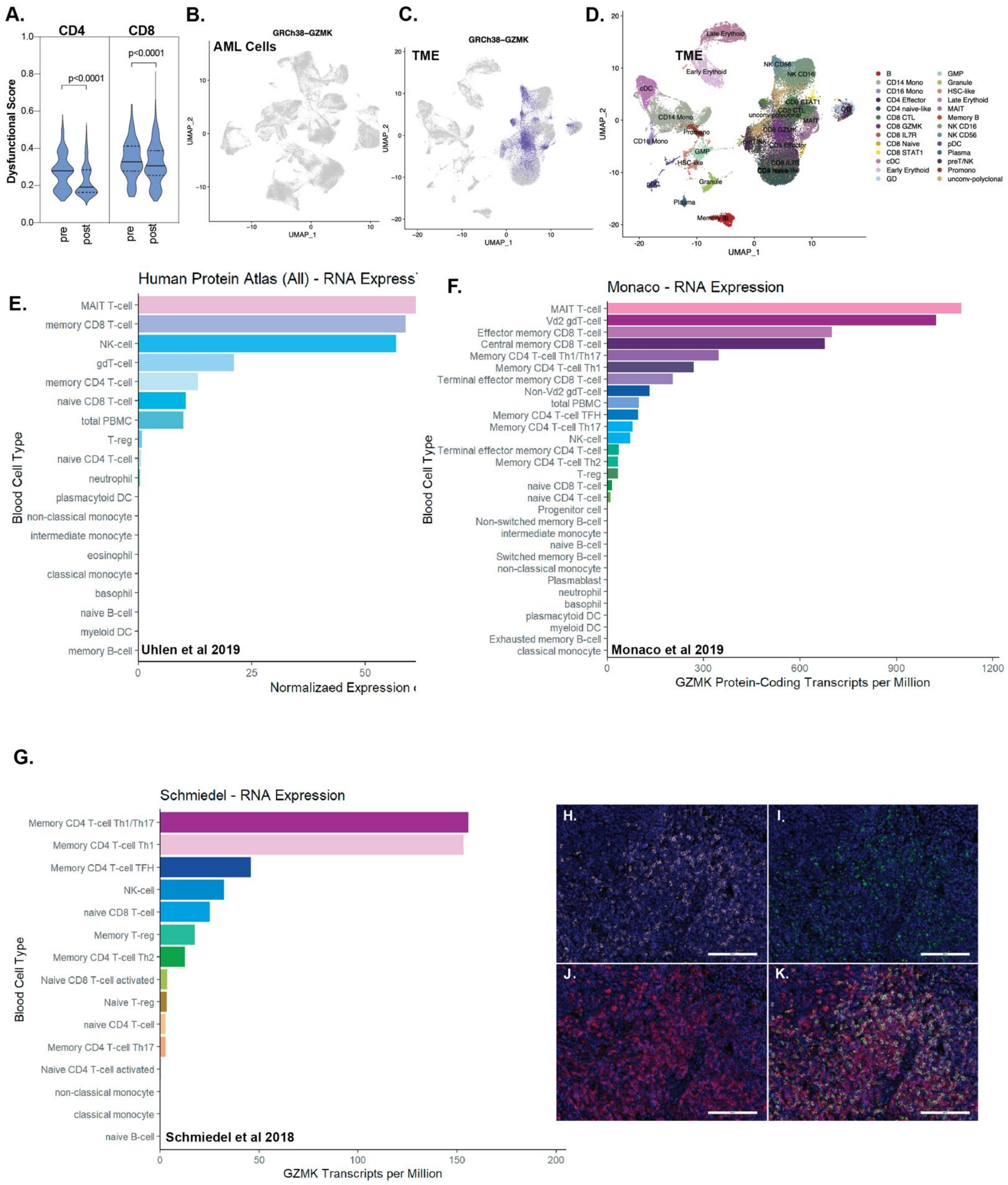

A.
CD4
CD8
p<0.0001
p<0.0001
Dysfunctional Score
1.0
0.8
0.6
0.4
0.2
0.0
pre
post
pre
post
B.
GRCh38–GZMK
AML Cells
UMAP_1
UMAP_2
C.
GRCh38–GZMK
TME
UMAP_1
UMAP_2
D.
TME
UMAP_1
UMAP_2
Late Erythoid
Early Erythoid
cDC
CD14 Mono
CD16 Mono
Promono
GMP
HSC-like
pDC
Granule
Plasma
Memory B
NK CD56
NK CD16
CD8 STAT1
unconv-polyclonal
CD8 CTL
CD8 GZMK
MAIT
CD4 Effector
CD8 IL7R
CD8 Naive
GD
B
CD14 Mono
CD16 Mono
CD4 Effector
CD4 naive-like
CD8 CTL
CD8 GZMK
CD8 IL7R
CD8 Naive
CD8 STAT1
cDC
Early Erythoid
GD
GMP
Granule
HSC-like
Late Erythoid
MAIT
Memory B
NK CD16
NK CD56
pDC
Plasma
preT/NK
Promono
unconv-polyclonal
E.
Human Protein Atlas (All) - RNA Express
Blood Cell Type
MAIT T-cell
memory CD8 T-cell
NK-cell
gdT-cell
memory CD4 T-cell
naive CD8 T-cell
total PBMC
T-reg
naive CD4 T-cell
neutrophil
plasmacytoid DC
non-classical monocyte
intermediate monocyte
eosinophil
classical monocyte
basophil
naive B-cell
myeloid DC
memory B-cell
Uhlen et al 2019
0
25
50
Normalizaed Expression
F.
Monaco - RNA Expression
Blood Cell Type
MAIT T-cell
Vd2 gdT-cell
Effector memory CD8 T-cell
Central memory CD8 T-cell
Memory CD4 T-cell Th1/Th17
Memory CD4 T-cell Th1
Terminal effector memory CD8 T-cell
Non-Vd2 gdT-cell
total PBMC
Memory CD4 T-cell TFH
Memory CD4 T-cell Th17
NK-cell
Terminal effector memory CD4 T-cell
Memory CD4 T-cell Th2
T-reg
naive CD8 T-cell
naive CD4 T-cell
Progenitor cell
Non-switched memory B-cell
intermediate monocyte
naive B-cell
Switched memory B-cell
non-classical monocyte
Plasmablast
neutrophil
basophil
plasmacytoid DC
myeloid DC
Exhausted memory B-cell
classical monocyte
Monaco et al 2019
0
300
600
900
1200
GZMK Protein-Coding Transcripts per Million
G.
Schmiedel - RNA Expression
Blood Cell Type
Memory CD4 T-cell Th1/Th17
Memory CD4 T-cell Th1
Memory CD4 T-cell TFH
NK-cell
naive CD8 T-cell
Memory T-reg
Memory CD4 T-cell Th2
Naive CD8 T-cell activated
Naive T-reg
naive CD4 T-cell
Memory CD4 T-cell Th17
Naive CD4 T-cell activated
non-classical monocyte
classical monocyte
naive B-cell
Schmiedel et al 2018
0
50
100
150
200
GZMK Transcripts per Million
H.
I.
J.
K.

Supplementary Figure 5. Expression of GZMK across different cell types and tissues. (A) Exhaustion scores in CD4 and CD8 subsets in non-responders at pre- and following treatment. Center line represent the median. GZMK expression in (B) AML cells and (C) tumor microenvironment. (D) Cellular annotation of tumor microenvironment components. (E-G): Expression of GZMK in 3 independent datasets from Human Protein Atlas. [5758-60] H) CD8 Immunofluorescence stain show reactivity (pink) in CD8 T-cells; I) GZMK immunofluorescence stain reveal positivity (green) in a subset of lymphocytes; J) CD45RO Immunofluorescence stain shows positivity (red) in memory and CD8 T-cells; K) Composite image multiplexing all markers used in a panel shows a co-expression of CD8, GZMK and CD45RO in a subset of cells. Scalebar represents 200 um.

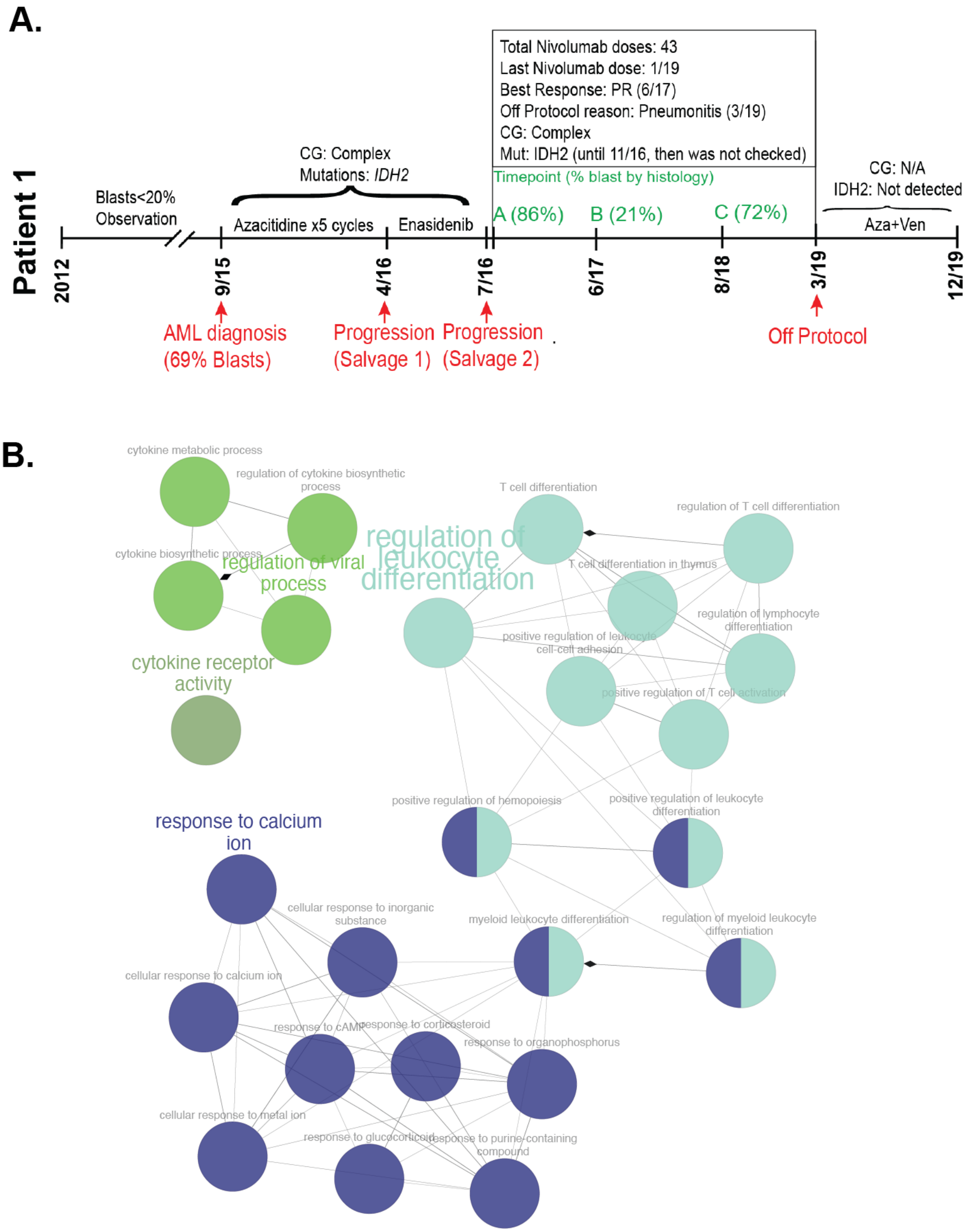
A.
Patient 1
Total Nivolumab doses: 43
Last Nivolumab dose: 1/19
Best Response: PR (6/17)
Off Protocol reason: Pneumonitis (3/19)
CG: Complex
Mut: IDH2 (until 11/16, then was not checked)
Timepoint (% blast by histology)
A (86%)
B (21%)
C (72%)
CG: Complex
Mutations: IDH2
Blasts<20%
Observation
Azacitidine x5 cycles
Enasidenib
CG: N/A
IDH2: Not detected
Aza+Ven
2012
9/15
4/16
7/16
6/17
8/18
3/19
12/19
AML diagnosis
(69% Blasts)
Progression
(Salvage 1)
Progression
(Salvage 2)
Off Protocol
B.
cytokine metabolic process
regulation of cytokine biosynthetic process
cytokine biosynthetic process
regulation of viral process
cytokine receptor activity
T cell differentiation
regulation of T cell differentiation
regulation of leukocyte differentiation
T cell differentiation in thymus
regulation of lymphocyte differentiation
positive regulation of leukocyte cell-cell adhesion
positive regulation of T cell activation
positive regulation of hemopoiesis
positive regulation of leukocyte differentiation
response to calcium ion
cellular response to inorganic substance
myeloid leukocyte differentiation
regulation of myeloid leukocyte differentiation
cellular response to calcium ion
response to cAMP
response to corticosteroid
response to organophosphorus
cellular response to metal ion
response to glucocorticoid
response to purine-containing compound

Supplementary Figure 6. Clinical course of patient 1 and CD8 GZMK enriched pathways (A) Clinical course of PT1 (responder). (B) ClueGo[97] plot of pathways enriched in GZMK-expressing cells.

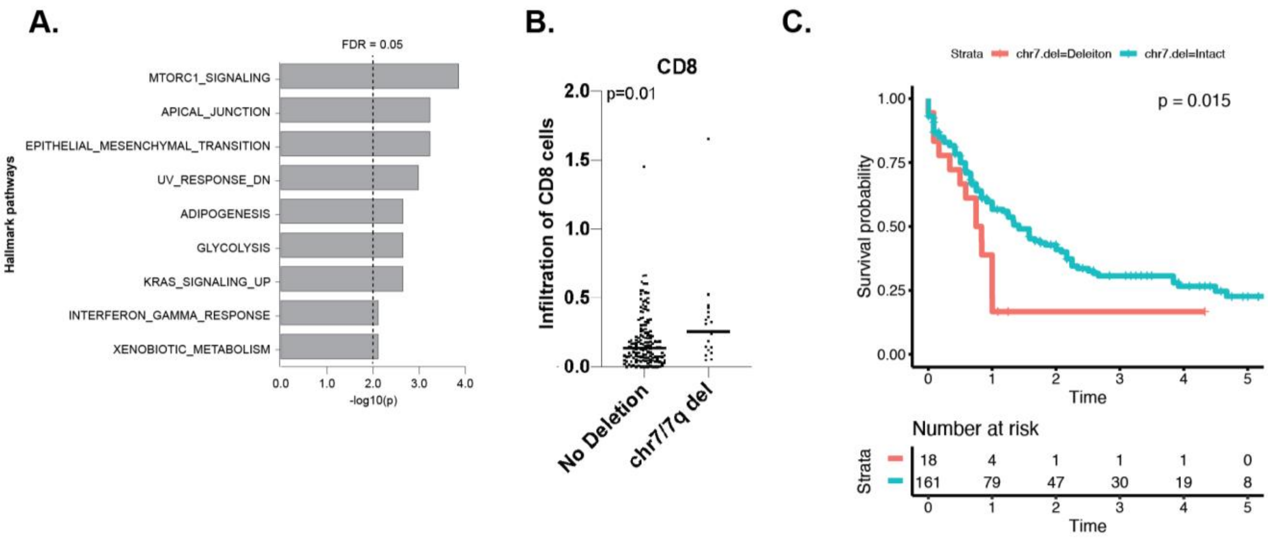

Supplementary Figure 7. Chr7/7q pathways and correlation with CD8 infiltration and AML outcomes. (A) Pathway analysis of differential genes enriched on chr7q. (B) Infiltration of CD8 cells in AML based on TCGA cohort. Line represent values above and below false discovery rate (FDR) test for significance of 0.05 (C) Overall survival of AML patients by chr7/7q loss status in TCGA cohort. P-value of log-rank test.

Patient ID	Age	Prior HMA	Time to Response Assessment (Months)	Best Response	Off protocol reason	Pretreatment Cytogenetics
PT1	88	Yes	10.57	PR	Pneumonitis	75~90<4n>,XXYY,-3,-7,-8,-9,+12,-13,-14,-16,+19,-20,+22,+22,+mar[cp13]/46,XY[7
PT2	80	Yes	0.77	CR	Relapse	46,XY[20]
PT3	65	No	1.77	CR	Relapse	46,XY,del(12)(p12)[5]/46,XY[15]
PT4	72	Yes	2.37	NR	No response	46,XY,del(7)(q22)[20]
PT5	71	Yes	4.00	NR	No response	45,XY,-7[19]/46,XY[1]
PT6	64	Yes	3.97	NR	No response	45,XX,5q-,6p-,-17,der(20)add(20q),+mar[3]/46,XX,-5,6p-,-7,-17,19q+,der(20)add(20q),+2mar[7]
PT7	74	Yes	15.63	SD	Loss of clinical benefit	47,XY,+8[5]/47,idem,del(8)(q21.2q24.3)[8]/46,XY[7]
PT8	76	Yes	13.90	SD	Loss of clinical benefit	46,XY,t(3;14)(q21;q32)[1]/46,XY,-14,add(20)(q13.3),+mar[1]/46,XY[18]

*PR=partial response; CR=complete response; NR=no response; SD =stable disease

Supplementary Table 1. Clinical characteristics of patients 1 to 8 on combined nivolumab/azacitidine study.
