# Supplementary material for: Single cell T cell landscape and T cell receptor repertoire profiling of AML in context of PD-1 blockade therapy
Source: Nat Commun. 2021 Oct 18;12:6071. doi: 10.1038/s41467-021-26282-z (PMC8524723; doi:10.1038/s41467-021-26282-z)
Supplement: Supplementary file 3 — Description of Additional Supplementary Files [file 41467_2021_26282_MOESM3_ESM.pdf]

Description of Additional Supplementary Files

File Name: Supplementary Data 1
Description: Differential gene expression of CD8 GZMK cells between responders and non-responders

File Name: Supplementary Data 2
Description: Differential gene expression of genes on chr7q region between patients with intact chr7/7q and those with loss in chr7/7q

File Name: Supplementary Data 3
Description: Clinical protocol entitled "An Open-label Phase II Study of Nivolumab (BMS-936558) in Combination with 5-azacytidine (Vidaza) or Nivolumab with Ipilimumab in combination with 5-azacytidine for the Treatment of Patients with Refractory/Relapsed Acute Myeloid Leukemia and newly diagnosed AML (>=65 years patients). PI: Naval Daver and Jorge Cortes;
