# Supplementary material for: Single cell T cell landscape and T cell receptor repertoire profiling of AML in context of PD-1 blockade therapy
Source: Nat Commun. 2021 Oct 18;12:6071. doi: 10.1038/s41467-021-26282-z (PMC8524723; doi:10.1038/s41467-021-26282-z)
Supplement: Supplementary file 6 — Supplementary Note 1 [file 41467_2021_26282_MOESM6_ESM.pdf]

An Open-label Phase II Study of Nivolumab (BMS-936558) in Combination with 5-azacytidine (Vidaza) or Nivolumab with Ipilimumab in combination with 5-azacytidine for the Treatment of Patients with Refractory/ Relapsed Acute Myeloid Leukemia and newly diagnosed older AML (≥65 years) patients

Principal Investigator: Naval Daver, M.D. and Jorge Cortes, M.D.

Sponsor: University of Texas, MD Anderson Cancer Center

IND number:125,751

University of Texas

MD Anderson Cancer Center

Leukemia Department, Unit 428

1.0 OBJECTIVES

1.1 Primary Objectives

Part a. Lead-in phase:

1. To determine the maximum tolerated dose (MTD) and dose limiting toxicity (DLT) of nivolumab in combination with 5-azacytidine in patients with refractory/ relapsed acute myeloid leukemia (AML).
2. To determine the maximum tolerated dose (MTD) and dose limiting toxicity (DLT) of nivolumab with ipilimumab in combination with 5-azacytidine in patients with refractory/ relapsed acute myeloid leukemia (AML).

Part b. Phase II

1. To determine the overall response rate (ORR) of nivolumab in combination with 5-azacytidine in patients with refractory/ relapsed AML.
2. To determine the overall response rate (ORR) of nivolumab in combination with 5-azacytidine in older patients (≥65 years) with newly diagnosed AML.
3. To determine the overall response rate (ORR) of nivolumab with ipilimumab in combination with 5-azacytidine in patients with refractory/ relapsed AML.
4. To determine the overall response rate (ORR) of nivolumab with ipilimumab in combination with 5- azacytidine in older patients (≥65 years) with newly diagnosed AML.

1.2 Secondary Objectives:

1. To determine the number of patients who achieve a > 50% reduction in blasts on therapy with either vidaza+nivolumab or vidaza+nivolumab+ipilimumab.
2. To determine the duration of response, disease-free survival (DFS), and overall survival (OS) of patients with refractory/ relapsed AML treated with either vidaza+nivolumab or vidaza+nivolumab+ipilimumab.
3. To determine the duration of response, disease-free survival (DFS), and overall survival (OS) in older patients with newly diagnosed AML treated with either vidaza+nivolumab or vidaza+nivolumab+ipilimumab.

1.3 Exploratory Objectives:

1. To study immunological and molecular changes in the peripheral blood and bone marrow in response to nivolumab and 5-azacytidine therapy or nivolumab with ipilimumab and 5-azacutidine therapy.
2. To determine induction of hypomethylation and DNA damage during therapy with this combination and its correlation with response.

2.0 BACKGROUND

2.1 Acute myeloid leukemia

AML is a malignancy of immature granulocytes or monocytes. The malignancy is characterized by accumulation of leukemic blastocytes and blockade of normal bone marrow production resulting in thrombocytopenia, anemia, and neutropenia. . There are approximately 13,000 new cases of AML per year in the United States, with an estimated

10,000 deaths occurring in the same time period[1]. Almost all newly diagnosed cases, as well as deaths, will be in adults[2]. Standard treatment for AML includes systemic combination chemotherapy to control bone marrow and systemic disease. Treatment is generally divided into an induction phase, to attain remission, and a maintenance phase[2]. Approximately 60% to 70% of adults with AML can be expected to attain complete remission status following appropriate induction therapy. Remission rates in adult AML are inversely related to age, with an expected remission rate of >65% for those younger than 60 years. Increased morbidity and mortality during induction appear to be directly related to age[3-5].

2.2A Relapsed/ Refractory AML

Approximately, 30-40% of adults with AML fail to achieve CR with 1 or 2 cycles of induction chemotherapy, and are deemed primary refractory. The outcome of patients with acute myeloid leukemia (AML) who are refractory to induction therapy are dismal, with low response rates to salvage chemotherapy and poor long-term survival [6-8]. We have previously reported a dismal median OS of 3.8 months for patients with AML who are refractory to HiDAC-containing induction therapy (defined as $\geq$ 1gm/m^2 cytarabine per dose)[7]. Salvage therapy in such patient populations yielded a response rate of 18% and median response duration of 9 months.

These results emphasize the need to explore alternate salvage regimens for patients with relapsed/refractory AML. The development of novel and effective anti-AML agents and/or combinations is crucial to improving the outcome of AML.

2.2 B Newly Diagnosed AML in Older Patients

AML is primarily a disease of older people with a median age of diagnosis of 68 years. There are approximately 13,000 new cases of AML per year in the United States and 55% of these occur in individuals who are age 65 years and older. AML in older patients is phenotypically different from AML in younger patients. As compared to their younger counterparts, older patients with AML more frequently have an antecedent hematological disorder or unfavorable cytogenetics, and respond less well to chemotherapy

Elderly patients with AML have a poor prognosis, which is attributed to having disease that is inherently more resistant to current standard cytotoxic agents and/or relatively poor tolerance of these agents because of comorbidity and reduced tolerance of adverse effects. As a result, despite steady progress in the therapy of AML in younger patients, treatment for elderly patients with AML has not improved for decades. Among patients 70 years or older and those with adverse karyotypes the 4-8 week mortality with intensive chemotherapy is 30%-50%, and the median survival is 4-7 months. Therefore such patients derive little benefit from intensive chemotherapy, and some may in fact be harmed with intensive chemotherapy. Such high induction mortality rates and poor tolerance to chemotherapy have resulted in a reluctance to treat AML in older patients. A Surveillance Epidemiology and End Results database analysis has indicated that 64% of AML patients over age 65 receive no therapy for AML, other than supportive care. This untreated group has a dismal median survival of 1.7 months.

Hypomethylating agents are the most frequently used agents in the treatment of less fit, older patients with AML in the US and Europe. The DACO-016 study compared the efficacy and safety of decitabine (20 mg/m^2/day for 5 days every 4 weeks) versus treatment of choice (TC) (including LDAC 20 mg/m^2/day for 10 days every 4 weeks or best supportive care) in 485 patients with AML (median age 73 years) ineligible for cytotoxic chemotherapy (Kantarjian et al., JCO 2012; Thomas X et al., CLML 2014). The CR rate plus CRp was 17.8% with decitabine versus 7.8% with TC (odds ratio, 2.5; P = .001). The initial analysis showed a trend toward improved survival with decitabine (7.7 vs. 5.0 months; *P* = 0.108) that became significant (*P* = 0.037) with further follow-up. Azacitidine has shown benefit in elderly patients with AML in two large multicenter trials. In one trial patients with AML with 20–30% blasts were evaluated as a subset analysis of the phase III

AZA-001 trial (Fenaux et al., JCO 2010). The patients were randomly assigned to receive subcutaneous azacitidine or conventional care regimen CCR (best supportive care [BSC] only, low-dose cytarabine (LDAC), or intensive chemotherapy [IC]). The morphologic CR rate was 18% (10 of 55 patients) in the azacitidine group and 16% (nine of 58 patients) in the CCR group (*P* = .80). At a median follow-up of 20.1 months, the median OS for azacitidine-treated patients was 24.5 months compared with 16.0 months for CCR-treated patients (P = 0.005), and 2-year OS rates were 50% and 16%, respectively (P = 0.001). In another trial, 488 elderly patient's with AML with >30% blasts and age ≥65 years were randomized to receive either azacitidine (75 mg/m^2/day for 7 days every 4 weeks) or a CCR (standard induction chemotherapy, LDAC, or supportive care only) (Dombret et al., Blood 2015). Overall response (CR + CRi) rates were comparable in the azacitidine (27.8%) and CCR (25.1%) arms (*P* = .5384). Median OS was 10.4 months (1-year survival 47%) for patients receiving azacitidine compared to 6.5 months (1-year survival 34%) for patients receiving CCR (*P* = 0.0829). A prespecified analysis censoring patients who received AML treatment after discontinuing study drug showed median OS with azacitidine vs CCR was 12.1 months vs 6.9 months (stratified log-rank P = .0190). The dismal prognosis in elderly AML patients has resulted in increased efforts and clinical trials to improve the frontline therapy in elderly patients (≥ 60 years) with AML.

These results emphasize the need to explore alternate strategies to intensive chemotherapy for AML in older patients and/or those unable to tolerate intensive chemotherapy (based on predicted high eight-week mortality rates of 30% or more). The development of novel and effective anti-AML agents and/or combinations is crucial to improving the outcome of older patients (age ≥ 65) with AML. The clinical results with azacytidine and nivolumab in the salvage setting are encouraging (see clinical update of ongoing 5-azacytidine and nivolumab trial in section 2.3 C on page 4). Therefore, we would like to evaluate the efficacy and tolerability of the combination of 5-azacytidine with nivolumab in older patients with AML.

2.3 A Role of PD-1/PD-L1 and CTLA4 interactions in AML

It is well known that negative regulatory mechanisms within the solid tumor microenvironment inhibit antitumor T-cell function. Proteins produced by the tumor cells often inhibit the anti-tumor activity of the microenvironment by altering immune cells contained within the microenvironment and by recruitment of immune-suppressor cells to that microenvironment. One inhibitory mechanism is up-regulation of programmed death-ligand 1 (PD-L1) expressed on tumor or stromal cells, which binds to programmed death-1 (PD-1) on activated T cells[9-12]. PD-1/PD-L1 engagement results in diminished antitumor

response by producing a state of exhaustion for tumor-infiltrating cytotoxic T-lymphocytes (CTLs). PD-L1 expression is associated with poor prognosis in many cancers, including those of the lung, stomach, colon, breast, cervix, ovary, renal cell, and liver, as well as in adult T-cell leukemia, glioma, and melanoma[13-15]. Interaction between PD-L1 and PD-1 plays an important role in controlling immune responses and is involved in peripheral tolerance, autoimmunity, allergy, infection, and antitumor immunity[13, 16].

The PD-1/PD-L1 pathway plays a major role in immune evasion and cytotoxic T-cell exhaustion in hematologic malignancies including AML and MDS[17-19]. Garcia-Manero et al identified ≥ 2-fold expression of PD-L1 in 34% of patients with AML or MDS[19]. Chen et al found increased expression of PD-L1 at AML progression, which was an independent negative prognostic factor for French-American-British type M5 AML[20].

Similarly, Zhou et al have demonstrated that AML progression in a murine model is associated with elevated PD-1 expression on CTLs and increased T-regulatory cells at the tumor site[21]. Both these mechanism decrease CTL activity at the tumor site. Elevated PD-1 expression independently dampens the anti-leukemic effect of CTLs. T-regulatory cells (Tregs) further suppress CTL activity and this suppression depends on PD-1 expression by Tregs and PD-L1 expression by antigen-presenting cells. Anti-PD-L1 monoclonal antibody treatment increased the proliferation and function of CTLs at tumor sites by reducing the interaction between PD1 and PDL1 resulting in decreased Treg-mediated suppression of CTLs. The enhanced CTL activity resulted in reduced AML tumor burden, and resulted in long-term murine survivors.

CTLA4: The role of CTLA4 in AML is not as well defined in AML. However, recent data suggest the CTLA-4 pathway may indeed play a role in AML and MDS, especially postSCT. Matthews et al evaluated anti-CTLA4 agent ipilimumab in a phase I/IB study in patients with relapsed AML post-allogeneic stem cell transplant (Matthews D et al., NEJM 2016; 375:143-153). Among 28 patients with post-SCT relapse 13 had relapsed AML (including medullary and/or extramedullary relapse). The patients had a median of 3 prior therapies for AML. A complete response was obtained in 5/13 patients. The therapy was generally well tolerated, with 2/23 cases experience any grade immune AE generally well controlled by early recognition and initiation of steroids. In a study at MDACC in patients with MDS who have progressed post-hypomethylator therapy we evaluated single-agent nivolumab as well as single agent (Garcia-Manero G, Daver N et al., American Society of Hematology, 2016, abstract # 344). Nivolumab as a single-agent did not induce a response in 15 patients with post-HMA MDS and the nivolumab alone cohort was closed to new patient entry per futility stopping rules. In contrast, ipilimumab as a single agent in patients with postHMA MDS produced a CR in 2 of 9 patients treated indicating modest activity. The ipilimumab cohort is being expanded with plans to proceed to a nivolumab+ipilimumab cohort that is built into the MDS protocol and the one expected to have best responses in MDS and possibly AML, similar to outcomes noted in solid tumors.

2.3 B Defining PD1, CTLA4 and Immune checkpoint expression in AML (MDACC experience) Between March, 2015 and October 2015 we performed 17-color multi-parameter flow- cytometry (MFC) on 15 untreated AML and 25 relapsed AML to assess expression of costim ligands (4-1BBL, B7-1, B7-2, ICOSL, PDL-1, PDL-2, OX40L) on

leukemic blasts and costim receptors (4-1BB, CTLA-4, ICOS, PD-1, OX40, GITR, LAG-3, TIM-3) on Tcell subsets: CD4 T effector cells [Teff]: $CD3^{+}CD4^{+}CD127^{lo/+}Foxp3^{-}$, CD4 T regulatory cells [Treg]: $CD3^{+}CD4^{+}CD127^{-}Foxp3^{+}$, and CD8 T cells: $CD3^{+}CD8^{+}$. Four healthy human BMs were used as control. Expression is denoted by percentage of specific T-cell subset or gated AML blasts positive for the marker indicated. PB mononuclear cells (PBMCs) and blasts were evaluated at the same time-point. OX40+ Teffs and OX40+ CD8 cells were higher in untreated AML BM as compared to healthy donor BM (median [med]: 7.2% versus [vs] 0.26%; P=0.0005 and med: 2.03% vs 0.06%; P=0.07, respectively). PD1+ Tregs and OX40+ Tregs were also higher in untreated AML BM as compared to healthy donor BM (med: 19.7% vs 7.5%; P=0.03 and med: 10.3% vs 0.5%; P=0.02, respectively). PD1+ Teffs, OX40+ Teffs, and ICOS+ Teffs were higher in relapsed AML BM as compared to healthy donor BM (med: 17.7% vs 6.7%; P= 0.047, med: 12% vs 0.27%, P=0.002, and med: 13.3% vs 1.1%; P=0.07, respectively). OX40+ CD8 cells, ICOS+ CD8 cells, TIM3+ CD8 cells (med: 5.0% vs 0.07%; P=0.04, med: 18.5% vs 2.6%; P=0.09, and med: 2.7% vs 0.7%; P=0.01, respectively) and OX40+ Tregs (med: 15.5% vs 0.5%; P<0.0001) were also higher in relapsed AML BM as compared to healthy donor BM. GITR+ Teffs, PD1+ CD 8 cells, and LAG3+ Tregs were higher in relapsed AML BM as compared to new AML BM (med: 10.9% vs 1.7%; P=0.08, med: 36.2% vs 21.3%; P=0.03, and med: 46.7% vs 16.9%; P=0.07, respectively). No other noteworthy differences were noted for costim receptor expression. CTLA4 expression on CD3+ T-cells as well as Teff, Treg, and CD8+ T-cells was evaluated and was found to be expressed on <10% of each respective population and was not significantly increased as compared to CTLA4 expression on corresponding T-cell populations in healthy donors (Daver N, Sharma P et al., American Society of Hematology 2016, abstract # 2900). There were no other noteworthy differences in ligand expression patterns between relapsed AML and new AML. There was significant variability in BM expression of costim receptors and ligands between individual pts. The expression of costim receptors and ligands differed significantly between BM and PB from the same time-point in the same pt. A larger sample size is needed to confirm these data and find additional associations and this is currently underway at our institution.
Thus, it appears clinically targetable checkpoint receptors including PD1 appear to be overexpressed in the BM of patients with AML. Relapsed AML had higher expression of costim receptors. These data have been submitted (Daver N, Sharma P et al. AACR 2016) and further support the evaluation of anti-PD1 based therapies in AML.

2.3 C 5-azacytidne and nivolumab clinical trial update (11/29/2016):

We have thus far treated 70 patients on the vidaza + nivolumab trial for relapsed/refractory AML as of Dec 30 2016 (Daver N, Sharma P et al., American Society of Hematology, 2016 abstract #763). The RP2D of 5-azacytidine and nivolumab was established as 5-azacytidine 75mg/m^2 days 1-7 and nivolumab 3.0 mg/kg on days 1 and 14 of each cycle in the ongoing clinical trial. 0/6 patients experienced a DLT at this dose during the safety-run-in phase. All subsequent 47 patients have received this RP2D dose of 5-azacytidine and nivolumab.
51 patients have completed at least 3 cycles and are evaluable for response and toxicity. 51 pts with a median age of 69 years (range, 45 – 90), secondary AML (55%), poor risk cytogenetics (47%), median number of prior regimens 2 (range, 1-7) have been enrolled. All 51 pts had baseline next generation sequencing and most frequently detected mutations included *DNMT3A* (n=12), TP53 (n=11), TET2 (N=9), *ASXL1* (n=7), *and RAS* (n=7). 35 pts

are evaluable for response: 6 (18%) achieved complete remission (CR)/ complete remission with insufficient recovery of counts (CRi) (3 CR, 3 CRi), 5 (15%) had hematologic improvement (HI), 9 (26%) had ≥50% BM blast reduction, 3 pts (9%) had stable disease > 6 months, and 12 (34%) had progression. 16 pts are early for response assessment (<3 courses). The median number of courses to CR/CRi/HI was 3 (1 – 9). The CR/CRi/HI (n=11) have been durable with no relapses among pts who achieved CR/CRi/HI [CR duration; not reached (NR)]. The 4- and 8-week mortality were 0 and 6%, respectively. The median overall survival for the 35 evaluable pts was 9.3 months (range, 1.8 – 14.3) (Fig 1) and this compares favorably to historical survival with AZA-based salvage protocols in similar pts treated at MDACC.
Grade 3/4 and Grade 2 immune mediated toxicities were observed in 7 (14%) and 6 (12%) pts, respectively. These included 8 episodes of pneumonitis, 2 nephritis, 2 transaminitis, and 1 skin rash. 1 pt died from grade 4 pneumonitis/epiglottitis. In the remaining 12 cases the toxicities responded rapidly to steroids and all 12 pts were successfully rechallenged with nivolumab. Time to onset of toxicities ranged from 4 days to 3.5 months. Four pts were postSCT and one had a grade 3 flare of GVHD of the skin and gut.
Multicolor flow-cytometry studies and Mass-cytometry (CyTOF) studies are conducted by the Immunotherapy Platform on baseline and on-treatment BM aspirate (end of cycle 1, 2, 4, 8) and peripheral blood to assess the T-cell repertoire and expression of co-stimulatory receptors and ligands on T cell subsets and leukemic blasts respectively. Baseline and end of cycle 1 BM was evaluated on 25 of the 35 evaluable patients, including 6 responders (CR/CRi/HI) and 19 non-responders. Pts who achieved a CR/CRi/HI had a baseline higher live total CD3 (P=0.10), $CD8^+$ T-cells (P=0.02), and lower live $CD4^+Foxp3^+PD1^+$ T-regulatory (T-reg) cells (P=0.01) infiltrate in the BM (Figure 2). The ratio of $PD1^+CD8^+$ T-effector cells to $PD1^+CD4^+Foxp3^+$ T-reg cells was significantly higher in the CR/CRi/HI pts vs non-responders (208.4 vs 8.91, P<0.05) in baseline BM. On further evaluation of post cycle 1 and post cycle 2 BM aspirates (unpublished – to be presented at ASH 2016) the responders demonstrate a progressive increase in the live CD3+ and CD8+ BM infiltrate and an increase in CD8+ ICOS+ cells. On the other hand non-responders did not have an increase in the live CD3+ or T-cell subsets. However, CTLA4 and Tim3 were up-regulated on CD8+, T-eff and to a lesser extent on T-regs post-cycle 2 suggesting up-regulation of these exhaustion markers as a potential mechanism of resistance.
We have treated 5 patients who are older than 60 years and not candidates for induction chemotherapy with vidaza in combinaiton with nivolumab as frontline therapy. The patients have not completed 3 cycles of therapy and are early for efficacy analysis. The regimen has been well tolerated with no patients requiring drug interruptions or discontinuation of therapy due to toxicities. Immune mediated toxicities have been noted in 3 patients including 2 episodes of Grade 2 pneumonitis and 1 episode of Grade2 nephritis. All 3 cases of immune mediated toxicity responded rapidly to steroid therapy and were resolved within 7 days.
Our conclusion based on these data were that full dose AZA and nivolumab are tolerable and produce an encouraging response rate with durable responses in relapsed AML with poor risk features. Immune mediated toxicities occur and may be adequately managed with early recognition and systemic steroids. On prelim analysis responders had higher baseline BM $CD8^+$ T-effectors, lower T-regs and higher $PD1^+CD8$/ $PD1^+CD4^+Foxp3^+$. Up-regulation of inhibitory checkpoints CTL4 and Tim3 may be a mechanism of resistance and dual blockade of PD1 and CTLA4 may abrogate such resistance increasing the response rate.

2.4 Nivolumab (BMS-936558, MDX1106):

2.4.1 Mechanism of Action

Immune activation is tightly regulated by co-stimulatory (e.g. CD28 and ICOS) and co-inhibitory (e.g. CTLA-4 and PD-1) receptors expressed on T cells. Agonistic antibodies against co-stimulatory T cell receptors and blocking antibodies against co-inhibitory T cell surface receptors have both been shown to potentiate T cell activation for tumor cell killing.

PD-1 is mainly expressed by activated CD4+ and CD8+ T cells, as well as antigen presenting cells (APCs). It has two ligands, PD-L1 and PD-L2, with distinct expression profiles.[16] PD-L1 is expressed not only on APCs, but also on non-hematopoietic cells, including tumor cells. Expression of PD-L2 is largely restricted to APCs including macrophages and myeloid dendritic cells, as well as mast cells. The role of PD-1 as a negative regulator of T cells was best demonstrated by the finding that PD-1 deficient mice developed significant autoimmunity with high titers of autoantibodies.[22, 23] Subsequently, blocking antibodies against PD-1 were shown to activate immune responses that resulted in reduction of tumor metastasis and tumor growth in a number of experimental tumor models.[24, 25] Consistent with the immune inhibitory role of PD-1/PD-L1/2 signaling, forced expression of PD-L1 in murine tumor cell lines allowed increased tumor growth in vivo, which was otherwise kept in check by T cells. The inciting effect of PD-L1 on tumor growth was reversed by blocking PD-L1 with anti-PD-L1

antibodies.[26]

Nivolumab (BMS-936558) is a fully human, IgG4 (kappa) isotype, monoclonal antibody that binds PD-1. Blockade of the PD-1 pathway by nivolumab was studied using the mixed lymphocyte reaction (MLR). PD-1 blockade resulted in a reproducible enhancement of both proliferation and IFN-γ release in the MLR. The effect of nivolumab on antigen-specific recall response was investigated using a CMV-restimulation assay with human peripheral blood mononuclear cells (PBMCs), and was evaluated by ELISA. These data indicated that nivolumab, versus an isotype-matched control antibody, augmented IFN-γ secretion from CMV-specific memory T cells in a dose-dependent manner. PD-1 blockade by nivolumab is therefore considered a promising immunotherapeutic strategy.

2.4.2 Summary of Safety Results from Nivolumab Program

For a complete review of clinical information, please refer to the nivolumab Investigator Brochure.

2.4.2.1 Summary of Safety

The monitoring of subject safety during and after a clinical study with nivolumab, including any special monitoring precautions, tests, or observations, and the proper means of recording and reporting adverse safety information [including adverse events (AEs) and abnormal laboratory values] will follow the procedures

outlined in the specific study protocol.

The overall safety experience with nivolumab is based on experience in approximately 1500 subjects as either a monotherapy or in combination with other therapeutics. In general for monotherapy, the safety profile is similar across tumor types. The one exception is pulmonary inflammation AEs which may be numerically greater in subjects with non-small cell lung cancer (NSCLC) possibly because in some cases it can be difficult to distinguish between nivolumab-related and unrelated causes of pulmonary symptoms and radiographic changes. The most frequently reported treatment-related AE is fatigue, which is almost always low grade.

The safety profile is generally consistent across completed and ongoing clinical trials with no maximum tolerated dose (MTD) reached at any dose tested up to 10 mg/kg. There was no pattern in the incidence, severity, or causality of AEs to nivolumab dose level. Most related AEs are thought to be due to the effects of inflammatory cells on specific tissues. Most AEs were low-grade (Grade 1 to Grade 2) with relatively few related high-grade (Grade 3 to Grade 4) AEs. Most high-grade events were manageable with use of corticosteroids or hormone replacement therapy (endocrinopathies).

2.4.2.2 Clinical Safety in Advanced Malignancies (Nivolumab Monotherapy)

A total of 306 subjects with treatment-refractory malignancies have been treated

in an ongoing, Phase 1 multidose study (MDX1106-03, CA209003). This is an ongoing phase I dose-escalation study of nivolumab monotherapy in patients with advanced cancers; 1, 3, or 10 mg/kg nivolumab and 0.1 and 0.3 mg/kg (included as part of Amendment 4) administered by IV Q2W; treatment up to 2 years. Results were published by Topalian et al. (NEJM 2012).[27] The baseline disease diagnosis by treatment for MDX1106-03 is provided in Table 1. A review of the safety data by tumor type (RCC, NSCLC, mCRPC, CRC, and melanoma) did not show any clinically meaningful differences in the proportion of subjects with AEs noted across tumor type.

Table 1: Baseline Disease Diagnosis by Treatment - MDX1106-03

	No. of Subjects					
**Nivolumab (mg/kg)**	**0.1 mg/kg**	**0.3 mg/kg**	**1 mg/kg**	**3 mg/kg**	**10 mg/kg**	**TOTAL**
**MDX1106-03, Total N**	**17**	**18**	**86**	**54**	**131**	**306**
NSCLC	0	0	33	37	58	128
Melanoma	17	18	35	17	20	107

RCC	0	0	18	0	16	34
mCRPC	0	0	0	0	17	17
CRC	0	0	0	0	19	19

Abbreviations: CRC: colorectal adenocarcinoma; mCRPC: metastatic castration-resistant prostate cancer; NSCLC: non-small cell lung cancer; RCC: renal cell carcinoma;

Source: Preliminary data, CA209003. Clinical data cut-off date 18 Mar-2013.

2.4.2.3 Adverse Events

There was no pattern in the incidence, severity, or causality of AEs related to the dose of nivolumab, between 1 and 10 mg/kg, in MDX1106-03. Of the 306 treated subjects in MDX1106-03, 303 (99.0%) subjects have at least 1 reported AE regardless of causality (Table 2). The most frequently reported AEs were fatigue (54.9%), decreased appetite (35.0%), diarrhea (34.3%), nausea (30.1%), and cough (29.4%). Treatment-related AEs were reported in 230 (75.2%) of the 306 subjects. The most frequently reported treatment-related AEs were fatigue (28.1%), rash (14.7%), diarrhea (13.4%), and pruritus (10.5%). Most treatment-related AEs were low grade. **Treatment-related Grade 3-4 AEs were reported in 52 (17.0%) of subjects.** The most frequently reported treatment-related high grade AE was fatigue (6.5%).

Table 2.: Summary of Adverse Events Reported in ≥15% of All Treated Subjects- MDX1106-03

**Preferred Term**	**No. of Subjects (%)**<br>**AEs regardless of causality**<br>**Any Grade N=306**	**Grade 3-4 N=306**	**Treatment-related AEs**<br>**Any Grade N=306**	**Grade 3-4 N=306**
**Any AE**	**303 (99)**	**127 (42)**	**230 (75)**	**52 (17)**
Fatigue	168 (55)	20 (7)	86 (28)	7 (2)
Decreased appetite	107 (35)	3 (1)	28 (9)	1(0.3)
Diarrhea	105 (34)	3 (1)	41 (13)	3 (1)
Nausea	92 (30)	9 (3)	27 (9)	2 (1)
Cough	90 (29)	4 (1)	11 (4)	1 (0.3)
Dyspnea	80 (26)	27 (9)	11 (4)	0
Constipation	78 (26)	2 (1)	5 (2)	0
Rash	74 (24)	0	45 (15)	0
Vomiting	70 (23)	7 (2)	10 (3)	1 (0.3)
Back pain	68 (22)	7 (2)	3 (1)	1 (0.3)

Arthralgia	63 (21)	4 (1)	15 (5)	0
Pyrexia	61 (20)	1 (0.3)	17 (6)	0
Headache	59 (19)	1 (0.3)	8 (3)	0
Edema peripheral	59 (19)	1 (0.3)	3 (1)	0
Dizziness	56 (18)	1 (0.3)	10 (3)	0
Pruritus	56 (18)	1 (0.3)	32 (11)	1 (0.3)
Weight decreased	48 (16)	1 (0.3)	11 (4)	0
Malignant neoplasm progression	48 (16)	4 (1)	1 (0.3)	1 (

Source: Preliminary data, MDX1106-03. Clinical data cut-off date: 18-Mar-2013

2.4.2.4 Select Adverse Events

Select AE categories (events with a potential inflammatory mechanism requiring more frequent monitoring and/or unique intervention such as immunosuppressant's and/or endocrine replacement therapy) include: GI AEs, pulmonary AEs, renal AEs, hepatic AEs, skin AEs, and endocrinopathies. In addition, Select AEs include a category for infusion reactions. Each category is composed of a discrete set of preferred terms, including those of greatest clinical relevance. These Select AEs are considered events of interest based on the mechanism of action and were previously referred to as immune-related AEs or immune-mediated AEs.

The frequencies of these events are summarized in investigator brochure (Appendix E). The 10-mg/kg cohort had numerically greater frequency of high-grade select AEs including the subcategories of endocrinopathies, GI, pulmonary, and infusion reactions.

2.4.2.5 Adverse Events Leading to Discontinuation

At least 1 treatment-related AE leading to discontinuation was reported in 32 (10.5%) of the 306 treated subjects. Grade 3-4 treatment-related events were reported in 14 (4.6%) subjects. The frequency of treatment-related AEs leading to discontinuation was not associated with the dose of nivolumab. Pneumonitis was the most common treatment-related AE leading to discontinuation (8 subjects, 2.6%); pneumonitis reported in 3 (1.0%) subjects was Grade 3-4. Treatment-related AEs reported in at least 2 subjects included pneumonitis (8 subjects, 2.6%), colitis (3 subjects, 1.0%) and myalgia, hepatitis, hypersensitivity, and infusion-related reactions (each reported in 2 subjects, 0.7%). One event of Grade 5 sepsis was reported for 1 subject, a 62-year-old male treated with 1 mg/kg nivolumab.

2.4.2.6 Deaths

As of 18-Mar-2013, 195 deaths have been reported in MDX1106-03 during the course of the study or within 30 days of last dose of study drug. The majority of the deaths were considered secondary to disease progression and malignant disease.

Three subjects in MDX1106-03 died after developing pneumonitis. A 62-year-old male (MDX1106-03-1-699) with NSCLC (adenocarcinoma) in the 1 mg/kg treatment group and a 59-year old male (MDX1106-03-1-3582) with CRC in the 10 mg/kg treatment group both died due to Grade 5 sepsis after having developed Grade 4 pneumonitis. Sepsis and pneumonitis were considered related to study drug by the investigator in both of these cases. In addition, a 40-year- old female (MDX1106-03-5-710) with NSCLC (adenocarcinoma) in the 1 mg/kg treatment group died due to respiratory failure after having developed Grade 4 pneumonitis and tumor progression. In this case, respiratory failure and pneumonitis were considered related to study drug by the investigator.

Additional deaths reported as due to “other” in MDX1106-03 included:

- MDX1106-03-2-3436 (10 mg/kg): Ischemic cardiomyopathy
- MDX1106-03-4-3484 (10 mg/kg): Death due to abdominal pain caused by superior mesenteric vein thrombosis
- MDX1106-03-3-674 (1 mg/kg): Progressive lung cancer

2.4.3 Summary of Efficacy (Nivolumab monotherapy)

The clinical activity data presented below are from MDX1106-03 (nivolumab monotherapy).

Table 3: Objective Response Rate and Progression Free Survival 24 Weeks Rate in Melanoma Subjects - MDX1106-03

Dose (mg/kg)	N	ORR	PFSR at 24 weeks (%)
**All Melanoma**	**107**	**33 (31)**	**4**
0.1	17	6 (35)	3
0.3	18	5 (28)	3
1.0	35	11 (31)	5
3.0	17	7 (41)	5
10.0	20	4 (20)	3

Abbreviations: ORR: objective response rate, PFSR: progression-free survival rate

Source: Preliminary data, MDX1106-03. Clinical cut-off date: 18-Mar-201

Table 4: Objective Response Rate per RECIST 1.0 and Progression Free Survival 24 Weeks Rate by Histology in Non-small Cell Lung Cancer Subjects - MDX1106-03

1.0	15	S	0	36
	18	NSQ	1 (6)	19
3.0	18	S	4 (22)	45
	19	NSQ	5 (26)	42
10.0	21	S	5 (24)	45
	37	NSQ	7 (19)	25

Abbreviations: NA: not applicable, NSCLC: non-small cell lung cancer; NSQ: non-squamous, ORR: objective response rate; PFSR: progression free survival rate, SQ: squamous

Source: Preliminary data, MDX1106-03. Clinical cut-off date 18- Mar-2013.

2.4.3.1 Ipilimumab:

Ipilimumab (BMS-734016, MDX010, MDX-CTLA4) is a fully human monoclonal immunoglobulin (Ig) G1κ specific for human cytotoxic T lymphocyte antigen 4 (CTLA-4, CD152), which is expressed on a subset of activated T cells. CTLA-4 is a negative regulator of T-cell activation. Ipilimumab binds to CTLA-4 and inhibits its interaction with ligands on antigen-presenting cells (APCs). The proposed mechanism of action for ipilimumab’s effects in subjects with melanoma is indirect, possibly through T-cell potentiation and mediation of antitumor immune responses.

Yervoy™ (ipilimumab) has been approved for use in over 40 countries including the United States (US, Mar-2011), the European Union (Jul-2011), and Australia (Jul-2011. Bristol-Myers Squibb (BMS) and Medarex, Inc. (MDX, acquired by BMS in Sep-2009) have co-sponsored an extensive clinical development program for ipilimumab, encompassing more than 13,800 subjects in several cancer types in completed and ongoing studies, as well as a compassionate use program. The focus of the clinical program is in melanoma, prostate cancer, and lung cancer, with advanced melanoma being the most comprehensively studied indication. Ipilimumab is being investigated both as monotherapy and in combination with other modalities such as chemotherapy, radiation therapy, and other immunotherapies. Phase 3 programs are ongoing in melanoma, prostate cancer, and lung cancer. In melanoma, 2 completed Phase 3 studies (MDX010-20 and CA184024) have demonstrated a clinically meaningful and statistically significant survival benefit in pretreated advanced melanoma and previously untreated advanced melanoma, respectively ®).

The safety profile of ipilimumab is generally consistent across these trials with a) the

majority AEs being inflammatory in nature consistent with the proposed mechanism of action of ipilimumab, b) the same types of such immune-mediated events in the gastrointestinal (GI) tract, skin, liver, and endocrine system being reported, and c) most of these events being manageable with immune suppressive therapies. In melanoma, 2 BMS-sponsored Phase 3 studies are ongoing in subjects with high-risk Stage III melanoma (CA184029, with adjuvant immunotherapy) and pretreated and treatment-naïve advanced melanoma (CA184169, 3 mg/kg versus 10 mg/kg ipilimumab). The completed Phase 3 study CA184043 evaluated ipilimumab in subjects with metastatic castration-resistant prostate cancer (mCRPC) who had progressed during or following docetaxel. Eligible subjects were randomized to a single dose of bone-directed radiotherapy (RT), followed by either ipilimumab 10 mg/kg or placebo (799 randomized: 399 ipilimumab, 400 placebo). This study did not meet its primary endpoint of overall survival (OS). The hazard ratio (HR) of 0.85 (95% CI: 0.72 to 1.00) for survival favored ipilimumab but did not reach statistical significance with a P-value of 0.053. Planned sensitivity analyses favored ipilimumab, where the greatest benefit appeared to be in subgroups defined by good prognostic features and low burden of disease. Additional evidence of ipilimumab activity observed in the study included a reduced risk of disease progression relative to placebo (HR = 0.70) and superior clinical outcomes compared to placebo in tumor regression and declines in prostate specific antigen (PSA). The safety profile in this study was consistent with the previously defined adverse event (AE) profile at the same dose. A second Phase 3 study CA184095 evaluating ipilimumab 10 mg/kg versus placebo in men with asymptomatic or minimally symptomatic, chemotherapy-naïve mCRPC with no visceral metastases is ongoing. In addition, a completed large Phase 2 study (CA184041) has investigated the addition of ipilimumab to carboplatin and paclitaxel using 2 different schedules (concurrent and phased) in subjects with nonsmall cell lung cancer (NSCLC) or small cell lung cancer (SCLC, a secondary endpoint). The phased, but not the concurrent schedule, demonstrated activity in both NSCLC and SCLC, including significant improvement of immune-related progression-free survival (irPFS) and a numeric trend for OS improvement (not significant). The efficacy and safety of ipilimumab in a phased schedule with carboplatin/paclitaxel is also being investigated in an ongoing Phase 3 study in subjects with advanced squamous NSCLC (CA184104). The efficacy and safety of

ipilimumab in a phased schedule with etoposide/platinum in subjects with extensive stage disease SCLC is being investigated in an ongoing Phase 3 study (CA184156). The unique immune-based mechanism of action is reflected in the clinical patterns of anti-cancer activity in some patients. Ipilimumab impacts tumor cells indirectly, and measurable clinical effects emerge after the immunological effects. Tumor infiltration with lymphocytes and the associated inflammation (documented by biopsy in some subjects) is likely the cornerstone of the effect of ipilimumab and can manifest in various patterns of clinical activity leading to tumor control. In some cases, inflammation may not be noted by radiological examination and objective response is observed with the first tumor assessment in a manner seen in patients receiving other types of anti-cancer treatments. In other cases, response may be preceded by an apparent increase in initial tumor volume and/or the appearance of new lesions, which may be mistaken for tumor progression on radiological evaluations. Therefore, in patients who are not experiencing rapid clinical deterioration, confirmation of progression is recommended, at the investigator's discretion, to better understand the prognosis as well as to avoid unnecessarily initiating potentially toxic alternative therapies in subjects who might be benefiting from treatment. Immune-related response criteria were developed based on these observations to systematically categorize novel patterns of clinical activity and are currently being prospectively evaluated in clinical studies. In metastatic diseases, stabilization is more common than response, and in some instances is associated with a slow, steady decline in tumor burden over many months, sometimes improving to partial and/or complete responses. Thus, the immune-based mechanism of action of ipilimumab results in durable disease control, sometimes with novel patterns of response, which contribute to its unique improvement in OS.

The unique immune-based mechanism of action is also reflected in the safety profile. The most common treatment-related AEs are inflammatory in nature, consistent with the mechanism of action of the drug and generally medically manageable with topical and/or systemic immunosuppressants. Such immunological safety events are described as irAEs or immune-mediated adverse reactions (imARs). The irAEs are described as AEs of unknown etiology, which were consistent with an immune phenomenon and were considered causally related to drug exposure by investigators.

The irAEs primarily involve the GI tract and skin. Immune-related AEs in the liver were also observed, particularly in subjects receiving 10 mg/kg. Endocrinopathy and neuropathy were important irAEs observed less frequently. The imARs were adjudicated in a blinded fashion based on sponsor-physician data review to exclude noninflammatory etiologies, such as infection or tumor progression, and to consider available evidence of inflammation, such as tumor biopsies or responsiveness to steroids, in an effort to determine whether specific AEs or abnormal hepatic laboratory values were likely to be immune mediated and associated with ipilimumab treatment. The early diagnosis of inflammatory events is important to initiate therapy and minimize complications. Inflammatory events are generally manageable using symptomatic or immuno-suppressive therapy as recommended through detailed diagnosis and management guidelines. The management guidelines for general irAEs and ipilimumab-related GI toxicities, hepatitis, endocrinopathy, and neuropathy are described in appropriate section. A program-wide independent Data Monitoring Committee (DMC) reviews data from the ipilimumab studies, allowing for an ongoing safety and benefit/risk assessment in subjects receiving ipilimumab. The DMC charter includes explicit stopping rules for some studies, allowing the DMC to recommend discontinuing further treatment across the ipilimumab program, if necessary.

In summary, ipilimumab offers clinically meaningful and statistically significant survival benefit to patients with pretreated advanced melanoma (as 3 mg/kg monotherapy compared to the melanoma peptide vaccine gp100) and previously untreated advanced melanoma (at 10 mg/kg in combination with dacarbazine [DTIC] compared to DTIC alone), and evidence of clinical activity in randomized studies in other tumor types. These findings, together with evidence of a safety profile that is manageable with careful monitoring and appropriate intervention for treatment of immune-related toxicities, suggest an acceptable benefit to risk ratio.

For further details please refer to the most recent version of the Ipilimumab Investigator Brochure attached as an Appendix.

2.5 5-azacytidine (Vidaza):

5-azacytidine, an analog of the pyrimidine nucleoside cytidine, has effects on cell differentiation, gene expression, and deoxyribonucleic acid (DNA) synthesis and metabolism. Since the early 1970s, 5-azacytidine has been investigated primarily in the US for the treatment of acute leukemia. Clinical studies have focused mainly on patients with disease refractory to conventional chemotherapy. Results of these investigations demonstrated activity of 5-azacytidine in the treatment of AML. Clinical studies subsequently evaluated the effects of 5-azacytidine in a variety of other malignant and hematologic disorders, including solid tumors, hemoglobinopathies (eg, thalassemia and sickle cell anemia), and MDS. In 1984, the Cancer and Leukemia Group B (CALGB) began a series of clinical studies with 5-azacytidine in patients with MDS. These studies, in addition to other supportive data, led to the approval of Vidaza® (5-azacytidine) in May 2004 for the treatment of MDS.

5-azacytidine inhibits the methylation of newly synthesized DNA by inhibiting DNA methyltransferase (DNMT).[28-30] Increased methylation of DNA (hypermethylation) may result in the silencing of critical genes responsible for cell growth control and differentiation. Hypermethylation of CpG islands spanning the promoter regions of tumor suppressor genes is commonly associated with cancers.[31] It is now widely recognized that hypermethylation of DNA is frequently associated with myelodysplastic syndromes and other cancers,[32-34] such as renal,[35] melanoma,[36] breast,[37] colorectal,[38] non-small cell lung[39] and hematologic malignancies.[40] 5-azacytidine is believed to exert its antineoplastic effects through hypomethylation and cytotoxicity on abnormal hematopoietic cells in the bone marrow.[41-45] Hypomethylation may restore normal function to genes that are critical for differentiation and proliferation.[31, 46, 47] The cytotoxic effects of 5-azacytidine cause the death of rapidly dividing cells, including cancer cells that are no longer responsive to normal growth control mechanisms.[41, 48-50]

The cytotoxicity of 5-azacytidine is proportional to dose and exposure time.[41, 42] Although the mechanisms of cytotoxicity are complex and multifaceted, there is general agreement that incorporation of 5-azacytidine into DNA and ribonucleic acid (RNA), and inhibition of protein synthesis, are critically important.[51] Cytotoxicity is greatest in cells that are proliferating (S phase) and metabolically active.[41] Cytotoxic effects may also be mediated through induction of the DNA damage response pathways.[50] Nonproliferating cells are relatively insensitive to 5-azacytidine.[41]

Toxicology studies have been conducted in mice, rats, dogs, and Rhesus monkeys.[52] Most of the studies were performed during the 1970s and early 1980s according to existing guidelines and standards in place during that period. The preclinical studies identified the bone marrow, liver, kidneys, and lymphoid tissues (spleen, lymph nodes, and thymus) as the main target organs of toxicity for 5- azacytidine.[52] In single-dose studies, the lethal dose of 5-azacytidine

after intravenous (IV) administration in mice, rats, and dogs was approximately 250 mg/m^2. Repeated daily dosing appears to increase the toxicity of 5- azacytidine.[52] The genotoxicity of 5-azacytidine is consistent with that of other nucleoside analogs that interact with nucleic acids.[52] Likewise, similar to other
agents with cytostatic properties, 5-azacytidine was embryotoxic and reduced the reproductive performance in mice and rats.[52]

Limited 5-azacytidine pharmacokinetic data are currently available. Based on human plasma concentrations of total radioactivity (which represents parent drug plus circulating metabolites), 5-azacytidine is rapidly absorbed when given subcutaneously (SC), with maximum plasma concentrations found 0.5 to 2 hours after dosing.[52] 5-azacytidine and/or its by-products are then rapidly cleared by the kidneys. The half-lives and percent radioactivity recovered in urine are similar following IV and SC routes of administration. The effects of renal or hepatic impairment, gender, age, or race on the pharmacokinetics of 5-azacytidine have not been studied.[52] A single dose (75 mg/m^2) SC versus IV crossover study in 6 MDS subjects[53] revealed an approximate bioavailability of 89% for the SC dose (range 52% to 128%) with mean half-lives of 0.69 hour and 0.36 hour after SC and IV administration, respectively. These results demonstrated that 5-azacytidine is rapidly and nearly completely absorbed after SC administration and that elimination is also rapid. The apparent SC clearance (167 L/h or 2791 mL/min) and systemic IV clearance (147 L/h) of 5-azacytidine exceeded the glomerular filtration rate (approximately 125 mL/min) and total renal blood flow (1200 mL/min) in healthy subjects. This indicates that non-renal elimination (eg, metabolism, hydrolysis, and/or degradation) plays a role in the elimination of parent 5-azacytidine. In addition, 5-azacytidine 75 mg/m^2 was generally well-tolerated after single SC injection or IV infusion over 10 minutes.[53]

A number of studies have looked at different parenteral doses and schedules of 5-azacytidine, finding maximum tolerated doses of up to 500 mg/m^2 when administered weekly.[54]

During the two decades between the start of the CALGB studies and the approval of 5-azacytidine, a new understanding of MDS has developed, such as the World Health Organization (WHO) diagnostic criteria, the International Prognostic Scoring System (IPSS), and the International Working Group (IWG) response criteria. Silverman et al. reevaluated the combined data (N = 309) from 3 of the CALGB studies using the WHO classification system for MDS and AML and the IWG response criteria.[55] Using the IWG response criteria in MDS patients, response rates were between 40% and 70% in 5-azacytidine treated patients. Ten to 17% of patients achieved a complete remission; partial remission was rare; and 23% to 36% of patients had a hematologic improvement. In patients with AML (N = 103), 35% to 48% had hematologic improvement or better responses. The median survival time for 27 patients assigned to 5-azacytidine was 19.3 months compared with 12.9 months for the 25 patients assigned to observation.[55]

A randomized international Phase III trial (Study 5-azacytidine PH GL 2003 CL 1) for higher-risk MDS patients, classified by FAB as RAEB, RAEB-T, or CMML with 0-29% marrow blasts, with an IPSS of Intermediate -2 or High by central pathology/cytogenetic review was recently reported.[56] Patients were randomized to 5-azacytidine (75 mg/m^2/day x 7days in 28 day cycles) or conventional care regimens (CCR), where CCR was pre-selected by the Investigator as best supportive care (transfusions, antibiotics, and G-CSF for neutropenic infection), low-dose cytarabine (20 mg/m^2/day x 14 days in 28 day cycles); or standard chemotherapy (conventional induction/consolidation). Patients were stratified by FAB/IPSS and randomized 1:1 to 5-azacytidine or CCR. This trial did not allow erythropoietin. Three hundred fifty eight patients (70% male) were randomized at 79 centers to 5-azacytidine (N=179) or CCR (N=179): best supportive care only (N=105, 59%), low-dose cytarabine (N=49, 27%), or standard chemotherapy (N=25, 14%). Median age was 69 years (range 38-88 years). The 5-azacytidine and CCR groups were comparable for baseline patient characteristics. At baseline, 95% of patients were higher risk: RAEB (58%), RAEB-T/WHO AML (34%), CMML (3%), and other (5%). By IPSS, 87% were higher risk: Intermediate -2 (40%), High (47%), and 13% Indeterminate/other. 5-azacytidine was administered for a median of 9 cycles; low-dose cytarabine for 4 cycles. Median follow-up for the survival analysis was 21.1 months. 5-azacytidine demonstrated statistically superior overall survival compared to CCR, with a median overall survival of 24.4 months vs. 15 months for CCR (stratified log-rank p=0.0001, hazard ration 0.58). Two-year survival approximately doubled in the 5-azacytidine arm compared to CCR: 51% vs. 26% (p<0.0001). 5-azacytidine was well tolerated with safety data consistent with previous reports.

Further details can be found in the 5-azacytidine drug information (Appendix F), which contains comprehensive pharmacology, toxicology, pharmacokinetics, pharmacodynamics, metabolism, preclinical, and clinical efficacy and safety data information.[52]

2.6 Rationale for study:

The PD-1/PD-L1 pathway plays a major role in immune evasion and cytotoxic T-cell exhaustion in AML[17, 20, 21]. PD-L1 expression in AML samples is increased at the time of disease progression or after exposure to IFN-gamma[20, 57]. Overexpression of PD-L1 is an independent negative prognostic factor in AML[20]. Hypomethylating agents may alter immune regulation[58]. Yang et al have recently demonstrated that hypomethylating therapy leads to up regulation of PD-L1, PD-1, and PD-L2 gene expression[19]. Patients resistant to hypomethylating therapy had higher increments in gene expression suggesting that PD-1 up regulation may promote resistance to hypomethylating agents. Demethylation of the PD-L1 gene by chronic viral infection results in PD-L1 up-regulation and CTL exhaustion[59]. Similarly, exposure to decitabine up regulates PD-L1, PD-1 and PD-L2 by demethylation of the PD-L1 locus[19]. These data suggest that blockage of PD-1 by nivolumab may improve response and abrogate resistance to hypomethylating agents. The data with the vidaza+nivo combination are encouraging as outlined in detail in section 2.3 with a higher response rate and improved median overall survival as compared to historical

outcomes with azacytidine or decitabine based prior salvage clinical trials performed at MDACC (Daver N, Sharma P et al., ASH 2016, Abst #763), however there is much room for improvement in the response rate and OS. Immune mediated toxicities occur but are being managed with improved awareness and early intervention with steroids and only 2 of 53 patients have come off therapy due to immune mediated organ toxicity. Furthermore, all 15 patients who had an immune mediated grade 2-4 toxicities could continue therapy with nivolumab without recurrence of the IO-toxicity except in one case. The vidaza+nivo is being evaluated in the frontline older (>65 year) patients with AML as well and we have treated 5 patients with no major immune mediated G3/4 toxicities observed. The frontline patients are early for evaluation of response (none have completed > 3 cycles).

Evaluation of multiple costim receptors from BM aspirates and peripheral blood in patients at baseline and every 4-8 weeks on trial by 17-color flow-cytometry in collaboration with the Immunotherapy Platform (Dr James Allison) at MDACC revealed that the CTLA4 on CD8+, T-eff and to a lesser extent on T-reg went up in non-responders after 2 cycles of vidaza+nivolumab suggesting up-regulation of this inhibitory checkpoint as a potential mechanism of resistance. This suggests that simultaneous blockade of the PD1 and CTLA4 may improve the response rate as has been seen in solid tumors. Based on this we propose to evaluate a combination of nivolumab with ipilimumab and vidaza in the first and second salvage and as a frontline approach in older AML patients >/=65 years not candidates for cytotoxic induction therapy.

3.1 STUDY DESIGN

- This will be a phase II, two parallel arm, open-label, non-randomized study with a safety lead-in phase.

- Patients will receive 5-azacytidine subcutaneously or intravenously daily for 7 days of each treatment cycle. For the lead-in portion, the length of the cycle will be at least 28 days to evaluate DLT. Subsequently, cycles will be repeated approximately every 28 days (+/-7 days), and therapy will be continued until clinically significant disease progression or documentation of unacceptable toxicity.

- Arm 1 (Vidaza+Nivolumab): Patients will receive therapy with nivolumab IV infusion on Day 1 and day 14 (+/-3 days) of each 5-azacyidine cycle for first 4 cycles or until CR (whichever occurs earlier) followed by a maintenance regimen (one dose of nivolumab on day 1 of each cycle of 5-azacytdine). Patients who have evidence of budding relapse or seem to have clinical benefit may continue or revert back to receiving nivolumab on Day 1 and day 14 (+/-3 days) of each cycle of 5-azacytidine. Patients may receive nivolumab more or less frequently during the maintenance regimen at the discretion of the treating physician only after discussion with the PI.
Arm 1 includes a salvage cohort and a frontline AML >/= 65 years age cohort.

The salvage cohort of vidaza+nivolumab has completed accrual of 70 patients as of 12/15/16 and will not enroll any more patients at this time. The frontline cohort of vidaza+nivolumab has enrolled 5 patients as of 12/15/16 and will continue to enroll frontline older AML (>65 years) patients.

- Arm 2: Patients will receive therapy with nivolumab IV infusion on Day 1 and day 14 (+/-3 days) of each 5-azacyidine cycle for first 4 cycles or until CR (whichever occurs earlier) followed by a maintenance regimen (one dose of nivolumab on day 1 of each cycle of 5-azacytdine). Patients who have evidence of budding relapse or seem to have clinical benefit may continue or revert back to receiving nivolumab on Day 1 and day 14 (+/-3 days) of each cycle of 5-azacytidine. Patients may receive nivolumab more or less frequently during the maintenance regimen at the discretion of the treating physician only after discussion with the PI. Patients will receive Ipilimumab starting on day 1 and every 6 (or 12) weeks (+/-3 days) there after. The plan is to give a total of 4 doses of the ipilimumab at 6 (or 12) week intervals and then stop therapy. Patients who have evidence of budding relapse or seem to have clinical benefit may continue the ipilimumab every 6 (or 12) weeks.
  Arm 2 includes a salvage cohort and a frontline AML >/= 65 years age cohort. The salvage cohort of vidaza+nivolumab+ipilimumab will have 30 slots and will the priority protocol in salvage setting as the vidaza+nivolumab has already completed enrollment of 70 patients and has no additional slots. The frontline cohort of vidaza+nivolumab+ipilimumab and vidaza+nivolumab will be open simultaneously and we will enroll alternately to these two frontline protocols with close monitoring for futility and toxicity as specified in the predefined statistical efficacy and futility stopping rules in Statistics Section 11.0. This will allow us to closely assess differences in efficacy and toxicity among these two protocols in a similar patient population.

- The historical experience in relapsed/refractory AML patient population is that response with single agent hypomethylator therapy (5-azacytidine or decitabine), when available, is 10-15%. In many instances (eg, patients with relapsed/refractory AML beyond first salvage) no standard therapy is available. Thus, an overall response rate of 30% will be considered significant

- The historical response rate in older newly diagnosed AML (≥65 years) with single agent hypomethylator therapy (5-azacytidine or decitabine) in three large randomized phase II trials was 17.5 % with decitabine alone in patients with AML (Kantarjian et al., JCO 2012), 18% with azacytidine alone in patients with AML and blasts 20-30% (Fenaux et al., JCO 2010), and 27.8% with azacytidine alone in patients with AML and blasts >30% (Dombret et al., Blood 2015). Thus, an overall response rate of ≥40% would be considered significant.

4.1 PATIENT SELECTION

Patients must have baseline evaluations performed prior to the first dose of study drug and must meet all inclusion and exclusion criteria. Results of all baseline evaluations,

which assure that all inclusion and exclusion criteria have been satisfied, must be reviewed by the Principal Investigator or his/her designee prior to enrollment of that patient. In addition, the patient must be thoroughly informed about all aspects of the study, including the study visit schedule and required evaluations and all regulatory requirements for informed consent. The written informed consent must be obtained from the patient prior to initiating treatment or any study-specific procedures. The following criteria apply to all patients enrolled onto the study unless otherwise specified.

4.2 Inclusion Criteria

4.2.1 **Arm 1 Salvage cohort:** Patients with AML or biphenotypic or bilineage leukemia who have failed prior therapy. Patients with AML should have failed prior therapy or have relapsed after prior therapy will be eligible for Arm 1.

Arm 2 Salvage cohort: Patients with AML who have failed up to one prior therapy (i.e. salvage 1 or 2 status) will be eligible for Arm 2 relapse cohort. Allogeneic stem cell transplant for patients in remission at the time of stem cell transplant will not be considered a salvage regimen. Similarly, hydroxyurea if used alone will not be considered a salvage regimen.

Arm 1 and 2 Frontline older cohort:. Patients age 65 years and above with previously untreated AML who are unfit for or decline standard induction therapy. Prior therapy with hydroxyurea, biological or targeted therapy (e.g. FLT3 inhibitors, other kinase inhibitors), or hematopoietic growth factors is allowed, however prior therapy with chemotherapy agents for the disease under study is not allowed. Patients may have received one dose of cytarabine (up to 2 g/m2 administered at presentation for control of hyperleucocytosis.

4.2.2 Patients with MDS or CMML who received therapy for the MDS or CMML and progress to AML are eligible at the time of diagnosis of AML regardless any prior therapy for MDS or CMML. The WHO classification will be used for AML. Prior therapy for MDS or CMML will not be considered as a prior therapy for AML, hence such patients will be considered as frontline AML and eligible for the frontline elderly cohort.

4.2.3 Prior therapy with hydroxyurea, chemotherapy, biological or targeted therapy (e.g. FLT3 inhibitors, other kinase inhibitors), or hematopoietic growth factors is allowed as long as within restrictions outlined in inclusion 4.2.1

4.2.4 Age >/=18 years

4.2.5 Eastern Cooperative Oncology Group (ECOG) Performance Status </=2

4.2.6 Adequate organ function: total bilirubin </= 2 times upper limit of normal (x ULN) (</= 3 x ULN if considered to be due to leukemic involvement or Gilbert's syndrome); aspartate aminotransferase or alanine

aminotransferase </= 2.5 x ULN (</= 5.0 x ULN if considered to be due to leukemic involvement); serum creatinine </= 2 x ULN or GFR>/=50

4.2.7 Patients must provide written informed consent

4.2.8 In the absence of rapidly progressing disease, the interval from prior treatment to time of initiation of 5-azacytidine and nivolumab will be at least 2 weeks OR at least 5 half-lives for cytotoxic/noncytotoxic agents. The half-life for the therapy in question will be based on published pharmacokinetic literature (abstracts, manuscripts, investigator brochure's, or drug-administration manuals) and will be documented in the protocol eligibility document. Since the effect of both nivolumab and 5-azacytidine may be delayed, use of one dose of cytarabine (up to 2 g/m^2) or hydroxyurea for patients with rapidly proliferative disease is allowed before the start of study therapy and during the study treatment. Concurrent therapy for CNS prophylaxis or continuation of therapy for controlled CNS disease is permitted

4.2.9 Females must be surgically or biologically sterile or postmenopausal (amenorrheic for at least 12 months) or if of childbearing potential, must have a negative serum or urine pregnancy test within 72 hours before the start of the treatment

4.2.10 Women of childbearing potential must agree to use an adequate method of contraception during the study and until 3 months after the last treatment. Males must be surgically or biologically sterile or agree to use an adequate method of contraception during the study until 3 months after the last treatment.

Adequate methods of contraception include:

- Total abstinence when this is in line with the preferred and usual lifestyle of the patient. Periodic abstinence (e.g., calendar, ovulation, symptothermal, post-ovulation methods) and withdrawal are not acceptable methods of contraception.
- Female sterilization (have had surgical bilateral oophorectomy with or without hysterectomy) or tubal ligation at least six weeks before taking study treatment. In case of oophorectomy alone, only when the reproductive status of the woman has been confirmed by follow up hormone level assessment
- Male sterilization (at least 6 months prior to screening). For female patients on the study, the vasectomized male partner should be the sole partner for that patient
- Combination of any of the two following (a+b or a+c or b+c)

a. Use of oral, injected or implanted hormonal methods of contraception or other forms of hormonal contraception that have

comparable efficacy (failure rate <1%), for example hormone vaginal ring or transdermal hormone contraception

b. Placement of an intrauterine device (IUD) or intrauterine system (IUS)

c. Barrier methods of contraception: Condom or Occlusive cap (diaphragm or cervical/vault caps) with spermicidal foam/gel/film/cream/ vaginal suppository

In case of use of oral contraception, women should have been stable on the same pill before taking study treatment.

Note: Oral contraceptives are allowed but should be used in conjunction with a barrier method of contraception due to unknown effect of drug-drug interaction.

Women are considered post-menopausal and not of child bearing potential if they have had 12 months of natural (spontaneous) amenorrhea with an appropriate clinical profile (e.g. age appropriate, history of vasomotor symptoms) or have had surgical bilateral oophorectomy (with or without hysterectomy) or tubal ligation at least six weeks ago. In the case of oophorectomy alone, only when the reproductive status of the woman has been confirmed by follow up hormone level assessment is she considered not of child bearing potential.

4.2.11 Patients with GVHD active < grade 2 who are on a stable dose of immunosuppressive therapy (tacrolimus, cyclosporine, or other) for > 2 weeks will be included. Note: Subjects may be using systemic corticosteroids or topical or inhaled corticosteroids.

4.3 Exclusion Criteria

4.3.1 Patients with known allergy or hypersensitivity to nivolumab, ipilimumab, 5- azacytidine, or any of their components.

4.3.2 Patients with a known history of severe interstitial lung disease or severe pneumonitis or active pneumonitis that is uncontrolled in the opinion of the treating physician.

4.3.3 Patients who have previously been treated with nivolumab and/or ipilimumab in combination with 5-azacytidine will be excluded.

4.3.4 Patients with a known history of any of the following autoimmune diseases are excluded: (a) patients with a history of inflammatory bowel disease (including Crohn’s disease and ulcerative colitis) (b) patients with a history of rheumatoid arthritis, systemic progressive sclerosis [scleroderma], Systemic Lupus Erythematosus, autoimmune vasculitis [e.g., Wegener’s Granulomatosis]).

4.3.5 Patients with organ allografts (such as renal transplant) are excluded

4.3.6 Patients with active GVHD > grade 1 will be excluded. Patients with recent increase in the immunosuppressive medication dose within last 2 weeks to control GVHD will not be included. Patients with grade 1 or lower GVHD on </= 10 mg prednisone without any additional immunosuppressive therapies (tacrolimus, prograf, etc) will be eligible.

4.3.7 Patients with symptomatic CNS leukemia or patients with poorly controlled CNS leukemia.

4.3.8 Active and uncontrolled disease/(active uncontrolled infection, uncontrolled hypertension despite adequate medical therapy, active and uncontrolled congestive heart failure NYHA class III/IV, clinically significant and uncontrolled arrhythmia) as judged by the treating physician.

4.3.9 Patients with known Human Immunodeficiency Virus seropositivity will be excluded.

4.3.10 Known to be positive for hepatitis B by surface antigen expression. Known to have active hepatitis C infection (positive by polymerase chain reaction or on antiviral therapy for hepatitis C within the last 6 months)

4.3.11 Any other medical, psychological, or social condition that may interfere with study participation or compliance, or compromise patient safety in the opinion of the investigator

4.3.12 Patients unwilling or unable to comply with the protocol.

4.3.13 Pregnant or breastfeeding

4.3.14 Acute promyelocytic leukemia (APL).

5 <u>TREATMENT PLAN</u>

5.1 General

All patients will be registered through CORe. The objective will be to administer nivolumab and 5-azacytidine OR nivolumab and ipilimumab with 5-azacytidine at established published doses from solid tumor studies. We will first treat 6 patients at dose level 0 in both Arm 1 (Vidaza+nivolumab) and Arm 2 (Vidaza+nivolumab+ipilimumab). Other dose levels will be used for dose adjustments for toxicity during therapy.

5.2 Schedule

The Investigator is responsible for completing the cohort summary template and submitting to the IND office Medical Monitor for review and approval prior to advancing subjects to the next protocol specified cohort/dose level. A copy of the cohort summary should be placed in the Investigator's Regulatory Binder under "sponsor correspondence". This should be submitted after the first six patients.

5.2.1 Patients will be treated according to the following schedule:

- Arm 1: 5-azacytidine will be administered subcutaneously (SQ) or intravenously (IV) for 7 days of every cycle with daily schedule of 7 consecutive days (Days 1-7) or for days 5-2-2 which is 5 consecutive weekdays (Days 1-5) with rest on the 2 weekend days (Days 6 and 7) and then dosing the first two weekdays of the next week (Days 8 and 9) of each 28-day cycle as determined by treating physician. Alternative dosing schedules such as 4-2-3 which is 4 consecutive weekdays (Days 1-4) with rest on the 2 weekend days (Days 5 and 6) and then dosing the first three weekdays of the next week (Days 7, 8, and 9) of each 28-day cycle or other schedules allowing the 2 day weekend interruption may be applied as long as we make every attempt to give the 7 days of azacytidine as consecutively as possible. This must be clearly documented in the medical record as determined by treating physician after approval from the PI. Both SQ and IV forms of administration are FDA approved and considered interchangeable. Patients may start receiving 5-azacytidine by one route and changed to the other, and also administration schedules from one schedule and changed to the other at any time as needed based on patient and/or physician preference.

  Nivolumab will be administered as an approximately 60 minute IV infusion on Day 1 and day 14 (+/-3 days) of each 5-azacyidine cycle for the first 4 cycles or until CR (whichever occurs earlier) followed by a maintenance regimen (one dose of nivolumab on day 1 of each cycle of 5-azacytdine). Patients who have evidence of budding relapse or seem to have clinical benefit may continue or revert back to receiving nivolumab on Day 1 and day 14 (+/-3 days) of each cycle of 5-azacytidine. Patients may receive nivolumab more or less frequently during the maintenance regimen at the discretion of the treating physician only after discussion with the PI. Nivolumab will be administered every cycle with no interruptions unless there are adverse events as described in Section 5.3.

- Arm 2: Azacytidine and nivolumab will be administered as described for Arm 1. Ipilimumab will be administered as a 90 minute IV infusion on day 1 and then every 6 weeks (+/- 3 days) or every 12 weeks (+/- 3 days) depending on the dose level being evaluated during the lead in phase (Table 5B). The Ipilimumab will be planned to be administered for 4 doses but may be continued beyond dose #4 if there is reason to believe this will be of clinical benefit. The ipilimumab may also be resumed if there is evidence of a budding relapse.

- 5-azacytidine at a dose of 75 mg/m2 on days 1-7 or days 5-2-2 has been

shown to be safe in combination with multiple agents including lenalidomide[60], bortezomib[60], and vorinostat[61, 62]. Alternative dosing schedules such as 4-2-3 which is 4 consecutive weekdays (Days 1-4) with rest on the 2 weekend days (Days 5 and 6) and then dosing the first three weekdays of the next week (Days 7, 8, and 9) of each 28-day cycle or other schedules allowing the 2 day weekend interruption may be applied as long as we make every attempt to give the 7 days of azacytidine as consecutively as possible. The maximum dose for this trial of nivolumab 3.0 mg/kg every 2 weeks is the same dose and schedule used in global Phase 3 studies. Based on these data we have proposed the starting dose level 0 of 5-azacytidine 75 mg/m2 x 7 days and nivolumab 3.0 mg/kg on days 1 and 14 of each cycle. The lead-in phase is to ensure that the combination is well tolerated with no unexpected side effects. Nivolumab infusion should begin right after administration of 5-azacytidine on day 1, as described above, whenever possible. The dosing calculations should be based on the body weight. All doses should be rounded to the nearest milligram. There will be no dose reductions (only interruptions when indicated) allowed for nivolumab on this trial.

5.2.1.1 The starting dose will be dose level 0 in both Arm1 and Arm 2.

Table 5A. Arm 1: Dose levels of 5-azacytidine and nivolumab during the lead-in phase (part a)

**Dose level**	**5-azacytidine (mg/m^2/d, Days 1-7)**	**Nivolumab (mg/kg, day 1 and 14)**
-4	25	0.1
-3	50	0.1
-2	50	0.3
-1	75	1.0
**0**	**75**	**3.0 (starting dose)**

The goal of the lead-in phase is to identify the dose at which <2/6 patients experience DLT. During the lead-in phase the dose's of 5- azacytidine and nivolumab may be reduced if 2 or more patients experience DLT at any given dose. After the lead-in phase the dose of nivolumab may not be reduced in the phase II portion of the study. For potential nivolumab related AEs only dose interruptions will be permitted. Dose reductions or escalations of 5-azacytidine are permitted in the phase II portion of the study and dose reductions or escalations of 5-azacytidine beyond those mentioned in table 5A or different to the doses specified should be discussed with the PI and documentation of the justification recorded in the chart.

5.2.1.2 The dose and /or schedule of administration is subject to modification pending information from ongoing clinical trials of nivolumab as a single agent and in combination with other drugs.

5.2.1.3

Table 5B: Arm 2: Dose levels of 5-azacytidine, nivolumab and ipilimumab during the lead-in phase (part a)

**Dose level**	**5-azacytidine (mg/m^2/d, Days 1-7)**	**Nivolumab (mg/kg, day 1 and 14)**	**Ipilimumab q6 weeks**
-2	75	1.0	0.3 mg/kg q6 weeks
-1	75	1.0	1.0 mg/kg q6 weeks
**0 (starting dose)**	**75**	**3.0 (starting dose)**	**1.0 mg/kg q12 weeks**
**+1 (target dose)**	**75**	**3.0 (starting dose)**	**1.0 mg/kg q6 weeks**

The goal of the lead-in phase is to identify the dose at which <2/6 patients experience DLT. During the lead-in phase the dose's of nivolumab and/or ipilimumab may be reduced if 2 or more patients experience DLT at any given dose. After the lead-in phase the dose of nivolumab and ipilimumab may NOT be reduced in the phase II portion of the study. For potential nivolumab and/or ipilimumab related AEs only dose interruptions will be permitted. Dose reductions or escalations of 5-azacytidine are permitted in the phase II portion of the study and dose reductions or escalations of 5-azacytidine beyond those mentioned in table 5B or different to the doses specified should be discussed with the PI and documentation of the justification recorded in the chart.

The dose and /or schedule of administration is subject to modification pending information from ongoing clinical trials of nivolumab or ipilimumab as a single agent and in combination with other drugs.

DLT is defined as clinically significant non-hematologic adverse event or abnormal laboratory value assessed as unrelated to disease progression, intercurrent illness, or concomitant medications and occurring during the first cycle on study that meets any of the following criteria:

- CTCAE Grade 3 AST (SGOT) or ALT (SGPT) for >/= 7 days or grade 3 hyperbiliribunemia for >/= 7 days
- CTCAE Grade 4 AST (SGOT) or ALT (SGPT) of any duration
- All other clinically significant non-hematological adverse event that is Grade 3 or 4 according to the NCI common terminology criteria version 4.0 with the following exceptions:
    - Grade 3 or 4 nausea, vomiting and diarrhea will be considered DLT only if not controlled by optimal therapy.
    - Grade 3 biochemical abnormalities (e.g., lipase or bilirubin

elevation) will only be considered DLT if accompanied by clinical consequences. Grade 3 or 4 electrolyte abnormalities will only be considered DLT if possibly related to study drug and not corrected by optimal replacement therapy.

5.2.1.4 Arm 1: We will first treat 6 patients at dose level 0 in the lead-in phase. If DLT occurs in >/=2/6 patients, this dose level would exceed the MTD, and 6 patients will be treated at the next lower dose level (i.e. dose level -1). The dose de-escalation (Table 5) will continue in cohorts of 6 until we reach a dose level at which <2/6 patients experience a DLT in the first 28 days. The dose level at which 0-1/6 patients experience a DLT in the first 28 days of treatment will be the MTD and will be used to treat an additional patients in the phase II portion of the study. If >/=2/6 patients experience DLT at dose level -4, the study will be revised to consider additional lower dose levels (based on potential synergistic toxicity).

If DLT is observed in the first 28 days of treatment in 0 or 1/6 at dose level 0, then dose level 0 will be used for the phase II portion of the study.

Patients that are removed from study before day 28 for any reason other than toxicity and have not experienced DLT will be replaced.

5.2.1.5 Arm 2: We will first treat 6 patients at dose level 0 in the lead-in phase. If DLT occurs in >/=2/6 patients, this dose level would exceed the MTD, and 6 patients will be treated at the next lower dose level (i.e. dose level -1). If DLT occurs in </=1/6 patients, this dose level would not exceed the MTD, and 6 patients will be treated at the next higher dose level (i.e. dose level +1). The dose escalation or de-escalation (Table 5b) will continue in cohorts of 6 until we reach a dose level at which <2/6 patients experience a DLT in the first 28 days or if </=1/6 patients experience a DLT at dose level +1. The highest dose level at which 0-1/6 patients experience a DLT in the first 28 days of treatment will be the MTD and will be used to treat an additional 24 salvage 1 and 2 patients (Arm 2 Salvage Cohort) and 30 frontline AML >/= 65 years not candidates for induction therapy (Arm 2 Frontline cohort) in the phase II portion of the study. If >/=2/6 patients experience DLT at dose level -2, the study will be revised to consider additional lower dose levels (based on potential synergistic toxicity).

Patients that are removed from study before day 28 for any reason other than toxicity and have not experienced DLT will be replaced.

5.2.2 One cycle of therapy is defined as 28 days. Patients will receive one cycle of therapy every 28 days (+/- 7 days). The ipilimumab will be given every 6 weeks (+/-3 days)

5.2.2.1 In the phase II portion of the study (once the MTD dose has been defined) cycles may be started early (but not earlier than day 21) for patients with active disease if judged in the best interest of the patient.

For the lead-in cohort, DLT defining period is 28 days. As such, cycle 2 should not be started before 28 days for the patients being treated on the lead-in cohort.

5.2.2.2 Subsequent cycles may be delayed for recovery of toxicity or other medical conditions (e.g. infections). Delays in start of subsequent cycles greater than 8 weeks will be acceptable only for patients who are deriving clinical benefit and after discussion with the principal investigator of potential risk/benefit ratio.

5.2.2.3 In instances where one drug has to be discontinued transiently because of safety, the administration of the other drug may continue as scheduled.

5.2.2.4 Subsequent courses of 5-azacytidine and nivolumab OR 5-azacytidine with nivolumab and ipilimumab may be administered regardless of peripheral blood counts during the first 4 cycles and/or in the presence of residual leukemia.

If prolonged myelosuppression (more than 8 weeks) WITH evidence of a hypocellular marrow (marrow cellularity less than 5% without evidence of leukemia) is observed, 5-azacytadine and nivolumab OR 5-azacytidine wih nivolumab and ipilimumab will be discontinued. Delays in start of subsequent cycles greater than 8 weeks will be acceptable only for patients who are deriving clinical benefit and after discussion with the principal investigator of potential risk/benefit ratio. If the peripheral counts do not recover (ANC <1 $x10^9$/L and/or platelets <30 $x10^9$/L) but there is evidence of residual leukemia in the bone marrow, subsequent cycles can be administered at the discretion of the treating physician not earlier than 21 days after the prior cycle.

5.2.2.5 For patients who discontinue therapy, the reason for treatment discontinuation will be captured.

5.2.2.6 Clinical trial update: The safe dose of 5-azacytidine and nivolumab has been established as 5-azacytidine 75mg/m^2 days 1-7 and nivolumab 3.0 mg/kg on days 1 and 14 of each cycle in the ongoing clinical trial. 1/6 patients experienced a DLT at this dose during the safety-run-in phase. All subsequent patients (including relapsed/refractory AML and

newly diagnosed elderly AML) will receive this dose of 5-azacytidine and nivolumab. For the 5-azacytidine with nivolumab and ipilimumab the MTD established from the run-in phase will be used to treat both salvage and frontline patients.

5.3 Dose Adjustments

5.3.1 Nivolumab, Ipilimumab and 5-azacytidine dose adjustments for hematological drug- related adverse events (AE):

Dose reduction/interruption/discontinuation decisions should be based on the CTCAE version 4.0 (Appendix C) of the toxicity and the guidelines provided below.

- Patients with acute leukemia's usually present with abnormal peripheral blood counts at the time therapy is started and myelosuppression is an expected event during the course of therapy for acute leukemia's. Thus, no dose adjustments or treatment interruptions for myelosuppression

  will be planned for the first 4 cycles and/or in the presence of residual leukemia. After that, treatment interruptions and dose adjustments may be considered according to the following guidelines only when there is no evidence of active leukemia (e.g., only if <5% blasts in the bone marrow or cytopenias not considered to be related to leukemia).

  - Patients with a response (no marrow evidence of leukemia) and pre-cycle counts of neutrophils >1x10^9/L and platelets >50 x10^9/L who have sustained low counts of neutrophils <0.5 x10^9/L or a platelet count <20 x 10^9/L for more than 2 consecutive weeks in the current cycle, may have the treatment with 5-azacytidine interrupted at the discretion of the treating physician after discussing with the PI until neutrophils recover to ≥1 x10^9/L and platelets to ≥50 x10^9/L. Nivolumab and ipilimumab have not ben associated with neutropenia and/or thrombocytopenia in prior studies and should generally not be interrupted or discontinued for myelosuppression.

  - If there are persistent peripheral blood blasts, or the bone marrow shows >5% blasts or evidence of leukemia, treatment may be continued regardless of neutrophil and platelet count with supportive care as needed. Dose-interruptions of 5-azacytidine in these patients should be considered on an individual case and discussed with the PI.

  - Patients with a response (no marrow evidence of leukemia) and pre-cycle counts of neutrophils <1x10^9/L and platelets <50 x10^9/L may be continued regardless of neutrophil and platelet

count with supportive care as needed. Dose-interruptions in these patients should be considered on an individual case and discussed with the PI.

- Investigators should, whenever possible, determine which medication is causing the toxicity and interrupt or dose reduce 5-azacytidine and/or interrupt nivolumab and/or ipilimumab or both, as applicable. No dose reductions are permitted for nivolumab and ipilimumab once the RP2D is established. Nivolumab and ipilimumab have not ben associated with neutropenia and/or thrombocytopenia in prior studies and should generally not be interrupted or discontinued for myelosuppression.

5.3.2 Nivolumab, Ipilimumab and 5-azacytidine dose adjustments for non-hematologic drug- related AEs

Investigators should, whenever possible, determine which medication is causing the toxicity and interrupt or dose reduce 5-azacytidine and/or interrupt nivolumab or ipilimumab or both, as applicable. No dose reductions (only interruptions) are permitted for nivolumab and ipilimumab. Dose reductions of azacytidine will be as per Table 5.

Table 6A Arm 1: Dose adjustments for non-hematologic drug-related AEs, clinically significant in the opinion of the investigator

Grade	Occurrence	Dose modification
1 or 2	Any time	No dose reduction
3or 4<br><br>(Persistent grade 2: Consider similar dose adjustments if persistent and not responding to optimal management in the opinion of PI and treating physician)	1st and 2nd time	Hold nivolumab and 5-azacytidine.<br>Resume nivolumab and 5-azacytidine at prior dose if recovery to ≤ Grade 1 occurs within 14 days.<br>If toxicity persists for 15-28 days, hold therapy and resume Nivolumab at prior dose and 5-azacytidine at prior dose if recovery to ≤ Grade 1 OR resume nivolumab at prior dose and 5-azacytidine at ONE dose level below current dose if recovery to ≤ Grade 2.<br>Dose re-escalation to prior dose of 5-azacytidine is permitted in accordance with the dose-escalation guidelines in section 5.3.6.
	3rd and 4th time	Hold nivolumab and 5-azacytidine.<br>Follow until toxicity ≤ Grade 2.<br>Resume nivolumab at prior dose and 5-azacytidine at ONE dose level below current dose.<br>Dose re-escalation of 5-azacytidine to prior dose is permitted in accordance with the dose-escalation guidelines in section 5.3.6..
	5th time	Take patient off the study.

Table 6B Arm 2: Dose adjustments for non-hematologic drug-related AEs, clinically significant in the opinion of the investigator

Grade	Occurrence	Dose modification
1 or 2	Any time	No dose reduction
3or 4<br><br>(Persistent grade 2: Consider similar dose adjustments if persistent and not responding to optimal management in the opinion of PI and treating physician)	1st and 2nd time	Hold nivolumab, ipilimumab and 5-azacytidine.<br>Resume nivolumab and/or ipilimumab and 5-azacytidine at prior dose if recovery to ≤ Grade 1 occurs within 14 days.<br>If toxicity persists for 15-28 days, hold therapy and resume nivolumab and/or ipilimumab at prior dose and 5-azacytidine at prior dose if recovery to ≤ Grade 1 OR resume nivolumab and/or ipilimumab at prior dose and 5-azacytidine at ONE dose level below current dose if recovery to ≤ Grade 2.<br>Dose re-escalation to prior dose of 5-azacytidine is permitted in accordance with the dose-escalation guidelines in section 5.3.6.
	3rd and 4th time	Hold nivolumab and ipilimumab and 5-azacytidine.<br>Follow until toxicity ≤ Grade 2.<br>Resume nivolumab and ipilimumab at prior dose and 5-azacytidine at TWO dose level below current dose.<br>Dose re-escalation of 5-azacytidine to prior dose is permitted in accordance with the dose-escalation guidelines in section 5.3.6..
	5th time	Take patient off the study.

5.3.3 Nivolumab or Ipilimumab or both dose delay/interruption for immune-oncology drug-related AEs, clinically significant in the opinion of the investigator

Nivolumab (Arm 1) or nivolumab+ipilimumab (Arm 2) administration should be delayed for the following drug-related AEs, clinically significant in the opinion of the investigator:

- Any Grade >/=2 non -skin AE, except that
  - Grade 2 fatigue or laboratory abnormalities do not require delay, however
    - ♦ Patients with ALT or AST > 3 and up to 5 x ULN or total bilirubin greater > 1.5 and up to 3 x ULN or creatinine >1.5 and up to 6 x ULN (or greater than 1.5 times baseline) should have nivolumab (Arm 1) or nivolumab+ipilimumab (Arm 2) treatment withheld.
- Any Grade 3 skin AE, or Grade 3 laboratory abnormality, with the following exceptions:
  - – Grade 3 lymphopenia or leukopenia does not require dose delay.
  - – However, patients with ALT or AST >5 x ULN or total bilirubin greater > 3 x ULN or creatinine >6 x ULN should be discontinued

from nivolumab treatment (Arm 1) or nivolumab and ipilimumab treatment (Arm 2).

- Any AE, laboratory abnormality, or intercurrent illness, which in the judgment of the investigator, warrants delaying the dose of study medication.
- Nivolumab and ipilimumab dose reductions are not permitted in this study (only dose delays when indicated).

Subjects may resume treatment with nivolumab alone (Arm 1) or nivolumab and ipilimumab (Arm 2) with study drug when the drug-related AE(s) resolve to Grade ≤1 or baseline value, with the following exceptions:

- Subjects may resume treatment in the presence of Grade 2 fatigue
- Subjects who have not experienced a Grade 3 drug-related skin AE may resume treatment in the presence of Grade 2 skin toxicity
- Drug-related pulmonary toxicity, diarrhea, or colitis, must have resolved to baseline before treatment is resumed
- Drug-related endocrinopathies adequately controlled with only physiologic hormone replacement may resume treatment

  If the criteria to resume treatment are met, the subject should restart treatment at the next scheduled timepoint per protocol. However, if the treatment is delayed past the next scheduled timepoint per protocol, the next scheduled timepoint will be delayed until dosing resumes. Patients who receive combination therapy in whom continuation of 5-azacytidine is considered to be inadequate, or inappropriate (e.g., because of pancytopenia) can discontinue 5-azacytidine and continue with nivolumab only.

  If treatment is delayed > 8 weeks, the subject must be permanently discontinued from study therapy, except as specified in discontinuation section.

5.3.4 Nivolumab (Arm 1) or nivolumab+ipilimumab (arm 2) Discontinuation Criteria

Treatment with IO-agent/s should be permanently discontinued for the following:

- Any Grade 2 drug-related uveitis or eye pain or blurred vision that does not respond to topical therapy and does not improve to Grade 1 severity within the re-treatment period OR lasting > 7 days with systemic treatment.

- Any Grade 3 non-skin, drug-related adverse event lasting > 7 days, with the following exceptions for drug-related laboratory abnormalities, uveitis, pneumonitis, bronchospasm, diarrhea, colitis, neurologic adverse event, hypersensitivity reactions, and infusion reactions:
  - Grade 3 drug-related uveitis, pneumonitis, bronchospasm, diarrhea, colitis, neurologic adverse event, hypersensitivity reaction, or infusion reaction of any duration requires discontinuation

  - Grade 3 drug-related laboratory abnormalities do not require treatment discontinuation except those noted below:

- Any drug-related liver function test (LFT) abnormality that meets the following criteria require discontinuation: ALT or AST >5 x ULN or total bilirubin greater > 3 x ULN or creatinine >6 x ULN

- Any Grade 4 drug-related adverse event or laboratory abnormality, except for the following events which do not require discontinuation:
  - Isolated Grade 4 amylase or lipase abnormalities that are not associated with symptoms or clinical manifestations of pancreatitis and decrease to < Grade 4 within 1 week of onset.

  - Isolated Grade 4 electrolyte imbalances/abnormalities that are not associated with clinical sequelae and are corrected with supplementation/appropriate management within 72 hours of their onset

- Any dosing interruption lasting > 8 weeks with the following exceptions:
- Dosing interruptions to allow for prolonged steroid tapers to manage drug-related adverse events are allowed. Prior to re-initiating treatment in a subject with a dosing interruption lasting > 8 weeks, the Investigator must be consulted. Tumor/Leukemia assessments should continue as per protocol even if dosing is interrupted.

- Dosing interruptions > 8 weeks that occur for non-drug-related reasons may be allowed if approved by the Investigator. Prior to re-initiating treatment in a subject with a dosing interruption lasting > 8 weeks, the Investigator must be consulted. Tumor/Leukemia assessments should continue as per protocol even if dosing is interrupted.

- Delays in start of subsequent cycles greater than 8 weeks will be acceptable only for patients who are deriving clinical benefit and after discussion with the principal investigator of potential risk/benefit ratio.

5.3.5 Detailed management algorithms for immune-oncology drug-related adverse events (including gastrointestinal, renal, pulmonary, hepatic, endocrine, skin and neurological) are provided in Appendix G. These general guidelines constitute guidance to the Investigator and may be supplemented by discussions with the Medical Monitor representing the Sponsor in specific cases.

5.3.6 Intra-patient dose re-escalation for patients who have had dose-reductions due to hematological or non-hematological toxicity:

Once the MTD has been established in the lead-in, the nivolumab dose (Arm 1) or nivolumab+ipilimumab dose (Arm 2) will not be reduced or escalated during the phase II portion. Intra-patient dose re-escalation of 5-azacytidine (in accordance with the dosing schema in table 5) will be permitted provided:

- Patient has completed >/=1 cycle at their current dose level
- Patient has not experienced any grade 3 or higher non-hematologic drug-related toxicity, and
- Patient has not experienced drug-related hematologic DLT, and
- At least 6 patients have been treated at the next higher dose level and followed for at least 28 days without experiencing DLT.
- The dose may be escalated by one dose level per cycle (per table 1) provided such dose level does not exceed the established MTD or dose level 0, whichever is lower. Dose level 0 is the maximum dose allowed on this study. No dose escalation beyond dose level 0 will be permitted.

5.3.7 Modifications of dose schedules other than the above will be allowed within the following guidelines:

5.3.7.1 Further dose reductions can be made to keep clinically significant toxicities grade ≤ 2.

5.3.7.2 Dose adjustments by more than 1 dose level at a time (e.g., from 5-azacytidine 75 mg/m^2 to 25 mg/m^2) can be considered when judged in the best interest of the patient (e.g. severe myelosuppression) when toxicity has resolved. The reason for this reduction will be discussed with the PI or Co-PI and documented in the medical record.

5.3.7.3 A patient who has had a dose reduction because of any of the reasons mentioned above may have their dose escalated provided the patient has remained free of toxicity requiring dose adjustments as defined above for at least 1 month. Escalation will be made by 1 dose-level increment only, and not more frequent than every month.

5.3.7.4 Treatment interruptions and dose modifications other than the ones mentioned above can be considered after discussion with the PI and proper documentation of the rationale.. Dose adjustment/delay of only one of the agents is permissible if the toxicity is most likely judged to be related to one of the agents by the investigator (e.g., in patients with autoimmune thyroiditis or autoimmune hepatitis this would be likely secondary to nivolumab or nivolumab+ipilimumab, in patients with cytopenia’s this would be likely secondary to 5-azacytidine).

5.4 Duration of Therapy

In the absence of treatment delays due to adverse events, treatment may continue until one of the following criteria applies:

1. Clinically significant progressive disease, or

2. Intercurrent illness that prevents further administration of treatment, or

3. Patient request, or

4. General or specific changes in the patient's condition render the patient unacceptable for further treatment in the judgment of the investigator, or

5. Unacceptable toxicity that in the opinion of the investigator makes it unsafe to continue therapy.

5.4.1 It is planned that up to a total of 24 cycles of therapy will be administered for patients deriving benefit from this regimen. Continuation of therapy for patients completing 24 cycles of therapy may be considered on a case-by-case basis after discussion with the principal investigator.

5.4.2. A minimum of 1 full course (defined as the administration of 5-azacytidine for 7 days and one dose of nivolumab) will be required for a patient to be considered as having received an adequate trial to evaluate efficacy. All patients receiving at least one dose of any of the two drugs will be considered evaluable for toxicity.

5.5 Supportive Care:

Supportive care measures including blood products, infection prophylaxis and growth factors will be administered according to institutional and Leukemia Department guidelines.

Management Algorithms for Treatment of Nivolumab or Ipilimumab Related Adverse Events Immuno-oncology (I-O) agents are associated with AEs that can differ in severity and duration than AEs caused by other therapeutic classes. Nivolumab and ipilimumab are considered immuno-oncology agent in this protocol. Early recognition and management of AEs associated with immuno-oncology agents such as nivolumab and/or ipilimumab may mitigate severe toxicity. Nivolumab and ipilimumab have a known safety profile however, a general principle is that differential diagnoses should be diligently evaluated according to standard medical practice. Non- inflammatory etiologies should be considered and appropriately treated. Corticosteroids are a primary therapy for immuno-oncology drug-related adverse events, and nivolumab or ipilimumab no exception. The oral equivalent of the recommended IV doses may be considered for ambulatory patients with low-grade toxicities. The lower bioavailability of oral corticosteroids should be taken into account when switching to the equivalent dose of oral corticosteroids.

Management Algorithms have been developed to assist investigators in

assessing and managing the following groups of adverse events: **Endocrinopathy, Gastrointestinal, Hepatic, Neurological Pulmonary, Renal and Skin**. These algorithms are found in the “Nivolumab Investigator Brochure” (Appendix E), Ipilimumab Investigator Brochure (Appendix J) and “Management algorithms for immuno-oncology drug-related adverse events” (Appendix G) of this protocol. The guidance provided in these algorithms should not replace the Investigator’s medical judgment but should complement it.

Finally, consultation with a medical or surgical specialist, especially prior to an invasive diagnostic or therapeutic procedure, is highly recommended.

5.6 Concomitant Medications:

Consistent with subject safety and comfort, administration of any prescription or over-the-counter drug products other than study medication should be minimized during the study period. Subjects should be discouraged from use of street drugs, herbal remedies, self-prescribed drugs, tobacco products, or excessive alcohol during the clinical study.

If considered necessary for the subject’s wellbeing, drugs for concomitant medical conditions or for symptom management may be given at the discretion of the investigator. The investigator’s decision to authorize the use of any drug other than study drug should take into account subject safety, the medical need, the potential for drug interactions, the possibility for masking symptoms of a more significant underlying event, and whether use of the drug will compromise the outcome or integrity of the study.

Subjects should be instructed about the importance of the need to inform the clinic staff of the use of any drugs or remedies (whether prescribed, over-the-counter, or illicit) before and during the course of the study.

Recommendations with regard to specific types of concomitant therapies, supportive care; diet and other interventions are as follows:

Concomitant medications are recommended as prophylaxis for nausea, vomiting, and infections, and are allowed for managing myelosuppression as shown in Table 8. Myelosuppression is expected in patients with AML due to underlying disease, as well as due to chemotherapy (such as 5-azacytidine), or both. Most patients have neutropenia, thrombocytopenia, or both at study entry. Significant or life-threatening myelosuppression may be managed with growth factor support and blood transfusion according to institutional standard of care, American Society of Clinical Oncology (ASCO) Practice Guidelines, and/or NCCN Practice Guidelines.

Infections secondary to myelosuppression are common in patients with AML, and may be related to underlying disease, chemotherapy, or both. Therefore, the use of prophylactic antibiotics, antifungal agents, and antiviral agents is

recommended according to institutional standards.

Since the effect of both nivolumab and 5-azacytidine may be delayed, patients with high WBC counts may receive hydroxyurea and up to 1 dose of cytarabine (up to 2 g/m^2) prior to study entry. Hydroxyurea is allowed before the start of study therapy and during the study treatment. Hydrea and cytarabine use would be recorded in the CRF. Concurrent therapy for CNS prophylaxis or continuation of therapy for controlled CNS disease is permitted. With the exception of these agents, concomitant systemic chemotherapy or radiation therapy is not permitted. Subjects are not allowed to participate concurrently in any other therapeutic clinical study.

Subjects may be receiving systemic corticosteroids (daily doses ≤10 mg of prednisone or equivalent if indicated for adrenal replacement or antiemetic therapy), topical, or inhaled corticosteroids at study enrollment. They may receive systemic, topical, inhaled, or enteric corticosteroids while on study without limitation if they develop conditions that require corticosteroid therapy; such subjects are not required to discontinue study participation.

All ongoing medications and therapies (including herbal products, nutritional supplements, and nontraditional medications) at screening will be considered prior medications. Concomitant medication data will not be collected or entered into the case report form other than hydrea and cytarabine as mentioned above; however, the subject's medication record will contain a list of concomitant medications. If a prohibited medication is inadvertently administered/ taken by the patient, the patient may remain on study as long as the prohibited medication is discontinued as soon as feasible. If a prohibited medication is considered essential for the patient well being, continuation on study with concomitant administration of such medication(s) will need to be discussed with and approved by the principal investigator and medical monitor.

Investigational agents that are not used for treatment of the leukemia per se (e.g. anti-infective prophylaxis or therapy) will be allowed. Other supportive care studies are allowed, even if under an IND.

Table 8: Instructions for the use of concomitant medications and therapies

Category of Use	Medication	Comment on Use	Restriction on Use
Recommended	Prophylactic antibiotics, antifungal agents, and antiviral agents	Strongly encouraged	None
	Antiemetic agents	According to standard of care at MDACC	None
Allowed	Oral allopurinol or rasburicase	At investigators discretion	None
	Leukapheresis	According to standard of care at MDACC	Before induction 1 day 1 only
	Red blood cell transfusion	None	None

	Platelet transfusion	None	None
	White blood cell transfusion	At investigators discretion according to standard of care at MDACC	None
	Myeloid growth factors or platelet growth factor	At investigators discretion according to standard of care at MDACC	None
	Erythropoietin or darbepoetin	At investigators discretion	None
	Any other medication for supportive care	At investigators discretion according to standard of care at MDACC	None

MDACC = MD Anderson Cancer Center

6.0 STUDY MEDICATIONS

6.1 Nivolumab (Anti-PD1)

Nivolumab is a fully human, IgG4 (kappa) isotype, monoclonal antibody that binds PD-1. Nivolumab will be supplied in vials of 100 mg (10 mg/mL) and packaged in an open-label fashion. Ten nivolumab vials (each 10 mL) will be packaged within a carton. The vials are not subject specific although there will be specific vial assignments by subject distributed by the Pharmacy in order to track drug usage and re-supply.

6.1.1 Dose Calculation of nivolumab

Total dose should be calculated as in the following example:

Subject’s actual body weight in kg x MTD in mg = total dose in mg

For example, a subject weighing 70 kg who is scheduled receive a dose of 3 mg/kg would be administered 210 mg of nivolumab (70 kg x 3 mg/kg = 210 mg).

6.1.2 Preparation and Dispensing of Nivolumab

The product storage manager should ensure that the study drug is stored in accordance with the environmental conditions (temperature, light, and humidity) as determined by the Investigator Brochure. If concerns regarding the quality or appearance of the study drug arise, do not dispense the study drug and contact the sponsor immediately.

Investigational product documentation must be maintained that includes all processes required to ensure drug is accurately administered. This includes documentation of drug storage, administration and, as applicable, storage temperatures, reconstitution, and use of required processes (e.g. required diluents, administration sets).

Nivolumab vials must be stored at a temperature of 2°C to 8°C and should be protected from light. If stored in a glass front refrigerator, vials should be stored in the carton. Recommended safety measures for preparation and handling of nivolumab include laboratory coats and gloves.

For details on prepared drug storage and use time of nivolumab under room temperature/light and refrigeration, please refer to the Investigator Brochure section for "Recommended Storage and Use Conditions". Care must be taken to assure sterility of the prepared solution, as the product does not contain any anti-microbial preservative or bacteriostatic agent. No incompatibilities between nivolumab and polyolefin bags have been observed.

Nivolumab is to be administered as an approximately 60 minute IV infusion, using a volumetric pump with a 0.2/0.22 micron in-line filter at the protocol-specified dose. Variations in infusion times due to minor differences in IV bag overfill/underfill and institutional procedure on flushing chemotherapy lines will not result in protocol deviation. All infusion times are considered approximate. The drug can be diluted with 0.9% normal saline for delivery but the total drug concentration of the solution cannot be below 0.35 mg/ml. It is not to be administered as an IV push or bolus injection. At the end of the infusion, flush the line with a sufficient quantity of normal saline.

6.1.3 Administration of nivolumab

Patients will receive nivolumab as an approximately 60 minute IV infusion on Day 1 and day 14 (+/-3 days) of a treatment cycle every 28 days for 4 cycles or until CR (whichever occurs earlier) followed by a maintenance regimen (one dose of nivolumab on day 1 of each cycle of 5-azacytidine). Dosing calculations should be based on the body weight assessed at the start of each cycle as described above. All doses should be rounded to the nearest milligram. The screening body weight may be used for dosing of cycle 1.

6.1.4 Patient Monitoring During Infusion

During the first infusion patient vital signs should be monitored prior to dosing, approximately every 30 minutes after the initiation of the infusion, and up to approximately 60 minutes after the completion of the infusion, or longer if indicated, until the vital signs normalize or return to baseline. For subsequent infusions, vital signs should be collected prior to dosing, and at the completion of the infusion. If the patient experiences and infusion reaction, the patient will be monitored up to 60 minutes after the completion of the infusion, or longer if indicated, until the vital signs normalize or return to baseline. A+/- 30 minute window applies to all vital sign time points.

6.1.5 Treatment of nivolumab Related Infusion Reactions

Since nivolumab contains only human immunoglobulin protein sequences, it is unlikely to be immunogenic and induce infusion or hypersensitivity reactions. However, if such a reaction were to occur, it might manifest with fever, chills, rigors, headache, rash, pruritis, arthralgia's, hypo- or hypertension, bronchospasm, or other symptoms. All Grade 3 or 4 infusion reactions should be reported within 24 hours to the BMS Medical Monitor and reported as an SAE if criteria are met. Infusion reactions should be graded according to NCI CTCAE (version 4.0) guidelines.

Treatment recommendations for nivolumab related infusion reactions are provided below and may be modified based on MD Anderson treatment standards and guidelines, as appropriate:

For Grade 1 symptoms (Mild reaction; infusion interruption not indicated; intervention not indicated): Remain at bedside and monitor subject until recovery from symptoms. The following prophylactic premedications are recommended for future infusions: diphenhydramine 50 mg (or equivalent) and/or acetaminophen 325 to 1000 mg at least 30 minutes before additional nivolumab administrations.

For Grade 2 symptoms (Moderate reaction requires therapy or infusion interruption but responds promptly to symptomatic treatment [e.g. antihistamines, non-steroidal anti-inflammatory drugs, narcotics, corticosteroids, bronchodilators, IV fluids]; prophylactic medications indicated for ≤ 24 hours):

Stop the nivolumab infusion, begin an IV infusion of normal saline, and treat the subject with diphenhydramine 50 mg IV (or equivalent) and/or acetaminophen 325 to 1000 mg; remain at bedside and monitor subject until resolution of symptoms. Corticosteroid or bronchodilator therapy may also be administered as appropriate. If the infusion is interrupted, then restart the infusion at 50% of the original infusion rate when symptoms resolve; if no further complications ensue after 30 minutes, the rate may be increased to 100% of the original infusion rate. Monitor subject closely. If symptoms recur then no further nivolumab will be administered at that visit. Administer diphenhydramine 50 mg IV, and remain at bedside and monitor the subject until resolution of symptoms. The amount of study drug infused must be recorded on the case report form (CRF). The following prophylactic premedications are recommended for future infusions: diphenhydramine 50 mg (or equivalent) and/or acetaminophen 325 to 1000 mg should be administered at least 30 minutes before additional nivolumab administrations. If necessary, corticosteroids (recommended dose: up to 25 mg of IV hydrocortisone or equivalent) may be used.

For Grade 3 or Grade 4 symptoms [Severe reaction, Grade 3: prolonged (i.e. not rapidly responsive to symptomatic medication and/or brief interruption of infusion); recurrence of symptoms following initial improvement; hospitalization indicated for other clinical sequelae (e.g. renal impairment, pulmonary infiltrates), Grade 4: life-threatening; pressor or ventilatory support indicated]:

Immediately discontinue infusion of nivolumab. Begin an IV infusion of normal saline, and treat the subject as follows: Recommend bronchodilators, epinephrine 0.2 to 1 mg of a 1:1,000 solution for subcutaneous administration or 0.1 to 0.25 mg of a 1:10,000 solution injected slowly for IV administration, and/or diphenhydramine 50 mg IV with methylprednisolone 100 mg IV (or equivalent), as needed. Subject should be monitored until the investigator is comfortable that the symptoms will not recur. Nivolumab will be permanently discontinued. Institutional guidelines will be followed for the treatment of anaphylaxis. Remain at bedside and monitor subject until recovery from symptoms. In the case of late-occurring hypersensitivity symptoms (e.g. appearance of a localized or

generalized pruritus within 1 week after treatment), symptomatic treatment may be given

(e.g. oral antihistamine or corticosteroids).

For further details regarding dose-calculation of nivolumab, preparation and dispensing of nivolumab, administration of nivolumab, patient monitoring during infusion and treatment of nivolumab related infusion reactions please see the dosing procedure manual (Appendix I).

6.2 Azacytidine:

5-azacytidine will be commercially obtained. Standard procedures should be used for preparation and administration of 5-azacytidine. The following guidelines are suggested:

Vidaza® (5-azacytidine) (is a cytotoxic drug and, as with other potentially toxic compounds, caution should be exercised when handling and preparing Vidaza® (5-azacytidine) suspensions.

If reconstituted Vidaza® (5-azacytidine) comes into contact with the skin, immediately and thoroughly wash with soap and water. If it comes into contact with mucous membranes, flush thoroughly with water.

The Vidaza® (5-azacytidine) vial is single-use and does not contain any preservatives. Unused portions of each vial should be discarded properly. See **Handling and Disposal.** Do not save any unused portions for later administration.

6.2.1 Preparation for Subcutaneous Administration: Vidaza® (5-azacytidine) should be reconstituted aseptically with 4 mL sterile water for injection. The diluent should be injected slowly into the vial. Vigorously shake or roll the vial until a uniform suspension is achieved. The suspension will be cloudy. The resulting suspension will contain 5-azacytidine 25 mg/mL.

6.2.1.1 Preparation for Immediate Subcutaneous Administration: Doses greater than 4 mL should be divided equally into 2 syringes. The product may be held at room temperature for up to 1 hour, but must be administered within 1 hour after reconstitution.

6.2.1.2 Preparation for Delayed Subcutaneous Administration: The reconstituted product may be kept in the vial or drawn into a syringe. Doses greater than 4 mL should be divided equally into 2 syringes. The product must be refrigerated immediately, and may be held under refrigerated conditions (2ºC–8ºC, 36ºF–46ºF) for up to 8 hours. After removal from refrigerated conditions, the suspension may be allowed to equilibrate to room temperature for up to 30 minutes prior to administration.

6.2.1.3 Subcutaneous Administration: To provide a homogeneous

suspension, the contents of the syringe must be re-suspended by inverting the syringe 2–3 times and vigorously rolling the syringe between the palms for approximately 30 seconds immediately prior to administration. Vidaza® (5-azacytidine) suspension is administered subcutaneously. Doses greater than 4 mL should be divided equally into 2 syringes and injected into 2 separate sites. Rotate sites for each injection (thigh, abdomen, or upper arm). New injections should be given at least 1 inch from an old site and never into areas where the site is tender, bruised, red, or hard.

6.2.1.4 Suspension Stability: 5-azacytidine reconstituted for subcutaneous administration may be stored for up to 1 hour at 25°C (77°F) or for up to 8 hours between 2°C and 8°C (36°F and 46°F).

6.2.2 Preparation for Intravenous Administration: Reconstitute the appropriate number of Vidaza® (5-azacytidine) vials to achieve the desired dose. Reconstitute each vial with 10 mL sterile water for injection. Vigorously shake or roll the vial until all solids are dissolved. The resulting solution will contain 5-azacytidine 10mg/mL. The solution should be clear. Parenteral drug product should be inspected visually for particulate matter and discoloration prior to administration, whenever solution and container permit.

Withdraw the required amount of Vidaza® (5-azacytidine) solution to deliver the desired dose and inject into a 50-100 mL infusion bag of either 0.9% Sodium Chloride Injection or Lactated Ringer’s Injection.

6.2.2.1 Intravenous Solution Incompatibility: Vidaza® (5-azacytidine) is incompatible with 5% Dextrose solutions, Hespan, or solutions that contain bicarbonate. These solutions have the potential to increase the rate of degradation of Vidaza® (5-azacytidine) and should therefore be avoided.

6.2.2.2 Intravenous Administration: Vidaza® (5-azacytidine) solution is administered intravenously. Administer the total dose over a period of 10-40 minutes. The administration must be completed within 1 hour of reconstitution of the VIDAZA vial.

6.2.2.3 Solution Stability: Vidaza® (5-azacytidine) reconstituted for intravenous administration may be stored at 25°C (77°F), but administration must be completed within 1 hour of reconstitution.

6.2.3 Storage: Store unreconstituted vials at 25º C (77º F); excursions permitted to 15º-30º C (59º-86º F) (See USP Controlled Room Temperature). There

is no need to protect 5-azacytidine from exposure to light.

6.2.4 Handling and Disposal: Procedures for proper handling and disposal of anticancer drugs should be applied.

6.3 Ipilimumab: Please see attached Ipilimumab updated IB for Study Medication information.

6.3.1 Variations in infusion times of nivolumab or 5-azacytidine due to minor differences in IV bag overfill/underfill and institutional procedure on flushing chemotherapy lines will not result in protocol deviation. All infusion times are considered approximate.

6.3.2 Unused or expired nivolumab and 5-azacytidine will be safely disposed according to MD Anderson pharmacy standard guidelines.

6.3.3 The administration time for each drug may vary by +/- 30 minutes from the specified time period for the infusion of each agent, without constituting a deviation to the study.

7.1 PATIENT EVALUATION

Every effort will be made to adhere to the schedule of events and all protocol requirements. Variations in schedule of events and other protocol requirements that do not affect the rights and safety of the patient will not be considered as deviations. Such variations may include laboratory assessments completed outside of schedule, occasional missed required research samples such as correlative assays.

7.2 Pre-Treatment Evaluation

All pretreatment studies should be obtained within 14 days of entry into the trial, unless otherwise stated.

7.2.1 A complete history and physical, documentation of all measurable disease, concomitant medications and performance status.

7.2.2 CBC, platelet count, differential (differential can be omitted if WBC is $\leq$0.5 x10^9/L).

7.2.3 Creatinine, total bilirubin, ALT or AST.

7.2.4 Pregnancy test (urine or plasma) in females of childbearing potential should be performed within 72 hours before initiation of study.

7.2.5 Bone marrow aspirate during the last 28 days preceding study initiation. Cytogenetics will be obtained prior to therapy (results from prior analysis can be used for this purpose).

7.2.6 Pretreatment optional correlative studies (see below)

7.2.7 Respiratory PCR panel by Nasal Wash (rationale: to rule out underlying

viral infection that may be exacerbated by Nivolumab)

7.2.8 Chest Xray (rationale: to rule out underlying pneumonitis that may be exacerbated by Nivolumab

7.2.9 Routine Urine analysis (UA)

7.2.10 TSH, free T3, free T4, total cortisol

7.2.11 Respiratory PCR panel
7.2.12 EKG at baseline

7.3 Evaluation During Treatment

7.3.1 Physical exam at the start of each cycle (± 4 days) and documentation of all concomitant medications.

7.3.2 CBC, platelet count, differential once weekly (±4 days) for the first 3 cycles, then every 2-4 weeks (differential can be omitted if WBC is ≤0.5 $x10^9/L$)

7.3.3 Creatinine, total bilirubin, ALT, or AST once weekly (±4 days) for the first 3 cycles, then every 2-4 weeks.

7.3.4 TSH, free T3, free T4, total cortisol once monthly (±4 days) for the first 3 cycles, then every 3 cycles (±4 days).

7.3.5 EKG once monthly on Day 1 (+/-4 days) of each cycle

7.3.6 Bone marrow aspiration on day 28 (+/- 7 days), then every 1-3 cycles. Bone marrow tests can be ordered more frequently if mandated by development of peripheral blood counts. No repeat bone marrow is necessary if nonresponse or progressive disease can be unequivocally diagnosed from peripheral blood tests or, in patients with a WBC < 0.3 if the bone marrow test is considered noncontributory by the investigator at any time point.

7.3.7 Concomitant medication data will not be collected or entered into the case report form except for concomitant hydroxyurea and cytarabine; however, the subject's medication record will contain a list of concomitant medications.

7.3.8 Correlative Studies relating to immunologic response (Optional)**:** Tumor tissue, blood samples and bone marrow aspirate will be collected on a separate IRB approved laboratory protocol for immune monitoring as previously published[63-66], under the supervision of the Immunotherapy Platform (IMT lab Protocol PA13-0291). Patients will be consented separately on the IRB approved consent for protocol PA 13-0291. Patients

may participate in protocol 2014-0861 irrespective of whether they choose to participate on protocol PA 13-0291. In tumor tissues, immunohistochemical studies will be performed to evaluate tumor and immunological cell markers such as CD4 and CD8 T cells. In peripheral blood, we will also evaluate tumor and immune cell populations including but not limited to CD4 and CD8 T cells in pre and post therapy samples.

Peripheral blood up to 45 mL (within 24 hours) will be collected under an IRB-approved laboratory protocol (IMT lab protocol PA 13-0291) for testing of biomarkers at the following time points
-Baseline (prior to 5-azacytidine dose), on day 14 (prior to nivolumab), and between days 21 to 28 on cycle 1 (done at MD Anderson).
-In each subsequent cycle blood samples will be obtained on day 1 (prior to 5-azacytidine dose) and day 14 (prior to nivolumab dose) when possible.
-Samples will be collected at progression whenever possible.

Bone Marrow:
-Bone marrow samples will be collected under an IRB-approved laboratory protocol for testing of biomarkers at baseline, at day 28 (+/-7 days), then every 1-3 cycles, and at progression. All correlative samples are optional.

All these samples can be obtained +/- 3 days. Missed samples for correlative studies will not constitute protocol deviations.

7.3.9 For patients that remain on study with no significant toxicity for more than 6 months, subsequent evaluations during study may be modified after discussion with the principal investigator. These include a decrease in frequency of bone marrow aspirations to every 6-12 months (or as clinically indicated), correlative studies to every 6-12 months (or suspension of sample collection for correlative studies), other laboratory tests to once every cycle.

7.3.10 ALL treatments with nivolumab and/or ipilimumab must be administered at the MDACC outpatient clinic. The first cycle of azacytidine must be fully administered at the MDACC outpatient clinic. Starting cycle #2, patients will have the option of receiving 5-azacytidine injections or infusions at the MDACC outpatient clinic or local ambulatory treatment center. We do not intend for the subjects to receive the nivolumab or the ipilimumab at an outside physician's office at any time on the study. During the first cycle all the laboratory evaluations will be done at MDACC. Subsequently, the patient may have the laboratory work done at a local clinic and the results reported to the research nurse for the study. The laboratory work done at the local clinic will be forwarded to the patient's attending physician at MDACC or PI of the study, who will sign off on the labs to verify that the results have been reviewed. Patient should have end of cycle visit here at MDACC and/or prior to starting next cycle (+/- 4 days).

Outside Physician Participation During Treatment

1. MDACC Physician communication with the outside physician is required prior to the patient returning to the local physician.
This will be documented in the patient record.

2. A letter to the local physician outlining the patient's participation in a clinical trial will request local physician agreement to supervise the patient's care (Appendix H).

3. Protocol required evaluations outside MDACC will be documented by telephone, fax or e-mail. Fax and/or e-mail will be dated and signed by the MDACC physician, indicating that they have reviewed it. Changes in drug dose and/or schedule must be discussed with and approved by the MDACC physician investigator, or their representative prior to initiation, and will be documented in the patient record.

4. A copy of the informed consent, protocol abstract, treatment schema and evaluation during treatment will be provided to the local physician.

5. Documentation to be provided by the local physician will include progress notes, reports of protocol required laboratory and diagnostic studies and documentation of any hospitalizations.

6. The home physician will be requested to report to the MDACC physician investigator all life threatening events within 24 hours of documented occurrence.

7. All follow-up visits will be performed at MDACC.

8. Changes in drug dose and/or schedule must be discussed with and approved by the MDACC physician investigator, or their representative prior to initiation, and will be documented in the patient record.

Study Period/ Cycle	Scre	Treatment													End of Study (EOS)[h]
		Cycle 1				Cycle 2				Cycle 3				Subsequent Cycles	
**Cycle Day**		**1**	**8**	**15**	**22**	**1**	**8**	**15**	**22**	**1**	**8**	**15**	**22**	**1**	
**Study Day**	**-14 to**	**1**	**8**	**15**	**22**	**29**	**36**	**43**	**50**	**57**	**64**	**71**	**78**		
Complete history	X	X				X				X				X	
Physical examination[a]	X	X				X				X				X	X
Performance status	X														

Document all measurable disease (if present)	X														
Concomitant medications[a]	X	X				X				X				X	
CBC with differential[b]	X	X	X	X	X	X	X	X	X	X	X	X	X	X	X
Creatinine, total bilirubin, ALT, or AST[b]	X	X	X	X	X	X	X	X	X	X	X	X	X	X	X
TSH, free T3, free T4, total cortisol[c]		X				X				X				X	
EKG	X					X				X				X	
Respiratory PCR panel	X														
Urine analysis	X														
Pregnancy test[d]	X														
Bone marrow aspirate/biopsy[e]	X					X								X	
Respiratory PCR panel and Chest XRay	X														
**Correlative**		**Cycle 1**				**Cycle 2**				**Cycle 3**				**Subsequent cycles**	**EOS**
Optional correlatives on blood[f]		Day 1		Day 14		Day 1		Day 14		Day 1		Day 14		Day 1 and 14	
Optional correlatives on bone marrow[g]		Day 1			Day 28									Every 1-3 cycles	

[a]*A complete physical examination and documentation of concomitant medications will be done on day 1 of each cycle (+/- 4 days).*
[b]*CBC with differential, creatinine, total bilirubin, ALT or AST will be done at least once weekly (+/- 4 days) for the first 3 cycles, then every 2-4 weeks on subsequent cycles.*
[c]*TSH, free T3, free T4, total cortisol will be done at least once monthly (+/- 4 days) after the start of therapy for the first 3 cycles, then every 3 cycles (+/- 4 days) on subsequent cycles.*
[d]*Pregnancy test either urine or plasma should be done in women of childbearing potential 72 hours*

before initiation of protocol therapy.

[e]*Bone marrow aspiration must be done within 28-days of initiation of therapy. Cytogenetics may be used from prior bone marrow analysis if these were not reported on the screening bone marrow.*
[f]*Correlative studies will be collected on peripheral blood at baseline (prior to 5-azacytidine dose), on day 14 (prior to nivolumab), and between days 21 to 28 on cycle 1. Subsequently, peripheral blood will be obtained on day 1 and day 14 of each cycles, and at progression if possible. All these tests can be +/- 3 days*
[g]*Correlative studies will be collected on bone marrow at day 28 (+/-7 days), then every 1-3 cycles, and at progression.*
[h]*EOS visitis include a physical examination, CBC with differential and platelets and a limited chemistry profile (total bilirubin, serum creatinine, SGPT or SGOT). A bone marrow aspiration may be done if non- response or progressive disease cannot be unequivocally diagnosed from peripheral blood.*

Data regarding adverse events will be collected during the study. Please see **Appendix D** regarding data capturing of adverse events and adverse events source documentation. Protocol specific data will be entered into PDMS/CORe. PDMS/CORe will be used as the electronic case report form for this protocol. Only unexpected AEs will be recorded in the Case Report Form (CRF). The Principal Investigator will sign and date the AE log per each patient at the completion of each course. Following signature, the AE log will be used as source documentation for the adverse events for attribution.

Treatment may be discontinued for a variety of reasons, including patient withdrawal, investigator decision, and reasons specified by the protocol. Reasons for discontinuation of treatments are described below.

8.1 CRITERIA FOR RESPONSE:

Response Criteria for AML

Response criteria will be modified from the International Working Group for AML [67]. Responders are patients who obtain a CR, CRi, or PR, with or without cytogenetic response, hematologic improvements, and morphologic leukemia-free state. Briefly, criteria are as follows:

8.1.1 Complete remission (CR):

- Peripheral blood counts:
  No circulating blasts
  Neutrophil count >/=1.0 x10^9/L
  Platelet count >/=100 x10^9/L

- Bone marrow aspirate and biopsy:
  ≤5% blasts
  No Auer rods
  No extramedullary leukemia

8.1.2 Complete Remission with Incomplete Platelet Recovery (CRp):

For patients to be classified as being in CRp, they must achieve CR except for incomplete platelet recovery ($<100 \times 10^9$/L).

8.1.3 Complete remission with incomplete blood count recovery (CRi):

- **Peripheral blood counts:**
  No circulating blasts
  Neutrophil count <1.0 x10^9/L, or
  Platelet count <100 x10^9/L

- **Bone marrow aspirate and biopsy:**
  </= 5% blasts
  No Auer rods
  No extramedullary leukemia

8.1.4 Partial remission:

- All CR criteria if abnormal before treatment except:
- >/=50 % reduction in bone marrow blast but still >5%

8.1.5 Morphologic leukemia-free state:

- Bone marrow: </=5% myeloblasts

8.1.6 Hematologic Improvement (HI): Hematologic response must be described by the number of positively affected cell lines.

- **Erythroid response (E)** (pretreatment Hgb <11 g/dL)
  Hgb increase by >/=1.5 g/dL

- **Platelet response (P)** (pretreatment platelets <100 x10^9/L)
  Absolute increase of >/=30 x 10^9/L for patients starting with > 20 x 10^9/L platelets
  Increase from < 20 x 10^9/L to > 20 x 10^9/L and by at least 100%

- **Neutrophil response (N)** (pretreatment ANC <1.0 x10^9/L)
  At least 100% increase and an absolute increase > 0.5 x 10^9/L

- **Blast response (B)**
  >/=50% reduction in peripheral blood or bone marrow blasts but still >5%

9 DISCONTINUATION OF TREATMENT:

9.1 Discontinuation Criteria for Individual Patients

9.1.1 Patient Withdrawal

Patients may voluntarily withdraw consent to participate in the clinical study at any time and without giving any reason. Their withdrawal will not jeopardize their relationship with their healthcare providers or affect their future care. Patients may also choose to withdraw from study treatment, but agree to remain in the study for follow-up procedures.

9.1.2 Investigator Discontinuation of Patient

The investigator may exercise medical judgment to discontinue study treatment if clinically significant changes in clinical status or laboratory values are noted.

9.1.3 Criteria for Protocol-Defined Required Discontinuation of Treatment

The protocol requires discontinuation of study treatment for the following reasons:

1. Patient requests discontinuation.
2. Unacceptable toxicity that in the opinion of the investigator makes it unsafe to continue therapy.
3. Clinically significant progressive disease.
4. Investigator discretion.

9.1.4 Follow-Up at Treatment Discontinuation or Early Withdrawal

Patients who discontinue treatment for any reason should complete end-of-treatment procedures when possible. End of treatment procedures will include a physical examination, CBC with differential and platelets and a limited chemistry profile (total bilirubin, serum creatinine, SGPT or SGOT). A bone marrow aspiration may be recommended only if non-response or progressive disease cannot be unequivocally diagnosed from peripheral blood. Although treatment will be discontinued at that time, all patients who do not withdraw consent for follow-up, die, or become lost to follow-up, will remain on study for follow-up evaluations. Subject will be followed for toxicity for at least 30 days after the last protocol treatment. The 30-day follow-up visit (+ or – 3 days) will be scheduled as a clinic visits for clinical evaluation and physical examinations. If the patient cannot make it to the MDACC clinic for this visit, the required follow up treatment procedures may be done with a local physician and the records forwarded to MDACC. The research nurse will contact the patient by telephone and get a verbal assessment of the patient’s condition. The phone conversation will then be documented in the patient’s charts.

9.2 Study Stopping Rules

The principal investigator and MDACC IND office have the right to terminate this clinical study at any time. The principal investigator and MDACC IND office, as appropriate, will be involved in any decisions regarding terminating the study, temporarily suspending enrollment, or stopping ongoing treatment with study treatment. Reasons for terminating the clinical study or a study site’s participation include, but are not limited to, the following:

- The incidence or severity of an adverse reaction related to treatment in this study or other studies indicates a potential health hazard to patients
- Data recording is significantly inaccurate or incomplete
- Study site personnel are noncompliant with study procedures

- Pattern of noncompliance is observed

9.3 Protocol Violations and Deviations

Protocol violations are defined as significant departures from protocol-required processes or procedures that affect patient safety or benefit potential, or confound assessments of safety or clinical activity. A protocol deviation is a departure from the protocol that does not meet the above criteria. Protocol violations or deviations may be grouped into the following classes:

- Enrollment criteria
- Study activities (missed evaluations or visits) except for those allowed per protocol
- Noncompliance with dose or schedule, including dose calculation, administration, interruption, reduction, or delay; or discontinuation criteria
- Investigational product handling, including storage and accountability
- Informed consent and ethical issues

10 ADVERSE EVENT REPORTING

10.1 Adverse event is any untoward medical occurrence that may present during treatment with a pharmaceutical product but which does not necessarily have a causal relationship with this treatment.

Adverse drug reaction is a response to a drug which is noxious and unintended and which occurs at doses normally used in man for prophylaxis, diagnosis, or therapy of disease or for the modification of physiologic function.

Assessing causal connections between agents and disease is fundamental to the understanding of adverse drug reactions. In general, a drug may be considered a contributory cause of an adverse event if, had the drug not been administered, 1) the event would not have happened at all, 2) the event would have occurred later than it actually did, or 3) the event would have been less severe.

The Investigator or physician designee is responsible for verifying and providing source documentation for all adverse events and assigning the attribution for each event for all subjects enrolled on the trial.

10.2 **Adverse Events (AEs) will be evaluated according to the latest CTC version 4 (Appendix C) and documented in medical record. Only unexpected AEs will be recorded in the Case Report Form (CRF). Expected events during leukemia therapy are:**

10.2.6 Myelosuppression related events (due to disease or leukemia therapy)

10.2.6.1 febrile or infection episodes not requiring management in the intensive care unit

10.2.6.2 epistaxis or bleeding except for catastrophic CNS or pulmonary hemorrhage

10.2.6.3 anemia, neutropenia, lymphopenia, thrombocytopenia, leukopenia

10.2.7 Disease related events

10.2.7.1 symptoms associated with anemia

10.2.7.1.1 fatigue

10.2.7.1.2 weakness

10.2.7.1.3 shortness of breath

10.2.7.2 electrolyte abnormalities (sodium, potassium, bicarbonate, CO2, magnesium)

10.2.7.3 chemistry abnormalities (LDH, phosphorus, calcium, BUN, protein, albumin, uric acid, alkaline phosphatase, glucose)

10.2.7.4 coagulation abnormalities

10.2.7.5 disease specific therapy (induction, maintenance, salvage, or stem cell therapy)

10.2.7.6 alopecia

10.2.7.7 bone, joint, or muscle pain

10.2.7.8 liver function test abnormalities associated with infection or disease progression

10.2.7.9 disease progression

10.2.7.10 abnormal hematologic values

10.2.8 General therapy related events

10.2.8.1 catheter related events

10.2.8.2 renal failure related to tumor lysis syndrome or antibiotic/ antifungal therapy

10.2.8.3 rash related to antibiotic use

10.2.9 Hospitalization for the management of any of the above expected events

10.3 Abnormal hematologic values will not be captured in the CRFs or the Leukemia AE log. Only the highest grade per course of grade 3 or 4 chemistry lab abnormalities will be captured on the AE log as well. All Serious Adverse Events will be captured on the Leukemia AE log as well as entered in the CRFs, regardless of expectedness.

10.4 Serious Adverse Event Reporting (SAE)

An adverse event or suspected adverse reaction is considered “serious” if, in the view of either the investigator or the sponsor, it results in any of the following outcomes:

- Death
- A life-threatening adverse drug experience – any adverse experience that places the patient, in the view of the initial reporter, at immediate risk of death from the adverse experience as it occurred. It does not include an adverse experience that, had it occurred in a more severe form, might have caused death.
- Inpatient hospitalization or prolongation of existing hospitalization
- A persistent or significant incapacity or substantial disruption of the ability to conduct normal life functions.
- A congenital anomaly/birth defect.

Important medical events that may not result in death, be life-threatening, or require hospitalization may be considered a serious adverse drug experience when, based upon appropriate medical judgment, they may jeopardize the patient or subject and may require medical or surgical intervention to prevent one of the outcomes listed in this definition. Examples of such medical events include allergic bronchospasm requiring intensive treatment in an emergency room or at home, blood dyscrasias or convulsions that do not result in inpatient hospitalization, or the development of drug dependency or drug abuse (21 CFR 312.32).

- Important medical events as defined above, may also be considered serious adverse events. Any important medical event can and should be reported as an SAE if deemed appropriate by the Principal Investigator or the IND Sponsor, IND Office.

- All events occurring during the conduct of a protocol and meeting the definition of a SAE must be reported to the IRB in accordance with the timeframes and procedures outlined in “The University of Texas M. D. Anderson Cancer Center Institutional Review Board Policy for Investigators on Reporting Unanticipated Adverse Events for Drugs and Devices”. Unless stated otherwise in the protocol, all SAEs, expected or

  unexpected, must be reported to the IND Office, regardless of attribution (within 5 working days of knowledge of the event).

- **All life-threatening or fatal events,** that are unexpected, and related to the study drug, must have a written report submitted within **24 hours** (next working day) of knowledge of the event to the Safety Project Manager in the IND Office.

- Unless otherwise noted, the electronic SAE application (eSAE) will be utilized for safety reporting to the IND Office and MDACC IRB.

- Serious adverse events will be captured from the time of the first protocol-specific intervention, until 30 days after the last dose of drug, unless the participant withdraws consent. Additionally, any serious adverse events that occur between Day 31-100 will be captured as immune mediated toxicities from checkpoint inhibitors may have delayed manifestations. Serious adverse events must be followed until clinical recovery is complete and laboratory tests have returned to baseline, progression of the event has stabilized, or there has been acceptable resolution of the event.

- Additionally, any serious adverse events that occur after the 30 day time period that are related to the study treatment must be reported to the IND Office. This may include the development of a secondary malignancy.

Reporting to FDA:

- Serious adverse events will be forwarded to FDA by the IND Sponsor (Safety Project Manager IND Office) according to 21 CFR 312.32.

It is the responsibility of the PI and the research team to ensure serious adverse events are reported according to the Code of Federal Regulations, Good Clinical Practices, the protocol guidelines, the sponsor's guidelines, and Institutional Review Board policy

10.5 Serious Adverse event Reporting to Bristol-Myers Squibb Inc.

All Serious Adverse Events must be reported to BMS Worldwide Safety

- All SAEs, whether related or unrelated to nivolumab and all pregnancies must be reported to BMS (by the investigator or designee) within 24 hours.
- All SAEs should be reported via confirmed facsimile (fax) transmission, or scanned and reported via electronic mail to:
  SAE Email Address: Worldwide.Safety@BMS.com
  SAE Fax Number: 609-818-3804

11.1 STATISTICAL CONSIDERATIONS

This will be a phase II, two parallel arm, open-label, non-randomized study with a safety lead-in phase.

11.2 Sample Size

Arm 1:

Part a. Lead-in phase

The primary objective of the lead-in phase is to determine the safety of nivolumab in combination with 5-azacytidine in patients with refractory/ relapsed AML. Up to 30 patients will be accrued into the lead-in part of the study.

Part b. Phase II

A. Up to 70 patients (including 6 patients treated at the MTD from the lead-in part) with relapsed/refractory AML will be recruited for the Arm 1 Salvage cohort .

B. Up to 40 newly diagnosed older AML patients (age ≥ 65) will be recruited for the Arm 1 Frontline older cohort .

Arm 2:

Part a. Lead-in phase

The primary objective of the lead-in phase is to determine the safety of nivolumab with ipilimumab in combination with 5-azacytidine in patients with refractory/ relapsed AML. Up to 24 patients will be accrued into the lead-in part of the study.

Part b. Phase II

A. Up to 30 patients (including 6 patients treated at the MTD from the lead-in part) with relapsed/refractory AML will be recruited for the Arm 2 Salvage cohort .

B. Up to 30 newly diagnosed older AML patients (age ≥ 65) will be recruited for the Arm 2 Frontline older cohort.

The efficacy of the combination will be measured by the overall response rate (ORR), defined as CR (complete remission) + CRp (complete remission with incomplete platelet recovery) + CRi (complete remission with incomplete count recovery) + PR (partial response) + marrow clearance of blasts + hematologic improvement (HI) in platelets, hemoglobin, or ANC within 3 months of treatment initiation among adult patients with refractory/ relapsed AML AND newly diagnosed older patients with AML. ORR and toxicity will be monitored simultaneously using the Bayesian approach of Thall, Simon, Estey (1995, 1996) and the extension by Thall and Sung (1998).

Once safety of the MTD has been established, any patients still on study at a dose lower than MTD can be dose escalated up to MTD. Patients treated in the lead-in phase at MTD will be included in the Phase II portion of the study.

11.3 Statistical Design

Part a. Lead-in phase

The MTD is defined as the highest dose level with < 2 out of 6 patients experience a DLT during the first 28 days of treatment. DLT is defined in section 5.2.2. The dosing schema for the combination treatment during the lead in phase is shown in Table 5 and 5B in section 5.2.1.

Part b. Phase II

A. Arm 1 Relapsed/Refractory AML cohort (Aza+Nivo) (N=70: completed accrual)

Historical data on similar patients show a ORR of 10-15%. The target ORR with the experimental treatment is 30%. This regimen of the combination treatment will be considered worthy of further investigation if it elicits an increase in ORR to 30% with acceptable toxicity. A >30% drug-related grade 3/4 toxicity rate is considered unacceptable. Thus, interim monitoring rules, assuming the prior distributions below, were constructed that meet the following two conditions,

1) Stop if Prob{ p(ORR,E)<p(ORR,H) + 0.15 | data} > 0.99, or

2) Stop if Prob{ p(TOX,E)>0.30| data}>0.95,

Where P(ORR, E) and P(TOX, E) are the true ORR and toxicity rates for the combination treatment, and p(ORR, H) is the true ORR rate of the standard treatment. The first rule provides for stopping the study if the data suggest that it is unlikely (i.e., probability < 1%) that ORR rate of the combination treatment is greater than the ORR rate of standard treatment by 15.0%. The second condition will stop the study early if excessive therapy-related grade 3/4 toxicity (>30%) is highly probable (i.e., probability >95.0%) for the combination treatment. Monitoring for toxicity and futility will not begin until 6 patients have been evaluated, and cohort size for future evaluations is 3.

The monitoring rule for the toxicity rate, based on these assumptions and monitoring conditions above is found in Table 9. **For example, accrual will cease if 5 or more patients experience toxicities among the first 6 patients.**

Table 9. Stop accrual if the number of drug-related grade 3/4 toxicities is greater than or equal to indicated (i.e., # patients with toxicities) among the number of patients evaluated

# patients evaluated	3	6	9	12	15	18	21
# patients with toxicities	3	5-6	6-9	7-12	8-15	9-18	11-21
# patients evaluated	24	27	30	33	36	39	42
# patients with toxicities	12-24	13-27	14-30	15-33	16-36	17-39	18-42
# patients evaluated	45	48	61	54	57	60	63
# patients with toxicities	19-45	20-48	22-51	23-54	24-57	25-60	26-63
# patients evaluated	66	70					
# patients with toxicities	27-66	Always stop with this many patients					

Monitoring the ORR rate, based on the above assumptions and monitoring conditions is found in Table 10. **For example, accrual will cease if no patient experience an overall response within 3 months of initiation of therapy in the first 9 patients treated.**

Table 10. Stop accrual if the number with overall response is less than or equal to indicated (i.e., # patients with overall response) among the number of patients evaluated

# patients evaluated	3-6	9-12	15-18	21-24	27-30	33	36-39
# patients with overall response	Never stop with this many patients	0	1	2	3	4	5
# patients evaluated	42	45-48	51	54-57	60	63-66	70

# patients with overall response	6	7	8	9	10	11	Always stop with this many patients

Multc Lean Desktop (version 2.1.0) was used to generate the toxicity and futility stopping boundaries and the OC table (Table 11). In order to utilize the software for the design, a 15% constant rate and beta(0.3, 1.7) priors were assumed for the standard treatment response distribution and experimental treatment response prior distribution, respectively. In addition, a 30% toxicity constant rate and beta (0.6, 1.4) priors were assumed for the standard treatment toxicity constant rate and experimental treatment toxicity prior distribution, respectively.

The probability of stopping the study early if the true ORR of the combination treatment was 30% and the true toxicity rate was 30% was 24.7%. Probabilities of stopping early for high true toxicity rates (i.e., 50%) were 97.5% when the true ORR was 30% and 99.6% when true ORR rate was 15%.

Table 11. Operating characteristics for simultaneous monitoring response and toxicity rates for patients treated with combination treatment

True Toxicity Rate	True ORR	Prob(stop the trial early)
0.10	0.15	0.8503
	0.20	0.5344
	0.25	0.2466
	0.30	0.0967
	0.35	0.0377
	0.40	0.0157
0.20	0.15	0.8527
	0.20	0.5419
	0.25	0.2587
	0.30	0.1112
	0.35	0.0532
	0.40	0.0315
0.30	0.15	0.8752
	0.20	0.6118
	0.25	0.3718
	**0.30**	**0.2469**
	0.35	0.1977
	0.40	0.1793
0.40	0.15	0.9500
	0.20	0.8446
	0.25	0.7485
	0.30	0.6985
	0.35	0.6788
	0.40	0.6714
0.50	0.15	0.9959

	0.20	0.9872

Table 11. Operating characteristics for simultaneous monitoring response and toxicity rates for patients treated with combination treatment

True Toxicity Rate	True ORR	Prob(stop the trial early)
	0.25	0.9792
	0.30	0.9751
	0.35	0.9735
	0.40	0.9729

B. Arm 1 Newly diagnosed older AML (≥65 years) cohort (Aza+Nivo) (N=40)

Historical data on similar patients show an ORR of 18-25% [the historical response rate in older newly diagnosed AML (≥65 years) with single agent hypomethylator therapy (5-azacytidine or decitabine) in three large randomized phase II trials was 17.5 % with decitabine alone in patients with AML (Kantarjian et al., JCO 2012), 18% with azacytidine alone in patients with AML and blasts 20-30% (Fenaux et al., JCO 2010), and 27.8% with azacytidine alone in patients with AML and blasts >30% (Dombret et al., Blood 2015)]. The target ORR with the experimental treatment is 40%. This regimen of the combination treatment will be considered worthy of further investigation if it elicits an increase in ORR to 40% with acceptable toxicity. A >30% drug-related grade 3/4 toxicity rate is considered unacceptable. Thus, interim monitoring rules, assuming the prior distributions below, were constructed that meet the following two conditions,

3) Stop if Prob{ p(ORR,E)<p(ORR,H) + 0.15 | data} > 0.99, or

4) Stop if Prob{ p(TOX,E)>0.30| data}>0.95,

where P(ORR, E) and P(TOX, E) are the true ORR and toxicity rates for the combination treatment, and p(ORR, H) is the true ORR rate of the standard treatment. The first rule provides for stopping the study if the data suggest that it is unlikely (i.e., probability < 1%) that ORR rate of the combination treatment is greater than the ORR rate of standard treatment by 15.0%. The second condition will stop the study early if excessive therapy-related grade 3/4 toxicity (>30%) is highly probable (i.e., probability >95.0%) for the combination treatment. Monitoring for toxicity and futility will not begin until 6 patients have been evaluated, and cohort size for future evaluations is 4.

The monitoring rule for the toxicity rate, based on these assumptions and monitoring conditions above is found in Table 12. **For example, accrual will cease if 5 or more patients experience toxicities among the first 8 patients.**

Table 12. Stop accrual if the number of drug-related grade 3/4 toxicities is greater than or equal to indicated (i.e., # patients with toxicities) among the number of patients evaluated

# patients evaluated	4	8	12	16	20	24	28	32	36	40
# patients with toxicities	4	5-8	7-12	9-16	10-20	12-24	13-28	15-32	16-36	Always stop with this many patients

Monitoring the ORR rate, based on the above assumptions and monitoring conditions is found in Table 13. **For example, accrual will cease if no patient experience an overall**

response within 3 months of initiation of therapy in the first 8 patients treated.

Table 13. Stop accrual if the number with overall response is less than or equal to indicated (i.e., # patients with overall response) among the number of patients evaluated									
# patients evaluated	4	8	12	16	20	24	28	32	36	40
# patients with overall response	Never stop with this many patients	0	0-1	0-2	0-3	0-4	0-5	0-6	0-8	Always stop with this many patients

Multc Lean Desktop (version 2.1.0) was used to generate the toxicity and futility stopping boundaries and the OC table (Table 14). In order to utilize the software for the design, a 25% constant rate, and beta(0.5,1.5) priors were assumed for the standard treatment response distribution and experimental treatment response prior distribution, respectively. In addition, a 30% toxicity constant rate and beta (0.6, 1.4) priors were assumed for the standard treatment toxicity constant rate and experimental treatment toxicity prior distribution, respectively.

The probability of stopping the study early if the true ORR of the combination treatment was 30% and the true toxicity rate was 30% was 17.6%. Probabilities of stopping early for high true toxicity rates (i.e., 50%) were 86.5% when the true ORR was 40% and 93.1% when true ORR rate was 25%.

Table 14. Operating characteristics for simultaneous monitoring response and toxicity rates for patients treated with combination treatment

True Toxicity Rate	True ORR	Prob(stop the trial early)
0.10	0.25	0.5185
	0.35	0.1345
	0.40	0.0558
	0.45	0.0217
	0.50	0.0083
0.20	0.25	0.5254
	0.35	0.2975
	0.40	0.0694
	0.45	0.0358
	0.50	0.0226
0.30	0.25	0.5797
	0.35	0.2446
	**0.40**	**0.1759**
	0.45	0.1461
	0.50	0.1344
0.40	0.25	0.7494
	0.35	0.5496
	0.40	0.5087
	0.45	0.4909

Table 14. Operating characteristics for simultaneous monitoring response and toxicity rates for patients treated with combination treatment		
True Toxicity Rate	True ORR	Prob(stop the trial early)
	0.50	0.4840
0.50	0.25	0.9309
	0.35	0.8759
	0.40	0.8646
	0.45	0.8597
	0.50	0.8578

C. Arm 2 First and Second Salvage AML cohort (AZA + Nivo + Ipi) (N=30)

Historical data on similar patients show an ORR of 12-15%. The target ORR with the experimental treatment is 30%. This regimen of the combination treatment will be considered worthy of further investigation if it elicits an increase in ORR to 30% with acceptable toxicity. A >30% drug-related grade 3/4 toxicity rate is considered unacceptable. Thus, interim monitoring rules, assuming the prior distributions below, were constructed that meet the following two conditions,

5) Stop if Prob{ p(ORR,E)<p(ORR,H) + 0.15 | data} > 0.99, or

6) Stop if Prob{ p(TOX,E)>0.30| data}>0.95,

Where P(ORR, E) and P(TOX, E) are the true ORR and toxicity rates for the combination treatment, and p(ORR, H) is the true ORR rate of the standard treatment. The first rule provides for stopping the study if the data suggest that it is unlikely (i.e., probability < 1%) that ORR rate of the combination treatment is greater than the ORR rate of standard treatment by 15.0%. The second condition will stop the study early if excessive therapy-related grade 3/4 toxicity (>30%) is highly probable (i.e., probability >95.0%) for the combination treatment. Monitoring for toxicity and futility will not begin until 6 patients have been evaluated, and cohort size for future evaluations is 3.

The monitoring rule for the toxicity rate, based on these assumptions and monitoring conditions above is found in Table 15. **For example, accrual will cease if 5 or more patients experience toxicities among the first 6 patients.**

Table 15. Stop accrual if the number of drug-related grade 3/4 toxicities is greater than or equal to indicated (i.e., # patients with toxicities) among the number of patients evaluated										
# patients evaluated	3	6	9	12	15	18	21	24	27	30
# patients with toxicities	Never stop with this many patients	5-6	6-9	7-12	8-15	9-18	11-21	12-24	13-27	14-30

Monitoring the ORR rate, based on the above assumptions and monitoring conditions is found in Table 16. **For example, accrual will cease if no patient experience an overall response within 3 months of initiation of therapy in the first 9 patients treated.**

Table 16. Stop accrual if the number with overall response is less than or equal to indicated (i.e., # patients with overall response) among the number of patients evaluated						
# patients evaluated	3-6	9-12	15-18	21-24	27	30
# patients with overall response	Never stop with this many patients	0	1	2	3	Always stop with this many patients

Multc Lean Desktop (version 2.1.0) was used to generate the toxicity and futility stopping boundaries and the OC table (Table 17). In order to utilize the software for the design, a 15% constant rate and beta(0.3, 1.7) priors were assumed for the standard treatment response distribution and experimental treatment response prior distribution, respectively. In addition, a 30% toxicity constant rate and beta (0.6, 1.4) priors were assumed for the standard treatment toxicity constant rate and experimental treatment toxicity prior distribution, respectively.

The probability of stopping the study early if the true ORR of the combination treatment was 30% and the true toxicity rate was 30% was 16.5%. Probabilities of stopping early for high true toxicity rates (i.e., 50%) were 76.3% when the true ORR was 30% and 88.0% when true ORR rate was 15%.

Table 17. Operating characteristics for simultaneous monitoring response and toxicity rates for patients treated with combination treatment		
True Toxicity Rate	True ORR	Prob(stop the trial early)
0.10	0.15	0.5293
	0.20	0.3022
	0.25	0.1544
	0.30	0.0731
	0.35	0.0329
	0.40	0.0142
0.20	0.15	0.5339
	0.20	0.3090
	0.25	0.1626
	0.30	0.0821
	0.35	0.0422
	0.40	0.0238
0.30	0.15	0.5759
	0.20	0.3712
	0.25	0.2380
	**0.30**	0.1647
	0.35	0.1285
	0.40	0.1117
0.40	0.15	0.7073
	0.20	0.5661
	0.25	0.4742

	0.30	0.4236
	0.35	0.3986
	0.40	0.3870
0.50	0.15	0.8797
	0.20	0.8216
	0.25	0.7838
	0.30	0.7631
	0.35	0.7528
	0.40	0.7480

D. Arm 2 Newly diagnosed older AML (≥65 years) cohort (Aza+Nivo+Ipi) (N=30)

Historical data on similar patients show an ORR of 18-25% [the historical response rate in older newly diagnosed AML (≥65 years) with single agent hypomethylator therapy (5-azacytidine or decitabine) in three large randomized phase II trials was 17.5 % with decitabine alone in patients with AML (Kantarjian et al., JCO 2012), 18% with azacytidine alone in patients with AML and blasts 20-30% (Fenaux et al., JCO 2010), and 27.8% with azacytidine alone in patients with AML and blasts >30% (Dombret et al., Blood 2015)]. The target ORR with the experimental treatment is 40%. This regimen of the Aza+Nivo+Ipi combination treatment will be considered worthy of further investigation if it elicits an increase in ORR to 40% with acceptable toxicity. A >30% drug-related grade 3/4 toxicity rate is considered unacceptable. Thus, interim monitoring rules, assuming the prior distributions below, were constructed that meet the following two conditions,

7) Stop if Prob{ p(ORR,E)<p(ORR,H) + 0.15 | data} > 0.99, or

8) Stop if Prob{ p(TOX,E)>0.30| data}>0.95,

where P(ORR, E) and P(TOX, E) are the true ORR and toxicity rates for the combination treatment, and p(ORR, H) is the true ORR rate of the standard treatment. The first rule provides for stopping the study if the data suggest that it is unlikely (i.e., probability < 1%) that ORR rate of the combination treatment is greater than the ORR rate of standard treatment by 15.0%. The second condition will stop the study early if excessive therapy-related grade 3/4 toxicity (>30%) is highly probable (i.e., probability >95.0%) for the combination treatment. Monitoring for toxicity and futility will not begin until 6 patients have been evaluated, and cohort size for future evaluations is 3.

The monitoring rule for the toxicity rate, based on these assumptions and monitoring conditions above is found in Table 18. **For example, accrual will cease if 5 or more patients experience toxicities among the first 6 patients.**

Table 18. Stop accrual if the number of drug-related grade 3/4 toxicities is greater than or equal to indicated (i.e., # patients with toxicities) among the number of patients evaluated										
# patients evaluated	3	6	9	12	15	18	21	24	27	30
# patients with toxicities	Never stop with this many patients	5-6	6-9	7-12	8-15	9-18	11-21	12-24	13-27	Always stop with this many patients

Monitoring the ORR rate, based on the above assumptions and monitoring conditions is found in Table 19. **For example, accrual will cease if no patient experience an overall response within 3 months of initiation of therapy in the first 8 patients treated.**

Table 19. Stop accrual if the number with overall response is less than or equal to indicated (i.e., # patients with overall								
# patients	3	6-9	12	15-18	21	24	27	30
# patients with overall response	Never stop with this many patients	0	1	2	3	4	5	Always stop with this many patients

Multc Lean Desktop (version 2.1.0) was used to generate the toxicity and futility stopping boundaries and the OC table (Table 19). In order to utilize the software for the design, a 25% constant rate, and beta(0.5,1.5) priors were assumed for the standard treatment response distribution and experimental treatment response prior distribution, respectively. In addition, a 30% toxicity constant rate and beta (0.6, 1.4) priors were assumed for the standard treatment toxicity constant rate and experimental treatment toxicity prior distribution, respectively.

The probability of stopping the study early if the true ORR of the combination treatment was 30% and the true toxicity rate was 30% was 17.6%. Probabilities of stopping early for high true toxicity rates (i.e., 50%) were 86.5% when the true ORR was 40% and 93.1% when true ORR rate was 25%.

Table 19. Operating characteristics for simultaneous monitoring response and toxicity rates for patients treated with combination treatment		
True Toxicity Rate	True ORR	Prob(stop the trial early)
0.10	0.25	0.4344
	0.30	0.2594
	0.35	0.1429
	0.40	0.0751
	0.45	0.0384
	0.50	0.0194
0.20	0.25	0.4399
	0.30	0.2665
	0.35	0.1512
	0.40	0.0840
	0.45	0.0477
	0.50	0.0289
0.30	0.25	0.4903
	0.30	0.3326

	0.35	0.2277
	**0.40**	**0.1665**
	0.45	0.1335
	0.50	0.1163
0.40	0.25	0.6483
	0.30	0.5395
	0.35	0.4671
	0.40	0.4249
	0.45	0.4021
	0.50	0.3902
0.50	0.25	0.8554
	0.30	0.8107
	0.35	0.7809
	0.40	0.7636
	0.45	0.7542
	0.50	0.7493

Statistical Analysis Plan

All patients who received any dose of the study agent will be included in the analysis for efficacy and safety. Demographic/clinical characteristics (including duration of response) and safety data of the patients will be summarized using descriptive statistics such as mean, standard deviation, median and range. For the primary efficacy analysis, we will estimate the ORR for the combination treatment, along with the 95% confidence interval. Patients who drop out of the study before completing all the cycles will be treated as "failures" for the primary analysis. Overall response rate (ORR) during the study period will also be presented with the 95% confidence interval. The association between ORR and patient's clinical characteristics will be examined by Wilcoxon's rank sum test or Fisher's exact test, as appropriate. Toxicity type, severity and attribution will be summarized for each patient using frequency tables.

The distribution of time-to-event endpoints (DFS and OS) including overall survival and progression free survival will be estimated using the method of Kaplan and Meier. Comparisons of time-to-event endpoints by important subgroups will be made using the log-rank tests. Paired t-tests will be used to determine the immunological and molecular changes in the peripheral blood and bone marrow from baseline to the time of response, and to the time of disease progression. Correlation analysis (such as logistic regression analysis) will be conducted to determine the relationship between induction of hypomethylation / DNA damage and clinical response.

Statistical analysis of biomarker data: Descriptive statistics including plots, mean, median and standard deviations will be used to summarize data. For continuous outcomes, t-test and ANOVA will be used to compare outcome measures across patient characteristics. Dunnett's and Tukey's test that properly adjust for multiplicity in multiple tests will be implemented. Pair-wise comparisons will be performed using pre- and post-therapy samples from each patient . The chi-square test or Fisher's exact test will be used to test the

association between two categorical variables such as disease state and performance status. Both univariate and multivariate logistic regressions will be performed to model prognostic factors.

12.0 PROTOCOL ADMINISTRATION

This study will be monitored for compliance by the IND Office.

Protocol amendments

Changes to the protocol will be made only when protocol amendments have been signed by the principal investigator and approved by the sponsor and the IRB of the study center.

Archival of data

All patient data (including source data) generated in connection with this study will be kept in the archives of the MDACC for at least 15 years after the study has been completed. All data will be available for inspection by company representatives of the Medical Department and by regulatory authorities.

13.0 REFERENCES

1. Siegel R, Naishadham D, Jemal A. Cancer statistics, 2013. CA: a cancer journal for clinicians 2013;63:11-30.
2. Heerema-McKenney A, Arber DA. Acute myeloid leukemia. Hematology/oncology clinics of North America 2009;23:633-654.
3. Appelbaum FR, Gundacker H, Head DR, et al. Age and acute myeloid leukemia. Blood 2006;107:3481-3485.
4. Kantarjian HM. Therapy for elderly patients with acute myeloid leukemia: a problem in search of solutions. Cancer 2007;109:1007-1010.
5. Kantarjian H, O'Brien S, Cortes J, et al. Results of intensive chemotherapy in 998 patients age 65 years or older with acute myeloid leukemia or high-risk myelodysplastic syndrome: predictive prognostic models for outcome. Cancer 2006;106:1090-1098.
6. Estey EH. Treatment of relapsed and refractory acute myelogenous leukemia. Leukemia 2000;14:476-479.
7. Ravandi F, Cortes J, Faderl S, et al. Characteristics and outcome of patients with acute myeloid leukemia refractory to 1 cycle of high-dose cytarabine-based induction chemotherapy. Blood 2010;116:5818-5823; quiz 6153.
8. Stanisic S, Kalaycio M. Treatment of refractory and relapsed acute myelogenous leukemia. Expert Rev Anticancer Ther 2002;2:287-295.
9. Dong H, Zhu G, Tamada K, et al. B7-H1, a third member of the B7 family, co-stimulates T-cell proliferation and interleukin-10 secretion. Nature medicine 1999;5:1365-1369.
10. Blank C, Brown I, Peterson AC, et al. PD-L1/B7H-1 inhibits the effector phase of tumor rejection by T cell receptor (TCR) transgenic CD8+ T cells. Cancer research 2004;64:1140-1145.
11. Curiel TJ, Wei S, Dong H, et al. Blockade of B7-H1 improves myeloid dendritic

cell-mediated antitumor immunity. Nature medicine 2003;9:562-567.
12. Hirano F, Kaneko K, Tamura H, et al. Blockade of B7-H1 and PD-1 by monoclonal antibodies potentiates cancer therapeutic immunity. Cancer research 2005;65:1089-1096.
13. Blank C, Mackensen A. Contribution of the PD-L1/PD-1 pathway to T-cell exhaustion: an update on implications for chronic infections and tumor evasion. Cancer immunology, immunotherapy : CII 2007;56:739-745.
14. Loos M, Giese NA, Kleeff J, et al. Clinical significance and regulation of the costimulatory molecule B7-H1 in pancreatic cancer. Cancer letters 2008;268:98-109.
15. Gao Q, Wang XY, Qiu SJ, et al. Overexpression of PD-L1 significantly associates with tumor aggressiveness and postoperative recurrence in human hepatocellular carcinoma. Clinical cancer research : an official journal of the American Association for Cancer Research 2009;15:971-979.
16. Keir ME, Butte MJ, Freeman GJ, et al. PD-1 and its ligands in tolerance and immunity. Annual review of immunology 2008;26:677-704.
17. Zhang L, Gajewski TF, Kline J. PD-1/PD-L1 interactions inhibit antitumor immune responses in a murine acute myeloid leukemia model. Blood 2009;114:1545-1552.

18. Liu J, Hamrouni A, Wolowiec D, et al. Plasma cells from multiple myeloma patients express B7-H1 (PD-L1) and increase expression after stimulation with IFN-{gamma} and TLR ligands via a MyD88-, TRAF6-, and MEK-dependent pathway. Blood 2007;110:296-304.
19. Yang W, Song Y, Lu YL, et al. Increased expression of programmed death (PD)-1 and its ligand PD-L1 correlates with impaired cell-mediated immunity in high-risk human papillomavirus-related cervical intraepithelial neoplasia. Immunology 2013;139:513-522.
20. Chen X, Liu S, Wang L, et al. Clinical significance of B7-H1 (PD-L1) expression in human acute leukemia. Cancer biology & therapy 2008;7:622-627.
21. Zhou Q, Munger ME, Veenstra RG, et al. Coexpression of Tim-3 and PD-1 identifies a CD8+ T-cell exhaustion phenotype in mice with disseminated acute myelogenous leukemia. Blood 2011;117:4501-4510.
22. Nishimura H, Okazaki T, Tanaka Y, et al. Autoimmune dilated cardiomyopathy in PD-1 receptor-deficient mice. Science 2001;291:319-322.
23. Nishimura H, Nose M, Hiai H, et al. Development of lupus-like autoimmune diseases by disruption of the PD-1 gene encoding an ITIM motif-carrying immunoreceptor. Immunity 1999;11:141-151.
24. Nomi T, Sho M, Akahori T, et al. Clinical significance and therapeutic potential of the programmed death-1 ligand/programmed death-1 pathway in human pancreatic cancer. Clinical cancer research : an official journal of the American Association for Cancer Research 2007;13:2151-2157.
25. Iwai Y, Terawaki S, Honjo T. PD-1 blockade inhibits hematogenous spread of poorly immunogenic tumor cells by enhanced recruitment of effector T cells. International immunology 2005;17:133-144.
26. Iwai Y, Ishida M, Tanaka Y, et al. Involvement of PD-L1 on tumor cells in the escape from host immune system and tumor immunotherapy by PD-L1 blockade. Proceedings of the National Academy of Sciences of the United States of America 2002;99:12293-12297.
27. Topalian SL, Hodi FS, Brahmer JR, et al. Safety, activity, and immune correlates

of anti-PD-1 antibody in cancer. The New England journal of medicine 2012;366:2443-2454.
28. Jones PA, Taylor SM, Wilson VL. Inhibition of DNA methylation by 5-azacytidine. Recent Results Cancer Res 1983;84:202-211.
29. Santi DV, Garrett CE, Barr PJ. On the mechanism of inhibition of DNA-cytosine methyltransferases by cytosine analogs. Cell 1983;33:9-10.
30. Gabbara S, Bhagwat AS. The mechanism of inhibition of DNA (cytosine-5-)-methyltransferases by 5-azacytosine is likely to involve methyl transfer to the inhibitor. Biochem J 1995;307:87-92.
31. Bird AP. The relationship of DNA methylation to cancer. Cancer Surv 1996;28:87-101.
32. Jones PA, Laird PW. Cancer epigenetics comes of age. Nat Genet 1999;21:163-167.
33. Karpf AR, Jones DA. Reactivating the expression of methylation silenced genes in human cancer. Oncogene 2002;21:5496-5503.

34. Uchida T, Kinoshita T, Nagai H, et al. Hypermethylation of the $p15^{INK4B}$ gene in myelodysplastic syndromes. Blood 1997;90:1403-1409.
35. Herman JG, Latif F, Weng Y, et al. Silencing of the VHL tumor-suppressor gene by DNA methylation in renal carcinoma. Proc Natl Acad Sci U S A 1994;91:9700-9704.
36. van der Velden PA, Metzelaar-Blok JA, Bergman W, et al. Promoter hypermethylation: a common cause of reduced p16(INK4a) expression in uveal melanoma. Cancer Res 2001;61:5303-5306.
37. Dobrovic A, Simpfendorfer D. Methylation of the BRCA1 gene in sporadic breast cancer. Cancer Res 1997;57:3347-3350.
38. Hiltunen MO, Alhonen L, Koistinaho J, et al. Hypermethylation of the APC (Adenomatous Polyposis Colie) gene promoter region in human colorectal carcinoma. Int J Cancer 1997;70:644-648.
39. Merlo A, Herman JG, Mao L, et al. 5' CpG island methylation is associated with transcriptional silencing of the tumour suppressor p16/CDKN2/MTS1 in human cancers. Nat Med 1995;1:686-692.
40. Herman JG, Jen J, Merlo A, et al. Hypermethylation-associated inactivation indicates a tumor suppressor role for p15INK4B. Cancer Res 1996;56:722-727.
41. Li LH, Olin EJ, Buskirk HH, et al. Cytotoxicity and mode of action of 5-azacytidine on L1210 leukemia. Cancer Res 1970;30:2760-2769.
42. Li LH, Olin EJ, Fraser TJ, et al. Phase specificity of 5-azacytidine against mammalian cells in tissue culture. Cancer Res 1970;30:2770-2775.
43. Silverman LR. Targeting hypomethylation of DNA to achieve cellular differentiation in myelodysplastic syndromes (MDS). Oncologist 2001;6:8-14.
44. Jones PA, Taylor SM, Wilson V. DNA modification, differentiation, and transformation. The Journal of Experimental Zoology 1983;228:287-295.
45. Leone G, Teofili L, Voso MT, et al. DNA methylation and demethylating drugs in myelodysplastic syndromes and secondary leukemias. Haematologica 2002;87:1324-1341.
46. Silverman LR, Holland JF, Demakos EP, et al. Azacitidine (Aza C) in myelodysplastic syndromes (MDS), CALGB studies 8421 and 8921. Ann Hematol; 1994. p A12.
47. Robertson KD, Jones PA. DNA methylation: past, present and future directions. Carcinogenesis 2000;21:461-467.

48. Plagemann PG, Behrens M, Abraham D. Metabolism and cytotoxicity of 5-azacytidine in cultured Novikoff rat hepatoma and P388 mouse leukemia cells and their enhancement by preincubation with pyrazofurin. Cancer Res 1978;38:2458-2466.
49. Juttermann R, Li E, Jaenisch R. Toxicity of 5-aza-2'-deoxycytidine to mammalian cells is mediated primarily by covalent trapping of DNA methyltransferase rather than DNA demethylation. Proc Natl Acad Sci U S A 1994;91:11797-11801.
50. Ueno M, Katayama K, Yasoshima A, et al. 5-Azacytidine (5AzC)-induced histopathological changes in the central nervous system of rat fetuses. Exp Toxic Pathol 2002;54:91-96.
51. Glover AB, Leyland-Jones B, Chun HG, et al. Azacitidine: 10 years later. Cancer Treat Rep 1987;71:737-746.
52. Pharmion Corporation. Azacitidine Investigator's Brochure, Edition 5. Boulder, CO; 2007.

53. Marcucci G, Silverman L, Eller M, et al. Bioavailability of azacitidine subcutaneous versus intravenous in patients with the myelodysplastic syndromes. J Clin Pharmacol 2005;45:597-602.
54. Von Hoff DD, Slavik M, Muggia FM. 5-Azacytidine. A new anticancer drug with effectiveness in acute myelogenous leukemia. Ann Intern Med 1976;85:237-245.
55. Silverman LR, McKenzie DR, Peterson BL, et al. Further analysis of trials with azacitidine in patients with myelodysplastic syndrome: studies 8421, 8921, and 9221 by the cancer and leukemia group B. J Clin Oncol 2006;24:3895-3903.
56. Fenaux P, Mufti G, Santini V, et al. Azacitidine (AZA) treatment prolongs overall survival (OS) in higher-risk MDS patients compared with conventional care regimens (CCR): Results of the AZA-001 phase III study. Blood; 2007. p 250a.
57. Dong H, Strome SE, Salomao DR, et al. Tumor-associated B7-H1 promotes T-cell apoptosis: a potential mechanism of immune evasion. Nature medicine 2002;8:793-800.
58. Bontkes HJ, Ruben JM, Alhan C, et al. Azacitidine differentially affects CD4(pos) T-cell polarization in vitro and in vivo in high risk myelodysplastic syndromes. Leukemia research 2012;36:921-930.
59. Youngblood B, Oestreich KJ, Ha SJ, et al. Chronic virus infection enforces demethylation of the locus that encodes PD-1 in antigen-specific CD8(+) T cells. Immunity 2011;35:400-412.
60. Garcia-Manero G, Kadia TM, Daver N, et al. A Phase II Study Of The Combination Of Azacitidine and Lenalidomide In Patients (pts) With Higher Risk Myelodysplastic Syndromes (MDS). Blood 2013;122.
61. Garcia-Manero G, Estey EH, Jabbour E, et al. Final Report of a Phase II Study of 5-Azacitidine and Vorinostat in Patients (pts) with Newly Diagnosed Myelodysplastic Syndrome (MDS) or Acute Myelogenous Leukemia (AML) Not Eligible for Clinical Trials Because Poor Performance and Presence of Other Comorbidities. Blood 2011;118:279-279.
62. Walter RB, Medeiros BC, Gardner KM, et al. Gemtuzumab ozogamicin in combination with vorinostat and azacitidine in older patients with relapsed or refractory acute myeloid leukemia: a phase I/II study. Haematologica 2014;99:54-59.
63. Liakou CI, Kamat A, Tang DN, et al. CTLA-4 blockade increases IFNgamma-producing CD4+ICOShi cells to shift the ratio of effector to regulatory T cells in cancer patients. Proceedings of the National Academy of Sciences of the United States of America 2008;105:14987-14992.

64. Carthon BC, Wolchok JD, Yuan J, et al. Preoperative CTLA-4 blockade: tolerability and immune monitoring in the setting of a presurgical clinical trial. Clinical cancer research : an official journal of the American Association for Cancer Research 2010;16:2861-2871.
65. Chen H, Fu T, Suh WK, et al. CD4 T cells require ICOS-mediated PI3K signaling to increase T-Bet expression in the setting of anti-CTLA-4 therapy. Cancer Immunol Res 2014;2:167-176.
66. Tang DN, Shen Y, Sun J, et al. Increased Frequency of ICOS+ CD4 T Cells as a Pharmacodynamic Biomarker for Anti-CTLA-4 Therapy (vol 1, pg 229, 2013). Cancer Immunol Res 2013;1:501-501.

67. Cheson BD, Bennett JM, Kopecky KJ, et al. Revised recommendations of the International Working Group for Diagnosis, Standardization of Response Criteria, Treatment Outcomes, and Reporting Standards for Therapeutic Trials in Acute Myeloid Leukemia. Journal of clinical oncology : official journal of the American Society of Clinical Oncology 2003;21:4642-4649.
68. Matthew D et al., A MultiCenter Phase I/Ib Study of Ipilimumab for Relapsed Hematologic Malignancies after Allogeneic Hematopoietic Stem Cell Transplantation, ASH National Meeting, Dec 2015, Abstract # 860.

14.0 APPENDICES

Appendix A: Prioritization List
Appendix B: Protocol Checklist
Appendix C: CTCAE version 4.0
Appendix D: Leukemia specific AE recording and reporting guidelines
Appendix E: Generic-Nivolumab Drug Information
Appendix F: Generic-Vidaza Drug Information
Appendix G: Management algorithms for immuno-oncology drug-related adverse events
Appendix H: Outside Physician Form
Appendix I: DOSING PROCEDURE MANUAL for Lirilumab and Nivolumab Version 2.0
Appendix J: Ipilimumab IB
Appendix K: Nivolumab and Ipilimumab product information
