# Supplementary material for: Single cell T cell landscape and T cell receptor repertoire profiling of AML in context of PD-1 blockade therapy
Source: Nat Commun. 2021 Oct 18;12:6071. doi: 10.1038/s41467-021-26282-z (PMC8524723; doi:10.1038/s41467-021-26282-z)
Supplement: Supplementary file 7 — Reporting Summary [file 41467_2021_26282_MOESM7_ESM.pdf]

nature research

Corresponding author(s): Kunal Rai, Linghua Wang, Naval Daver and Andrew Futreal

Last updated by author(s): 08/02/2021

Reporting Summary

Nature Research wishes to improve the reproducibility of the work that we publish. This form provides structure for consistency and transparency in reporting. For further information on Nature Research policies, see Authors & Referees and the Editorial Policy Checklist.

Statistics

For all statistical analyses, confirm that the following items are present in the figure legend, table legend, main text, or Methods section.

n/a	Confirmed	
☐	☒	The exact sample size (*n*) for each experimental group/condition, given as a discrete number and unit of measurement
☒	☐	A statement on whether measurements were taken from distinct samples or whether the same sample was measured repeatedly
☐	☒	The statistical test(s) used AND whether they are one- or two-sided<br>*Only common tests should be described solely by name; describe more complex techniques in the Methods section.*
☒	☐	A description of all covariates tested
☐	☒	A description of any assumptions or corrections, such as tests of normality and adjustment for multiple comparisons
☐	☒	A full description of the statistical parameters including central tendency (e.g. means) or other basic estimates (e.g. regression coefficient) AND variation (e.g. standard deviation) or associated estimates of uncertainty (e.g. confidence intervals)
☐	☒	For null hypothesis testing, the test statistic (e.g. *F*, *t*, *r*) with confidence intervals, effect sizes, degrees of freedom and *P* value noted<br>*Give P values as exact values whenever suitable.*
☒	☐	For Bayesian analysis, information on the choice of priors and Markov chain Monte Carlo settings
☒	☐	For hierarchical and complex designs, identification of the appropriate level for tests and full reporting of outcomes
☐	☒	Estimates of effect sizes (e.g. Cohen's *d*, Pearson's *r*), indicating how they were calculated

Our web collection on statistics for biologists contains articles on many of the points above.

Software and code

Policy information about availability of computer code

Data collection	R version 3.5.2; Prism v8, Cell Ranger, InferCNV, GSVA, GSEA
Data analysis	All codes used were based on publically available libraries and specific packages and modules are listed in the methods section

For manuscripts utilizing custom algorithms or software that are central to the research but not yet described in published literature, software must be made available to editors/reviewers. We strongly encourage code deposition in a community repository (e.g. GitHub). See the Nature Research guidelines for submitting code & software for further information.

Data

Policy information about availability of data

All manuscripts must include a data availability statement. This statement should provide the following information, where applicable:

- Accession codes, unique identifiers, or web links for publicly available datasets
- A list of figures that have associated raw data
- A description of any restrictions on data availability

The data have been deposited in EGA database EGA ID number for 5' scRNA-seq (EGAD00001007672), scTCR-seq (EGAD00001007674) and DNA-seq (EGAD00001007671) data. The data can be accessed via https://ega-archive.org/studies/EGAS00001004894

Field-specific reporting

Please select the one below that is the best fit for your research. If you are not sure, read the appropriate sections before making your selection.

☒ Life sciences ☐ Behavioural & social sciences ☐ Ecological, evolutionary & environmental sciences

For a reference copy of the document with all sections, see nature.com/documents/nr-reporting-summary-flat.pdf

Life sciences study design

All studies must disclose on these points even when the disclosure is negative.

Sample size	8 patients for single cell study; 2 healthy donors for single cell studies. 57 for azacitidine/nivolumab clinical trial analysis. 99 patients for azacitidine-basd regimens. Samples were selected based on available bone marrows on patients in the clinical trial.
Data exclusions	No specific data was excluded
Replication	not applicable as this is based on individual patient responses to treatment.
Randomization	Not applicable. Patients were selected based on response vs no-response, and available bone marrows to be analyzed.
Blinding	No blinding was done. Patients were selected based on response vs no-response, and available bone marrows to be analyzed.

Reporting for specific materials, systems and methods

We require information from authors about some types of materials, experimental systems and methods used in many studies. Here, indicate whether each material, system or method listed is relevant to your study. If you are not sure if a list item applies to your research, read the appropriate section before selecting a response.

Materials & experimental systems

n/a	Involved in the study	
☐	☒	Antibodies
☒	☐	Eukaryotic cell lines
☒	☐	Palaeontology
☒	☐	Animals and other organisms
☐	☒	Human research participants
☐	☒	Clinical data

Methods

n/a	Involved in the study	
☒	☐	ChIP-seq
☐	☒	Flow cytometry
☒	☐	MRI-based neuroimaging

Antibodies

Antibodies used	CD34
Validation	Not applicable

Human research participants

Policy information about studies involving human research participants

Population characteristics	Patients >= 18 years of age who had failed prior therapy for AML were eligible to participate on combined azacitidine and nivolumab trial (ClinalTrials.gov identifier: NCT02397720; full protocol is included in Supplementary files).
Recruitment	Relapsed/refractory AML and MDS patients were considered for study entry
Ethics oversight	A written informed consent that was approved by the internal review board of University of Texas M D Anderson Cancer Center was obtained. The study was conducted in accordance with the Declaration of Helsinki.

Note that full information on the approval of the study protocol must also be provided in the manuscript.

Clinical data

Policy information about clinical studies

All manuscripts should comply with the ICMJE guidelines for publication of clinical research and a completed CONSORT checklist must be included with all submissions.

Clinical trial registration	NCT02397720
Study protocol	Supplementary File of the study protocol is provided
Data collection	2016-2020 conducted at MD Anderson Cancer Center. ALl bone marrows were stored in leukemia storage bank.
Outcomes	Response to treatment (CR, CRi, Stable disease, no-response). Remission rate based on bone marrow blasts <5% for CR/CRi.

Outcomes | Overall survival and disease free survival were measured from time of treatment initiation.

Flow Cytometry

Plots

Confirm that:

☒ The axis labels state the marker and fluorochrome used (e.g. CD4-FITC).

☒ The axis scales are clearly visible. Include numbers along axes only for bottom left plot of group (a 'group' is an analysis of identical markers).

☒ All plots are contour plots with outliers or pseudocolor plots.

☒ A numerical value for number of cells or percentage (with statistics) is provided.

Methodology

Sample preparation	fresh bone marrow aspirate is collected in EDTA tubes. Flow cytometry is performed within 24 hours.
Instrument	BD FACSCanto 8-color and 10-color instruments
Software	FCSEXPRESS Version 6
Cell population abundance	200,000 events collected for each sample
Gating strategy	Starting from all events the flow cytometer collected, a gate for singlets is made to exclude all doublets. Under Singlets gate, a FSC/SSC gate is drawn to exclude debris. Then a gate for nucleated cells is drawn to exclude mature red cells. Under Nucleated cells gate, a CD45 dim gate is made. Blast population(CD34-positive blasts) illustrated in supplementary 3 is under CD45 dim gate.

☒ Tick this box to confirm that a figure exemplifying the gating strategy is provided in the Supplementary Information.
